# Evaluating the Antifungal Potential of Autophagy-Related Protein 4 (ATG4) Inhibitors against Human Fungal Pathogens

**DOI:** 10.4014/jmb.2509.09002

**Published:** 2025-12-15

**Authors:** Seungmee Jung, Jongchan Woo, Hyunjin Cha, Seung-Heon Lee, Sagar Dahal, Yong-Sun Bahn, Eunsook Park

**Affiliations:** 1 Department of Molecular Biology, College of Agriculture, Life Sciences, and Natural Resources, University of Wyoming, Laramie WY 82071, USA; 2 Department of Biotechnology, College of Life Science and Biotechnology, Yonsei University, Seoul, Republic of Korea

**Keywords:** Antifungal agent, ATG4, bioluminescence resonance energy transfer, autophagy inhibitor, human fungal pathogens

## Abstract

Emerging fungal pathogens pose a significant threat to global public health. Despite the availability of antifungal agents, their clinical efficacy is increasingly challenged by the rise of fungicide-resistant strains. Therefore, identifying novel therapeutic targets and ensuring the safe application of antifungal agents are critical for advancing treatment strategies. Autophagy, a fundamental cellular process that maintains intracellular homeostasis by degrading and recycling dysfunctional proteins and organelles, is implicated in fungal pathogenicity. It indicates that inhibition of autophagy represents a promising approach for antifungal development. In this study, we evaluate the antifungal potential of autophagy inhibitors targeting the Autophagy-related protein 4 (ATG4)-mediated cleavage of Autophagy-related protein 8 (ATG8). Our findings demonstrate that ebselen and its analogs effectively inhibit ATG4 activity in *Cryptococcus neoformans*, *Aspergillus fumigatus*, and *Aspergillus niger*, exhibiting fungicidal activity against *Cryptococcus* and *Candida* species. These results provide valuable insights into novel antifungal development strategies, highlighting the therapeutic potential of autophagy inhibitors against diverse pathogenic fungi.

## Introduction

Invasive fungal infections are one of the most challenging diseases to manage in human health today [1-3]. The three most common systemic fungal infections in humans are caused by species of the genera, *Aspergillus*, *Cryptococcus*, and *Candida* [4]. *Cryptococcus neoformans*, ranked among the top fungal pathogens on the World Health Organization's first Fungal Priority Pathogens List, is a globally distributed opportunistic fungus primarily originated from the environment [5]. It poses a significant threat to human health, causing life-threatening cryptococcosis, particularly in immunocompromised individuals [6]. Despite its serious impact on human health, *C. neoformans* infections are often ignored, emphasizing the urgent need to unravel its molecular pathogenesis for the development of new therapeutic options. *Aspergillus fumigatus* is responsible for severe respiratory infections. Invasive pulmonary aspergillosis (IPA) accounts for an estimated 200,000 cases annually [7]. IPA is an aggressive and often fatal disease that primarily affects individuals undergoing chemotherapy, organ transplantation, or suffering from advanced pulmonary diseases [8, 9]. Similarly, *Candida auris* has emerged as a multidrug-resistant yeast responsible for bloodstream infections with high mortality rates. In the United States, clinical cases of *C. auris* surged by 60% in 2020 and doubled in 2021, highlighting its increasing prevalence [10].

The current available antifungal agents, consisting of polyenes, azoles, and echinocandins, are insufficient to manage the mortality caused by fungal infections due to significant off-target effects, the rapid emergence of resistance to antifungal therapeutics, and the emergence of intrinsically drug-resistant fungal pathogens [11]. Therefore, in addition to the development of new formulations of commercially available antifungal drugs, there is an urgent need for the development of alternative classes of broad-spectrum antifungal drugs that are fast-acting and safe.

Autophagy, often referred to as the self-eating machinery, is a fundamental process that maintains intracellular homeostasis by recycling or degrading unnecessary or damaged components, particularly under unfavorable growth conditions [12]. It plays a crucial role in various biological functions, including nutrient sensing, cellular differentiation, tissue homeostasis, aging, immunity, and programmed cell death [13]. More than 30 autophagy-related genes (ATGs) have been identified in yeast, with their homologs conserved across eukaryotes [14, 15]. Among these, the ubiquitin-like protein Autophagy-related protein 8 (ATG8) has a crucial role in autophagosome biogenesis and cargo recruitment. The cysteine protease Autophagy-related protein 4 (ATG4) is required for the maturation and recycling of ATG8, enabling its conjugation to phosphatidylethanolamine (PE) [16]. This modification not only anchors ATG8 to autophagic membranes but also promotes membrane expansion and cargo encapsulation, ensuring the completion of autophagosome formation and the subsequent degradation of their cargoes [16, 17].

Fungal autophagy is responsible for homeostasis in response to nutrient and carbon starvation of which stresses are commonly observed during infection of opportunistic fungal pathogens in hosts [18]. Based on *in vitro* and/or *ex vivo* investigation of autophagy-deficient mutants of fungal pathogens, severe impairments of conidiation and hyphae development have been observed under the stresses while normal development of the autophagy mutants was reported in the replenish condition [18, 19]. *C. neoformans* has been shown to require autophagy process during infection [18-20]. Once *C. neoformans* successfully infects its host, the fungus activates autophagy within host macrophage to cope with unfavorable survival conditions [21]. Studies show that autophagy-defective strains of *C. neoformans* become hypersensitive to nutrient starvation and reactive oxygen species in macrophages [6, 22]. In addition, several *ATG* mutants, including *atg1*Δ, *atg7*Δ, *atg8*Δ, and *atg9*Δ, exhibit avirulent phenotypes [23]. These findings indicate that autophagy in *C. neoformans* plays crucial roles during infection in mammalian hosts. Similarly, in *A. fumigatus*, autophagy regulated by Protein Kinase A (PKA) is essential for fungal survival under nutrient-deprived condition [24] and plays a critical role in pathogenicity [25]. In *C. albicans*, *atg11*Δ results in hypersensitivity to nitrogen starvation and defects in both the cytoplasm-to-vacuole targeting (Cvt) pathway and mitophagy. This mutant also exhibits impaired growth under autophagy-inducing conditions [26]. Although information on the role of autophagy during host-microbe interactions in *Candida* species remains limited, current evidence suggests that fungal autophagy is essential for maintaining cellular homeostasis in response to developmental cues and environmental stress. Together, targeting autophagy represents a promising strategy for antifungal drug discovery.

In previous studies, a bioluminescence resonance energy transfer (BRET) system was employed to monitor the ATG4-mediated cleavage of ATG8 in the format of high-throughput screening (HTS) platforms [27-29]. It has been shown that novel autophagy modulators target ATG4s of *Botrytis cinerea* and *Magnaporthe oryzae*, thereby influencing fungal development and pathogenesis in the hosts [28]. While prior research has primarily focused on plant-pathogenic fungi interaction, this study investigates the antifungal activity of these modulators against human-pathogenic fungi. Our findings reveal that ebselen and its analogs not only target ATG4-mediated ATG8 processing in plant pathogenic fungi but also inhibit the same process in several clinical isolates of *Cryptococcus* and *Aspergillus* species. Furthermore, all tested autophagy inhibitors exhibited antifungal activity against *Cryptococcus* and *Candida* species. These results demonstrate that ebselen and its analogs exert antifungal effects across multiple human pathogenic fungal species, highlighting their potential as novel therapeutic agents for broad-ranged invasive fungal infections. Taken together, evolutionarily conserved autophagy represents an excellent target pathway for the discovery of broad-spectrum antifungal agents against clinically important fungal pathogens.

## Materials and Methods

### Identification of ATG4 and ATG8 in Fungal Genomes

Selected fungal species were defined to search ATG4 and ATG8 in Uniprot [30]. As a group of representative human fungal pathogens listed in the Center for Disease Control and Prevention CDC [5], *Cryptococcus* spp., *Aspergillus* spp., *Candida* spp., *Histoplasma* spp., *Coccidioides* spp., *Mucor* spp., and *Rhizopus* spp., were selected to search ATG8 and ATG4 in their genomes. The initial key word search failed to identify ATG8 and ATG4 in several species. Therefore, the term ‘autophagy’ was used to search relevant sequences, which were then sorted based on key features, such as the conserved glycine residue at the carboxyl terminal (C-terminal) region of ATG8. While ATG8 proteins in fungal species were relatively well annotated, the keyword search with autophagy failed to identify ATG4s in several fungal species. Therefore, cysteine proteases were searched and refined to obtain a final list of potential ATG4s that preserve the enzymatic catalytic triad residues [31]. Final listed proteins were used for further sequence analyses (Data S1 and S2).

### Phylogenetic Analysis and Data Visualization

Multiple sequence alignment (MSA) was performed using Clustal Omega, a highly accurate alignment tool that utilizes a seeded guide tree and HMM profile-profile techniques to generate alignments [32]. The aligned sequences were then subjected to phylogenetic analysis using IQ-TREE (version 2.3.6) [33] on a high-performance computing (HPC) cluster provided by Advanced Research Computing Center (ARCC), University of Wyoming. The MSA was performed using Clustal Omega via the EBI web server, while the tree generation was executed using IQ-TREE with the command iqtree2 -s <alignmentfilename> -m MFP –bb 1000. This command employs Model Finder Plus (-m MFP) to automatically determine the best-fit evolutionary model based on statistical criteria and conducts ultrafast bootstrap resampling (-bb 1000) with 1,000 replicates to evaluate branch support and tree reliability. The final tree, along with its associated bootstrap values, was visualized using iTOL v7, providing an interactive platform for annotation and interpretation [34].

### Protein Modeling and Structure Analysis

Protein structural models of *Cn*Atg4 were resolved by AlphaFold2-multimer integrated in Google Colab [35] or on AlphaFold2.3.2 in a high-performance computing (HPC) cluster provided by ARCC, University of Wyoming [36]. Structure model of a truncated *Cn*Atg4 (*Cn*Atg4T) was compared with ATG4 of *B. cinerea* protein structure that was simulated in our previous study [28] or human ATG4 structure that was retried from a co-crystal structure of human ATG4B and *Hs*LC3 deposited in the protein data bank, RCSB (PDB, 2Z0E [16]) by using a matchmaker function of ChimeraX 1.10 daily [37]. Docking simulations were carried out using DiffDock, an artificial intelligence (AI)-based generative diffusion model for protein-ligand pose prediction [38]. The model generated multiple candidate poses through a reverse diffusion process and automatically ranked by the DiffDock Confidence score. The top 10 ranked poses were selected and aligned within the binding pocket. Docking geometries were visualized and inspected using ChimeraX 1.10 daily.

### Plasmid Construction of ATG4s and ATG8 Synthetic Substrates

*Cn*Atg4T (J9VN57), *Af*Atg4 (A0A9P9N7X1), and *An*Atg4 (A2QY50) open reading frames (ORFs) were synthesized and cloned into pET28a expression vector (Twist Bioscience, USA), resulting in 6xHis-ATG4. For BRET-based substrates of ATG8s, the synthesized ORF fragments of *Cn*Atg8 (P0CO54), *Af*Atg8 (B0XPW3), and *An*Atg8 (G3XZT6), digested with BamHI-HF (New England Biolabs, R3136S, USA) and SalI-HF (New England Biolabs, R3138S), were cloned into the corresponding site of the pET28-Citrine-*Bc*Atg8-ShR plasmid [28], replacing *Bc*Atg8 with *Cn*Atg8, *Af*Atg8, or *An*Atg8. All plasmid sequences were confirmed by whole plasmid sequencing (Plasmidsaurus, USA). Primer sequences used for cloning are available upon request. All plasmids of 6xHis-ATG4s and ATG8 synthetic substrates were transformed into *E. coli* strain Rosetta (DE3) competent cells (Novagen, USA) for recombinant protein purification.

### Protein Expression and Purification

We followed the experimental procedures described in Woo *et al*. [27]. Protein expression in *E. coli* was induced with 1 mM isopropyl β-D-thiogalactopyranoside (IPTG) (GoldBio, USA) at OD_600_ of 0.6-0.8 and the culture was incubated at 18°C overnight. Cells were collected by centrifugation, lysed with equilibrium buffer (50 mM sodium phosphate, 300 mM sodium chloride, 10 mM imidazole; pH 7.4), and sonicated with 10 s on/off pulses at 10%power on ice for 7 m per 500 ml of culture. After centrifugation, soluble fraction was loaded in a column chromatography cartridge packed with HisPur Cobalt Resin (Thermo Fisher Scientific, USA), washed with a washing buffer (50 mM sodium phosphate, 300 mM sodium chloride, 10 mM imidazole; pH 7.4), and then eluted with an elution buffer (50 mM sodium phosphate, 300 mM sodium chloride, 150 mM imidazole; pH 7.4). Affinity-purified recombinant proteins were dialyzed against 1X phosphate-buffered saline (PBS, pH 7.2) at 4°C overnight and the dialyzed proteins were analyzed by sodium dodecyl sulfate-polyacrylamide gel electrophoresis (SDS-PAGE). Approximate protein concentration was determined by comparison to a bovine serum albumin (BSA) standard (Thermo Fisher Scientific) after Coomassie blue staining (Labsafe GEL Blue, G-Bioscience, USA).

For microscale thermophoresis (MST) assay, purified *Cn*Atg4T was concentrated using Amicon^®^ Ultra Centrifugal Filter, 10 kDa MWCO (Millipore, USA). Concentrated *Cn*Atg4T was gel filtrated using a Superdex 200 Increase 10/300 GL on an AKTA pure chromatography system (Cytiva, USA).

### *In Vitro* Cleavage Assay and Western Blot

Chemical application for the *in vitro* cleavage assay was followed the procedure described in Woo and Jung *et al*.[28]. Approximately 100 ng of the purified ATG4s and 10 μM of chemicals (EB, EO, and PT) were incubated at room temperature (RT) for 10 min. 200 ng of the ATG8 synthetic substrates were added and then incubated for an additional 10 min. Total reaction volume was 20 μl. For reversible covalent inhibition assay, 50 mM of dithiothreitol (DTT) was treated for 10 min after chemical treatment. The total proteins were boiled for 5 min at 95°C with 2X Laemmli buffer (Bio-Rad, USA) and separated on Mini-protean TGX precast gels 4–20% Resolving Gel (Bio-Rad) using Tris-glycine SDS buffer at 120 V. After SDS-PAGE, the proteins were transferred to a 0.45 μm polyvinylidene difluoride (PVDF) membrane (Bio-Rad) using the Trans-Blot Turbo Blotting System (Bio-Rad). After transferring the proteins onto a PVDF membrane, immunoblot analyses were performed using antibodies against *Renilla* luciferase (1:5000, MAB4400, Sigma-Aldrich, USA) and anti-mouse IgG-HRP secondary antibody (1:5000, Kindle Bioscience) followed by developing the blot with SuperSignal West Pico PLUS Chemiluminescent Substrate (Thermo Fisher Scientific, USA). To estimate relative enzyme activity, ImageJ (National Institutes of Health, USA) and GraphPad Prism 8 (GraphPad, USA) were used for quantification.

### Bioluminescence Resonance Energy Transfer (BRET) Measurement

As described in Woo *et al*. [27], approximately 100 ng of purified *Cn*Atg4T and 10 μM of each chemical were incubated at RT for 10 min and then 200 ng of the *Cn*Atg8-sensor added in a 96-well plate. 100 μM solution of coelenterazine (CLZ) was prepared by dilution with 100% ethanol and protected with aluminum foil from light because CLZ is light sensitive. BRET measurement using a microplate reader (TECAN, USA) was recorded after automatic injection of 10 μl of 100 μM of CLZ. 460 nm and 540 nm filters were used to measure blue luminescence and yellow fluorescence, respectively.

### Microscale Thermophoresis (MST)

MST assay was performed as described in the experimental protocol [39]. For protein labeling, *Cn*Atg4T was diluted to 45 nM in 1X PBS-Tween 20 (PBS-T) and the RED-tris-NTA 2^nd^ Generation dye (NanoTemper, MO-L018, USA) was diluted in the same buffer to 100 nM. Protein and dye were mixed in a 1:1 volume ratio and incubated for 30 min at RT. Before the MST measurements, every labeled protein was centrifuged at 12,000 g for 10 min at 4°C to remove potential aggregates.

For all thermophoretic measurements, Monolith Capillaries (NanoTemper) were used. In all MST experiments, the final concentration of His-tag-labeled *Cn*Atg4T was set to 45 nM. Serial dilutions of EB at different concentration ranges were prepared in dimethyl sulfoxide (DMSO). Final samples were prepared by mixing equal volumes (20 μl) of 45 nM His-tag labeled *Cn*Atg4T with 2 μl of EB at a specified concentration. Measurements were performed in PBS-T, with EB concentrations typically ranging from 0 to 200 μM. Measurements of *Cn*Atg4T-EB interaction were performed using a NanoTemper Monolith NT.115 instrument. Before MST measurements, samples were equilibrated for 10 min at RT in the dark, loaded into capillaries, and then inserted into the data collection instrument (with the temperature set at 25°C). The final RED-tris-NTA dye concentration of 50 nM yielded the fluorescence intensity of labeled *Cn*Atg4T around 400 counts at a light-emitting diode (LED) power of 40%. The samples were measured at medium MST power with a pre-MST period of 3 sec, a laser-on time of 20 sec, and a laser-off time of 1 sec.

### Minimum Inhibitory Concentration (MIC) Tests Following European Committee on Antimicrobial Susceptibility Testing (EUCAST) and Clinical and Laboratory Standards Institute (CLSI) Guidelines

Wild-type fungal strains were grown overnight at 30°C in YPD medium, washed twice, and resuspended in sterile distilled H_2_O (dH_2_O). For the MIC assays conducted according to EUCAST guidelines [40], the cell suspension was adjusted to an OD_600_ of 0.5. A 200 μl aliquot of the cell suspension was mixed with 10 ml of RPMI medium (pH 7.4, buffered with 0.165 M MOPS, and 2% glucose) and distributed into 96-well plates containing two-fold serial dilutions of the tested compounds. The plates were incubated at 35°C for 2 to 3 days, and cell density in each well was measured at OD_595_ to determine the MIC values. After assessing growth, 3 μl of culture from each well was spotted onto YPD plates (2% peptone, 1% yeast extract, 2% glucose, and 2% agar) and incubated at 30°C for 24 h to evaluate the fungicidal effects of the tested compounds.

For CLSI MIC tests [41], overnight cultures of each strain were grown in 2 ml of YPD medium at 30°C, washed twice, and diluted 100-fold with sterile dH_2_O. The cell density was adjusted to a final concentration of 2.5 × 10^3^ cells/ml. The cell suspensions were mixed with RPMI medium (pH 7.0, buffered with 24.53 g/L MOPS). The tested compounds (EB, EO and PT) were serially diluted to the desired concentrations, and 96-well plates were prepared by dispensing 200 μl per well, containing both the diluted compounds and the cell suspension. The plates were incubated at 35°C for 48 h for *Candida* species and 72 h for *Cryptococcus* species. The MIC value was defined as the lowest drug concentration at which no visible growth was observed.

### Major Reagents and Antibodies Used

Chemicals and reagents: Ebselen (Cayman chemicals, 70530, USA), ebselen oxide (Cayman chemicals, 10012298, USA), and PT (Cayman chemicals, 16272), IPTG (GoldBio, I2481), HisPur Cobalt Resin (Thermo Fisher Scientific, 89965). Antibodies: anti-*Renilla* luciferase (Sigma-Aldrich, MAB4400). The results were analyzed and visualized by using ImageJ (National Institutes of Health) and PRISM 8 (GraphPad).

### Statistical Analysis

Statistical analyses of the results in this study were performed with PRISM 8 statistical and graphical software (GraphPad). IC_50_ calculation was fit to nonlinear regression model using the least square regression method. The R^2^ of EB, EO, and PT treatment was 0.82, 0.91, and 0.98, respectively. The result of MST assay was visualized by a normalized dose-response equation that is preset in PRISM 8. The R2 to evaluate the goodness of fit was 0.8995.

## Results

### Analysis of ATG4s and ATG8s from Major Human Fungal Pathogens

**Fig. 1:**
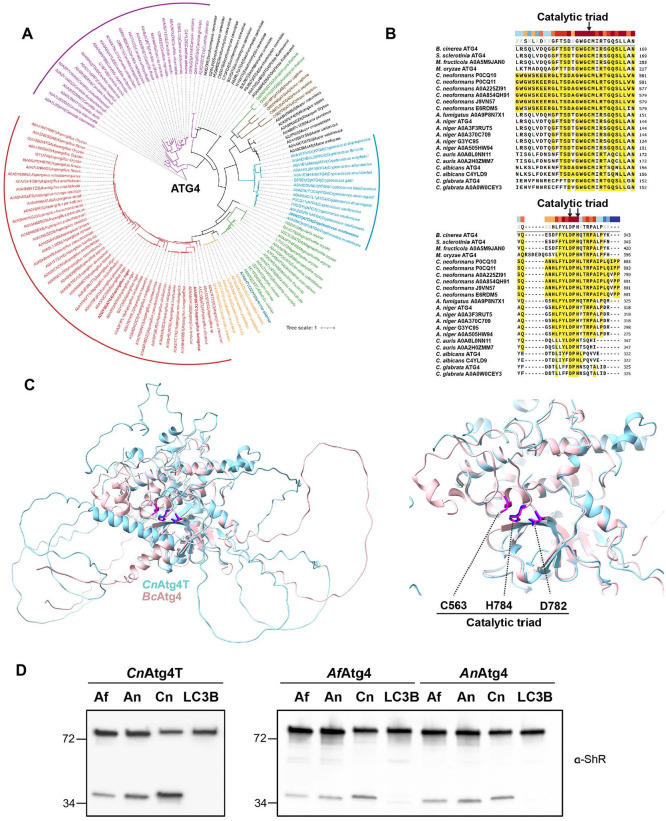
Sequence and structural analyses of ATG4 cysteine proteases of human fungal pathogens. (**A**) The phylogenetic tree of ATG4 presenting clustered ATG4 from species within the same genus. (**B**) Amino acid sequence alignment of the catalytic triad of ATG4 proteins of *Cryptococcus* ssp., *Aspergillus* ssp., and *Candida* ssp., compared to other fungal ATG4s studied in [28]. (**C**) The comparison of the structural model of truncated *Cn*Atg4 with *Bc*Atg4. Structures of core enzymatic activity containing the catalytic triad have well aligned each other, supporting the functionality of the truncated *Cn*Atg4 (*Cn*Atg4T). (**D**) The cleavage of various fungal ATG8 substrates by fungal ATG4s. *Af*Atg4, *An*Atg4, and *Cn*Atg4T could process fungal ATG8s but not *Hs*LC3B. *n* = 3. *Af*, *A. fumigatus*; *An*, *A. niger*; *Cn*, *C. neoformans*; *Hs*, *Homo sapiens*.

Autophagy-related protein 4 (ATG4) is a cysteine protease classified as a family C54 enzyme and plays a critical role in both Autophagy-related protein 8-phosphatidylethanolamine (ATG8-PE) conjugation and its subsequent delipidation, which is essential for autophagosome formation [31]. To investigate the evolutionary conservation of ATG4 and ATG8 across fungal pathogen genomes, phylogenetic analyses were conducted based on their aligned protein sequences (Figs. 1A and S1). As ATG4 was not annotated in several fungal pathogen genomes, all cysteine proteases from major human fungal pathogens were retrieved and manually examined to identify those harboring the catalytic triad, a characteristic hallmark of ATG4 proteins (Data S1 and Fig. 1B). Interestingly, while the amino acid sequence homology of ATG8 in major fungal pathogens is greater than 80%, ATG8 proteins from species within the same genus are grouped and, often identical (Data S2 and Fig. S1).

ATG4 proteins of fungal species in the same genus also cluster together, however, the overall sequence homology of ATG4 proteins in major human fungal pathogens is significantly lower than that of ATG8 (Fig. 1A). Therefore, amino acid sequences of catalytic triads of ATG4 cysteine protease were compared and found that they are conserved in *C. neoformans* ATG4 (*Cn*Atg4) (Fig. 1B). Unlike other fungal ATG4 proteins, *Cn*Atg4 possesses expanded, non-aligned amino acid sequences at its amino terminus (N-terminus) and protein structural models of the full length *Cn*Atg4 showed long disorder region of the N-terminus (Fig. S2A). Therefore, we generated a trimmed version excluding this N-terminus (*Cn*Atg4T) (Fig. S2B) [31]. To explore the functional core unit of *Cn*Atg4 as a protease capable of cleaving *Cn*Atg8, the structural model of *Cn*Atg4T was aligned with *Botrytis cinerea* ATG4 (*Bc*Atg4) whose structure was predicted and inhibitors were identified by the bioluminescence resonance energy transfer (BRET)-based screening successfully in previous study [28]. This alignment revealed conserved catalytic triads, Cys563/Cys171, Asp782/Asp345, and His784/His347, corresponding to the residues of *Cn*Atg4 and *Bc*Atg4, respectively [31]. These catalytic triad residues were positioned within the structurally well-aligned active site of *Cn*Atg4, closely matching those in *Bc*Atg4 (Fig. 1C). Structural predictions further suggest that the expanded N-terminus of *Cn*Atg4 comprises intrinsically disordered regions, while the C-terminus contains all the structurally conserved regions. Although the precise *in vivo* function of the disordered regions remains unknown, the functional core unit of the C-terminal *Cn*Atg4 appears to contribute to the protease’s potential functionality that cleaves ATG8 (Fig. 1C, right). Based on these findings, we constructed a truncated version of *Cn*Atg4, *Cn*Atg4T, which retains the conserved catalytic triad and active site to facilitate the investigation of its potential protease activity toward ATG8.

To assess whether *Cn*Atg4T can function as the catalytic core unit of the ATG4 cysteine protease, we expressed and purified recombinant *Cn*Atg4T using an *Escherichia coli* expression system. Previously, BRET-based synthetic sensors were utilized as ATG8 substrates to investigate the biochemical properties of ATG4s [29, 42]. The molecular basis underlying the BRET-based sensor is detailed below. Our *in vitro* cleavage assay results demonstrated that *Cn*Atg4T, *Aspergillus fumigatus* ATG4 (*Af*Atg4), and *Aspergillus niger* ATG4 (*An*Atg4) efficiently processed the fungal ATG8 sensors but failed to cleave the human ATG8 sensor (*Hs*LC3B sensor) (Fig. 1D). *Candida* species were not included in the biochemical assays to evaluate ATG4-mediated processing of ATG8 because *Candida glabrata* is closely related to *Saccharomyces cerevisiae* within the *Saccharomycetaceae* family [43]. We have previously conducted biochemical assays using *Sc*Atg4 [42]. It is worth noting that biochemical characteristics of *Sc*Atg4 are comparable to those of other fungal ATG4s, as shown in Fig. 1D. These findings are consistent with previous studies and suggest that fungal ATG4 exhibits cross-kingdom substrate recognition of ATG8 except for *Hs*LC3 [42]. The conservation of catalytic triad residues and structural similarity among fungal ATG4s suggest that ATG4-mediated processing of ATG8 is an evolutionarily conserved mechanism (Fig. S2C). This conservation underscores the essential role of ATG4 in fungal autophagy and highlights its potential as a target for intervention against a broad range of eukaryotic pathogens. Interestingly, *Cryptococcus* species encode an unusually large ATG4 with the N-terminal disordered regions, distinguishing it from other fungal ATG4 homologs. To fully understand the role of these N-terminal disordered regions beyond the cysteine protease function, *in vivo* functional studies are required. Nevertheless, *Cn*Atg4T alone is sufficient for canonical ATG4 activity, providing a valuable tool for *in vitro* biochemical analyses and high-throughput screening (HTS) for the *Cn*Atg8 maturation using the BRET-based synthetic sensor of *Cn*Atg8. This enables the exploration of its enzymatic properties and supports the development of new antifungal agents for the treatment of cryptococcosis.

### Evaluation of the Autophagy Inhibitors EB, EO and PT Targeting Fungal ATG4-Mediated Cleavage of ATG8

**Fig. 2:**
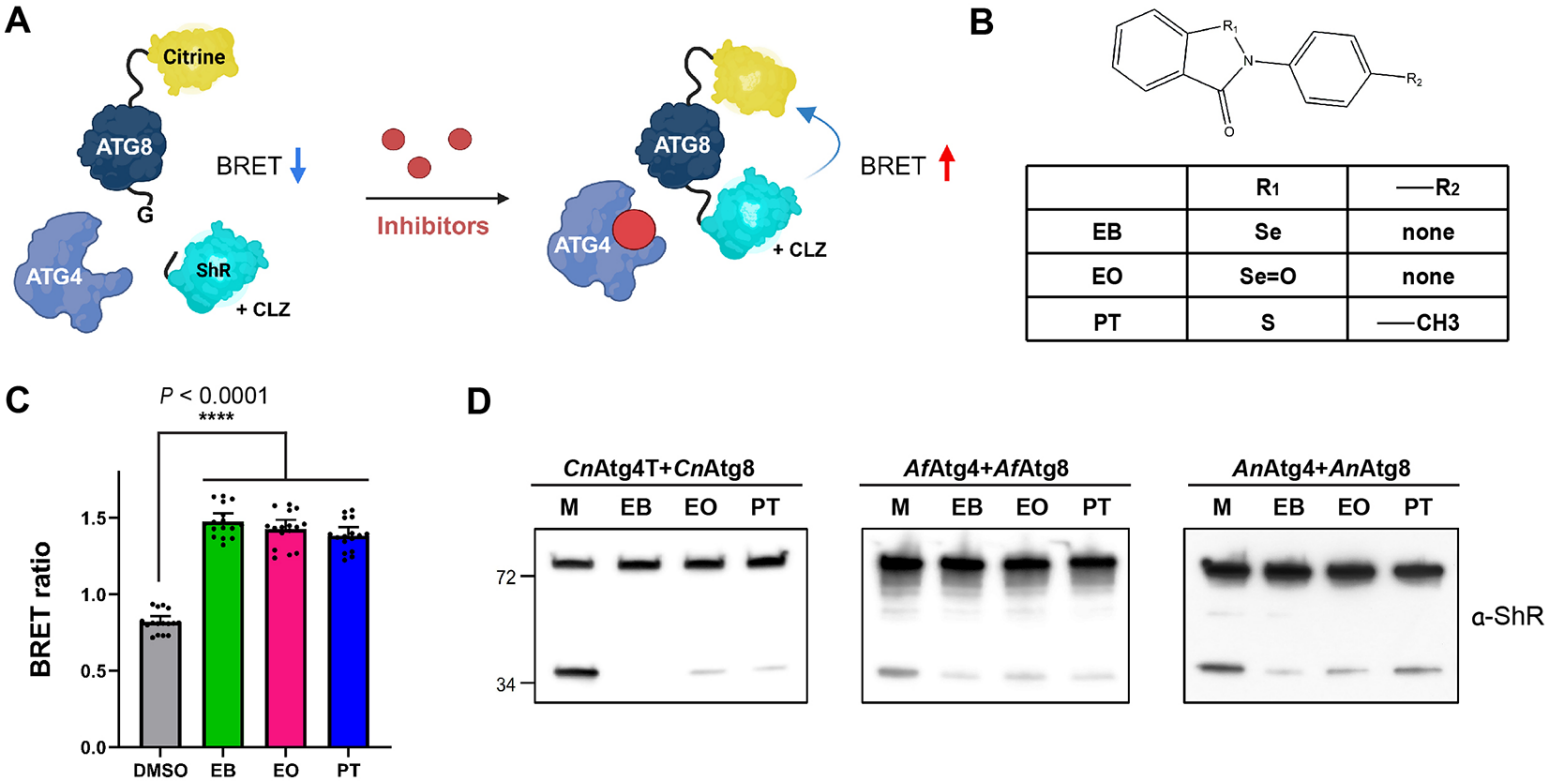
Evaluation of autophagy inhibitors in the maturation of ATG8s in human pathogenic fungi. (**A**) Schematics of high-throughput screening (HTS) using the bioluminescence resonance energy transfer (BRET)-based ATG8 sensor in which the citrine fluorescence protein and a modified *Renilla* luciferase, SuperhRLUC (ShR), are fused to the N- and the C-terminus of ATG8, respectively. CLZ, coelenterazine. (**B**) Chemical scaffolds of EB, EO, and PT. R_1_ and R_2_ represent the functional group and side chain, respectively. The corresponding atoms are shown in the table. (**C**) BRET ratio of the autophagy inhibitor treatments. High BRET ratios upon treatment with autophagy modulators indicate inhibition of *Cn*Atg8 maturation, compared to the DMSO control. The graph presents mean and standard error (SE). *P* < 0.0001 (****), ordinary one-way ANOVA. *n* = 4. (**D**) The *in vitro* cleavage of BRET-sensors by *Cn*Atg4T (left), *Af*Atg8 sensor (middle), and *An*Atg8 sensor (right) confirmed the inhibition of ATG4-mediated ATG8 processing by EB, EO and PT, compared to the DMSO (**M**) control. *n* = 3.

We previously established an optimized HTS platform utilizing a BRET-based synthetic ATG8 sensor to identify autophagy modulators targeting plant fungal pathogens [28]. In brief, for BRET-based synthetic ATG8 sensors, the citrine fluorescent protein (Citrine), serving as the resonance energy acceptor, and an engineered Super human-codon-optimized *Renilla* luciferase (ShR) [44] as the donor, were fused to the N- and C-termini of the *Cn*Atg8, respectively, to construct the *Cn*Atg8 sensor (Fig. 2A). This synthetic sensor is suitable for both *in vitro* and *in vivo* BRET assay [27-29]. In its uncleaved form, the *Cn*Atg8 sensor enables BRET to occur in the presence of the luciferase substrate coelenterazine (CLZ), due to the close proximity between Citrine and ShR. However, cleavage of the *Cn*Atg8 sensor by *Cn*Atg4T separates Citrine and ShR, leading to a significant reduction in the BRET ratio (yellow fluorescence/blue luminescence). Therefore, inhibition of *Cn*Atg4T by chemical modulators leads to an accumulation of the intact *Cn*Atg8 sensor, resulting in an increased BRET ratio. This accumulation of full-length *Cn*Atg8 sensor can be confirmed by an *in vitro* cleavage assay.

Through this screening platform, we reported autophagy modulators as effective fungicidal agents against agriculturally important Ascomycota pathogens [28]. Ebselen (EB), ebselen oxide (EO), and 2-(4-methylphenyl)-1,2-benzisothiazol-3(2H)-one (PT) (Fig. 2B) was identified as inhibitors of ATG8 maturation via ATG4 inhibition in *B. cinerea* and *Magnaporthe oryzae* [28]. Given the evidence of cross-reactivity and structural similarities including conserved catalytic triads and active sites among ATG4s, as well as the finding that *Cn*Atg4T functions as the catalytic unit responsible for ATG4 protease activity (Figs. 1D and S2C), we further performed AI-based docking simulation using DiffDock [38]. The simulation of *Cn*Atg4T with EB revealed a similar binding position of EB within the catalytic pocket of the *Cn*Atg4T enzyme (Fig. S2D), consistent with the docking simulation obtained for *Bc*Atg4 (Fig. S12 in [28]). Based on these biochemical and *in silico* analyses, we hypothesized that the autophagy modulators previously characterized against *Bc*Atg4 [28] may also inhibit the autophagy core unit in human fungal pathogens across different genera, resulting in impeding the proteolytic cleavage of fungal ATG8s.

To assess the inhibitory effects of the autophagy modulators on *Cn*Atg4T-mediated cleavage of *Cn*Atg8, we monitored BRET ratios using the *Cn*Atg8 sensor and recombinant *Cn*Atg4T in the presence of each autophagy inhibitor (Fig. 2C). In parallel, we conducted *in vitro* cleavage assays using *Cn*Atg8, *Af*Atg8, and *An*Atg8 substrates alongside their respective recombinant proteases, *Cn*Atg4T, *Af*Atg4, and *An*Atg4 (Fig. 2D). The results demonstrated that EB, EO, and PT significantly inhibited the cleavage activity of *Cn*Atg4T toward *Cn*Atg8, indicating effective suppression of ATG4 enzymatic function by these compounds. Additionally, the inhibitors exhibited comparable inhibitory effects on the ATG8 maturation catalyzed by *Af*Atg4 and *An*Atg4 (Fig. 2D).

Collectively, these findings support that EB, EO, and PT act as potent inhibitors of ATG4-mediated ATG8 maturation, suggesting their potential as broad-spectrum autophagy inhibitors in pathogenic fungi. Taken together, these findings highlight the evolutionary conservation of ATG4-mediated processing of ATG8 and reinforce the concept that targeting ATG8 maturation represents a versatile and broadly applicable strategy for the development of effective broad-spectrum autophagy inhibitors against diverse fungal pathogens.

**Fig. 3:**
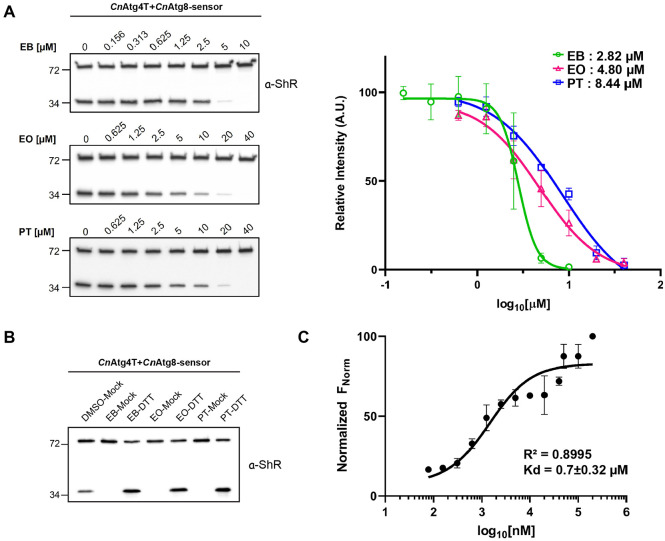
Biochemical properties of autophagy modulators for *Cn*Atg4T-mediated cleavage of *Cn*Atg8. (**A**) IC_50_ values of EB, EO, and PT for the *Cn*Atg8 cleavage are estimated by *in vitro* cleavage assay (left panels). The graph on the right shows the mean and SE. *n* = 3. (**B**) The inhibitory effect of lead compounds (EB, EO, and PT) on the *Cn*Atg4T-mediated maturation of *Cn*Atg8 is fully reversed under reducing conditions. *Cn*Atg4T was pre-treated with 10 μM of each compound, followed by the addition of 50 mM DTT. Two independent experiments were performed with same results. (**C**) The direct binding of fluorescently labeled *Cn*Atg4T to EB was analyzed by Microscale thermophoresis. EB was titrated from 78 nM to 200 μM. The change in the thermophoretic signal yielded a dissociation constant (Kd) of 0.7 ± 0.32 μM. The error bars represent the standard deviation (SD) of each data point calculated from three independent thermophoresis measurements.

Since individual chemical inhibitors showed different inhibitory kinetics on the tested ATG4s (Fig. 2D), we compared their inhibitory potency using the *Cn*Atg4T-mediated cleavage of *Cn*Atg8. Based on the half-maximal inhibitory concentration (IC_50_) values, EB is a stronger inhibitor than EO and PT in the *Cn*Atg8 maturation (Fig. 3A). It has been suggested that the selenium atom in EB serves as the functional group because the primary distinction from non-inhibitory analog scaffolds lies in the modification or substitution of the selenium [28]. Given that ATG4 is a cysteine protease, the selenium atoms in EB and EO, as well as the sulfur atom in PT, are thought to facilitate the formation of selenium-sulfur [45] and disulfide bonds [46] with cysteine residues, respectively. These covalent interactions appear to contribute to the modulation of ATG4 activity. It has been proposed that EB interacts with ATG4 via a covalent modification of the catalytic cysteine, which is consistent with the established reactivity profile of selenium-containing compounds [28].

Previous studies have demonstrated that treatment with the reducing agent dithiothreitol (DTT) disrupts the EB-ATG4 complex and restores enzymatic activity [28], supporting the formation of a reversible covalent bond between the catalytic cysteine of *Cn*Atg4T and EB. To experimentally validate this reversibility in *Cn*Atg4T-mediated cleavage of *Cn*Atg8, we performed *in vitro* cleavage assays under reducing conditions. We observed that the inhibitory effects of the autophagy inhibitors on *Cn*Atg8 maturation were reversed upon DTT treatment (Fig. 3B), suggesting that the Se-S bonds formed by EB and EO, as well as the S-S bond formed by PT with the cysteine residues, are reversible under these conditions. Although equilibrium dissociation constants (Kd) are generally inapplicable to covalent enzyme inhibition owing to the irreversible nature of most covalent interactions, Kd remains applicable for evaluating enzymatic characteristics in cases of reversible covalent modifications [47, 48]. To further characterize the interaction between EB and *Cn*Atg4T, we employed microscale thermophoresis (MST) assays, which revealed a direct binding with a dissociation constant (Kd) of 0.7 ± 0.32 μM (Fig. 3C). These data demonstrate that EB directly binds to *Cn*Atg4T and inhibits *Cn*Atg4T-mediated cleavage of *Cn*Atg8. Notably, this inhibitory effect is also observed in *A. fumigatus* and *A. niger* (Fig. 2D). These findings indicate that, in addition to their efficacy against previously studied Ascomycota [28], EB, EO, and PT also target autophagy biogenesis of *C. neoformans* in Basidiomycota, representing a phylogenetically distinct fungal clade. Taken together, these autophagy inhibitors have the potential to serve as broad-spectrum antifungal agents across various fungal taxa.

### EB Exhibited Antifungal Activity against a Range of Pathogenic Fungi, Showing Greater Efficacy Than Its Derivatives

**Fig. 4:**
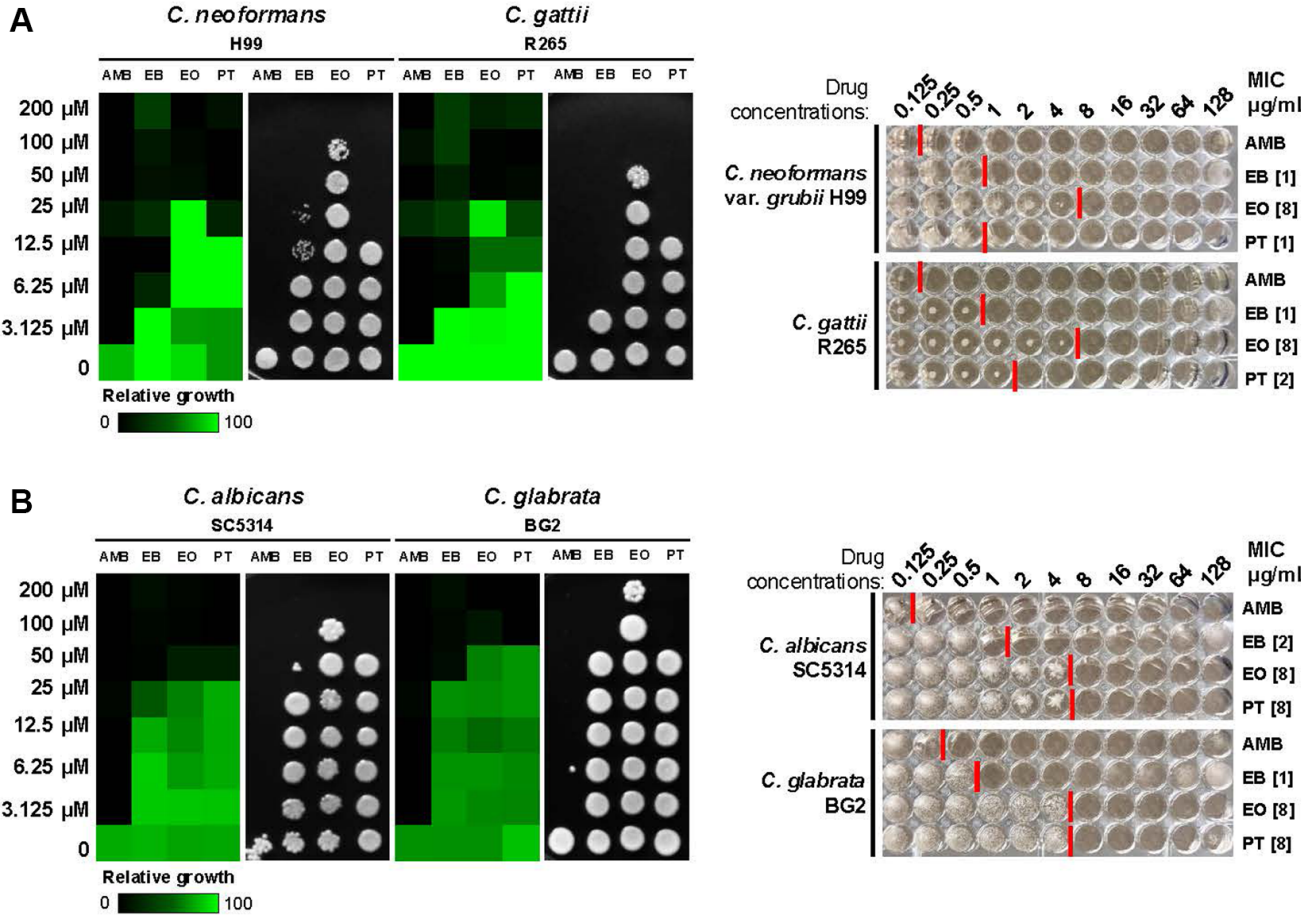
Autophagy inhibitors exhibit antifungal effects against *Cryptococcus* and *Candida* species. (**A**) The EUCAST MIC and CLSI MIC tests for EB, EO, and PT are shown for *C. neoformans* H99 and *C. gattii* R265. (**B**) EB, EO, and PT antifungal effects against *C. albicans* SC5314 and *C. glabrata* BG2 were determined by the same assays shown in (**A**) Amphotericin B (AMB), a broad-spectrum antifungal agent, was included as a control to validate the MIC assay results. After growth assessment, the cultures were transferred onto YPD plates and incubated at 30°C for 24 h to evaluate the fungicidal effects of the tested compounds. Numbers in brackets indicate MIC values.

**Fig. 5:**
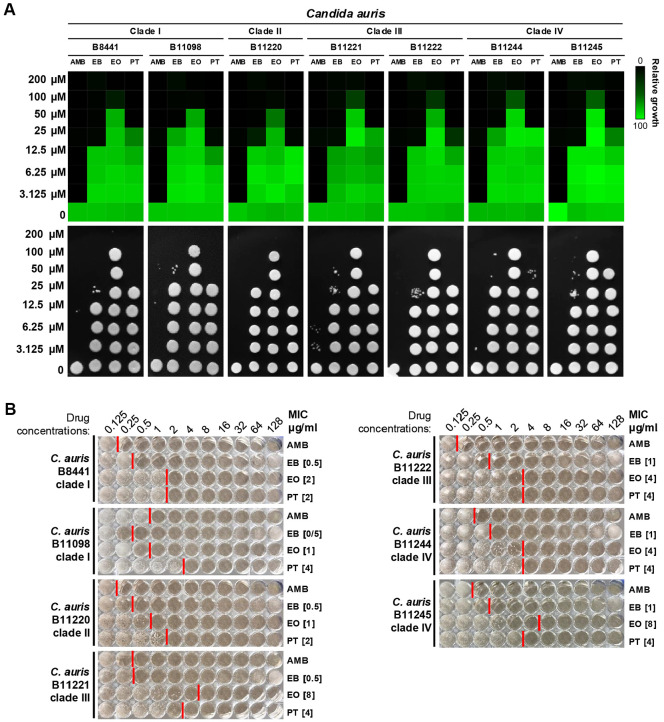
Autophagy inhibitors exhibit antifungal effects against multi-drug resistant *Candida auris*. (**A**) The susceptibility of seven different wild-type strains of *C. auris* to EB, EO, and PT were determined by the EUCAST MIC assay. (**B**) CLSI MIC assays were repeated to determine antifungal efficacy of EB, EO, and PT against *C. auris*. Representatives of four clades of *C. auris* (clade I: B8441 and B11098; clade II: B11220; clade III: B11221 and B11222; clade IV: B11244 and B11245) were used. Amphotericin B (AMB), a broad-spectrum antifungal agent, was included as a control to validate the MIC assay results. Numbers in brackets indicate MIC values.

To investigate the antifungal and fungicidal activities of EB and its analogs against human pathogenic fungi, we conducted susceptibility assays on clinically relevant fungal pathogens. *C. neoformans* and *C. gattii* were selected as representative species of pathogenic *Cryptococcus* species. Additionally, due to the increasing prevalence of antifungal-resistance and the urgent public health threat posed by *Candida* species, we also examined their susceptibility by testing *C. albicans*, *C. glabrata*, and *C. auris*. Antifungal susceptibility test was performed following two standardized minimum inhibitory concentration (MIC) determination methods: the European committee on antimicrobial susceptibility testing (EUCAST) and clinical and laboratory standards institute (CLSI) protocols ([40, 41] ; Figs. 4 and 5). Amphotericin B (AMB), a well-established broad-spectrum antifungal agent commonly used to treat systemic fungal infections, was included as a positive control to compare the antifungal efficacy of the autophagy inhibitors [49]. Our results showed that EB, EO, and PT exhibited antifungal activity against all tested fungal strains with variations in potency (Figs. 4 and 5). Since antifungal drug susceptibility varies among *Candida* species (Fig. 4B), we further evaluated seven wild-type *C. auris* strains representing four distinct clades (clade I to IV) [50] to assess potential clade-specific differences in drug response (Fig. 5). The results indicate that EB, EO, and PT inhibit the growth of *C. auris* isolates across all clades. *In vitro* ATG4 inhibition assay yielded IC_50_ values of 2.28, 4.80, and 8.44 μM for EB, EO, and PT, respectively (Fig. 3A). However, the order of inhibition strength did not always correlate with MIC values (Fig. 4A), likely due to differences in compound stability under assay-specific conditions (*e.g.*, pH and buffer components) as well as variation in membrane permeability, influx activity, or intracellular target accessibility. The distinct functional group and side chain of PT may further influence its physicochemical properties and metabolic stability in fungal cells [51]. Nevertheless, EB exhibited the greatest fungicidal potency with significantly lower MIC values than EO and PT (Figs. 4 and 5). Taken together, autophagy modulators have potential as a lead compound for antifungal drug development against multidrug-resistant *C. auris*.

## Discussion

Autophagy plays a pivotal role in fungal survival and virulence by enabling fungi to adapt and thrive in the hostile environment of the human host during infection [52, 53]. Of particular interest, autophagy-deficient mutants exhibit a complete loss of virulence, underscoring its critical role in fungal pathogenicity [18, 23, 53]. Targeting autophagy modulators, particularly ATG4 inhibitors, represents a promising strategy for antifungal drug development, as EB exhibits fungicidal activity against *Cryptococcus* and *Candida* species, including *C. auris* (Figs. 4 and 5). EB is an organoselenium compound that has already been reported to exert fungicidal effects against various fungal species [54]. However, its mechanism of action and biological targets had not been clearly elucidated. Our study reinforces fungal ATG4 as an effective antifungal target and highlights autophagy as a novel pathway for therapeutic intervention.

However, due to its evolutionary conservation, a fundamental question arises in antifungal drug development: Can autophagy be selectively inhibited in human-pathogenic fungi to reduce their virulence without disrupting autophagy in the human host? In mammalian host-fungal pathogen interactions, phagocytes serve as the first line of defense against invading fungi by engaging both canonical and noncanonical autophagy pathways to eliminate pathogens. Host autophagy can be broadly classified into three major subtypes: canonical autophagy, xenophagy, and LC3-associated phagocytosis (LAP) [55-57]. All subtypes require precise processing of LC3/ATG8 by the cysteine protease ATG4, which exposes the conserved glycine residue necessary for conjugation with phospha-tidylethanolamine (PE). LAP is a non-canonical autophagy pathway crucial for degrading engulfed fungal spores within host cells. Unlike canonical autophagy, LAP is initiated upon the phagocytic uptake of pathogenic fungi such as *Aspergillus* conidia by immune cells such as macrophages and neutrophils. During this process, LC3 conjugates to PE on the phagosome membrane, leading to the formation of the LAPosomes (LC3-decorated phagosomes). This specialized compartment subsequently fuses with lysosomes, enabling the degradation of internalized fungal pathogens and effectively eliminating the threat. LAP plays a pivotal role in host resistance against *A. fumigatus* infections [25, 58-62], and defects in this pathway impair host immune function, thereby increasing susceptibility to fungal diseases [61].

While inhibiting fungal autophagy could impair survival and pathogenicity, broad-spectrum inhibitors that affect both fungal and human autophagy may pose a risk of host toxicity, given the critical roles of autophagy in various human immune cells. The key to unlocking the therapeutic potential of autophagy inhibitors lies in identifying fungi-specific modulators that selectively disrupt fungal autophagy without compromising the host process. Achieving this selectivity would represent a significant breakthrough, enabling the development of safer and more targeted antifungal treatments. Promisingly, although the fungal ATG4 and ATG8 proteins targeted to identify autophagy inhibitors are highly similar to human *Hs*ATG4 and *Hs*LC3, distinct clusters clearly separate the fungal proteins from their human counterparts (Figs. 1A and S1A). Structure comparison of *Cn*Atg4 and human ATG4 showed significant structural differences supporting the potential of screening of fungal autophagy-specific inhibitors (Fig. S3). Although the catalytic triads of these ATG4 proteins are well-conserved (magenta for *Cn*Atg4T and navy for *Hs*ATG4 in Fig. S3), *Cn*Atg4 exhibits several unique structural regions distinct from *Hs*ATG4. Among them, two regions appear particularly promising: an alpha helix connected to the helix containing the catalytic cysteine (top circled region in Fig. S3) and a long helix located near the active site (bottom circled region in Fig. S3). Modification of the upper helical structure may alter the positioning of the catalytic cysteine residue affecting the enzymatic activity. More interestingly, the long helix positioned near the active site could sterically hinder *Cn*Atg8 access to the active site of *Cn*Atg4T upon novel chemical modification, suggesting a potential mechanism for potent inhibition of the fungal autophagy process. The difference between fungal ATG4s and *Hs*ATG4s facilitates the identification of species-specific autophagy modulators that specifically target pathogenic fungi.

In certain fungal infectious disease contexts, precise regulation of autophagy in both pathogens and hosts is essential, as host autophagy can both restrict infection through xenophagy and LAP and paradoxically support fungal persistence. For instance, during early *C. neoformans* invasion of macrophages, host autophagy enhances phagocytosis, yet the fungus replicates within LC3-associated *C. neoformans*-containing vesicles (*Cn*CVs). Inhibition of autophagy with 3-methyladenine (3-MA) reduces both uptake and intracellular replication, indicating that host autophagy can be subverted to create an intracellular niche for fungal proliferation [63, 64]. Therefore, the ability to differentially modulate autophagy in pathogens and hosts during pathogenesis is critical for achieving optimal therapeutic outcomes. Our HTS platform can be customized for species-specific evaluation by substituting ATG8 homologs from various pathogenic fungi and hosts, enabling comparative assessment of ATG4-mediated ATG8 processing across species. This approach facilitates the discovery of selective autophagy inhibitors that attenuate fungal virulence without interfering with host immune-related autophagy, while also allowing precise modulation of host autophagy to prevent its subversion by fungi. Therefore, this strategy offers a safer and more refined antifungal therapeutic avenue.

## Supplemental Materials

Supplementary data for this paper are available on-line only at http://jmb.or.kr.
