# Supplementary material for: Evaluating the Antifungal Potential of Autophagy-Related Protein 4 (ATG4) Inhibitors against Human Fungal Pathogens
Source: J Microbiol Biotechnol. 2025 Dec 15;35:e2509002. doi: 10.4014/jmb.2509.09002 (PMC12723477; doi:10.4014/jmb.2509.09002)
Supplement: Supplementary file 1 [file jmb-35-e2509002-supple.pdf]

Figure S1

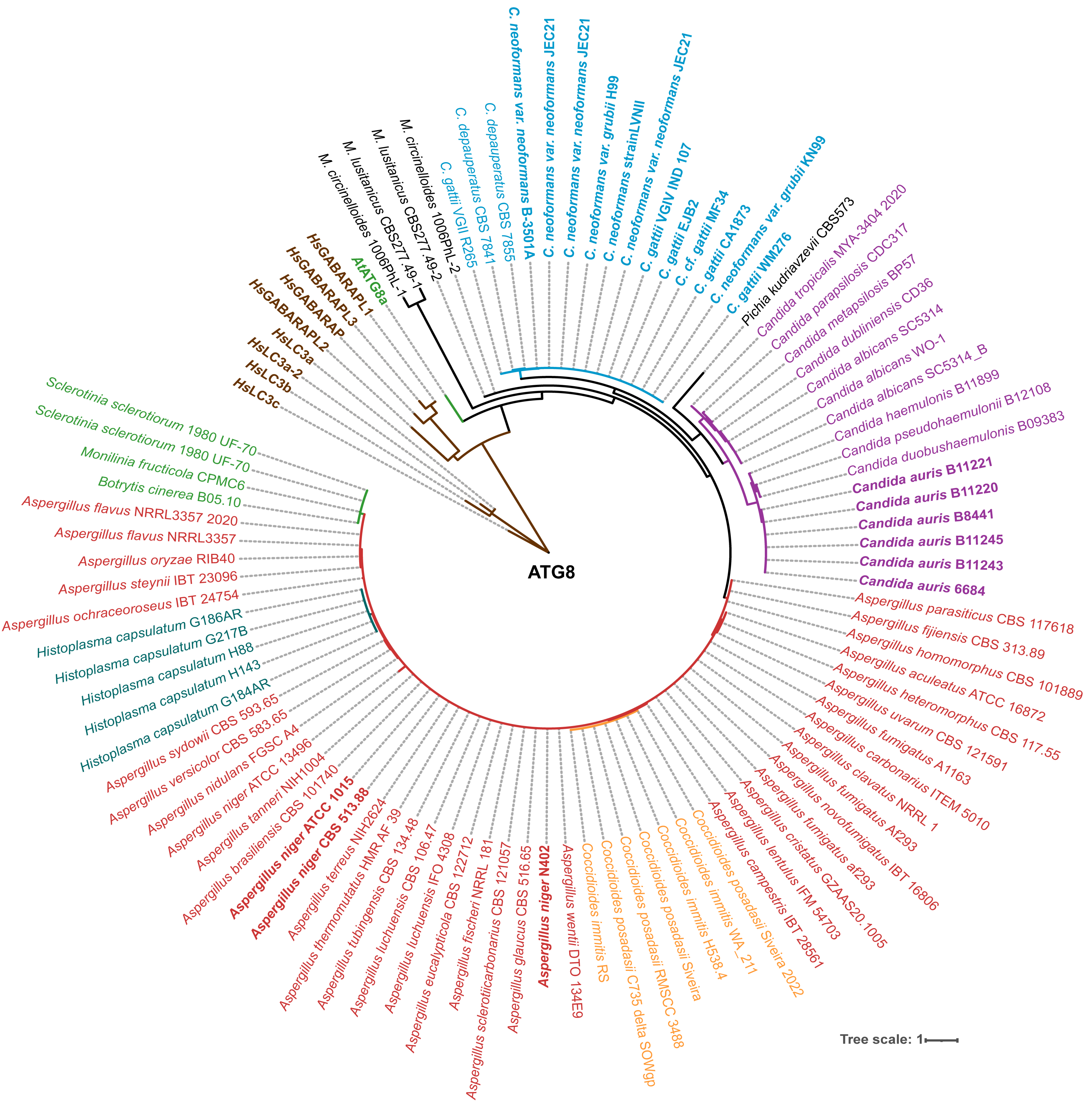
ATG8
Tree scale: 1
C. neoformans var. neoformans B-3501A
C. neoformans var. neoformans JEC21
C. neoformans var. neoformans JEC21
C. neoformans var. grubii H99
C. neoformans strainLVNIII
C. neoformans var. neoformans JEC21
C. gattii VGIV IND 107
C. gattii EJB2
C. cf. gattii MF34
C. gattii CA1873
C. neoformans var. grubii KN99
C. gattii WM276
C. depauperatus CBS 7855
C. depauperatus CBS 7841
C. gattii VGII R265
M. circinelloides 1006PhL-2
M. lusitanicus CBS277.49-2
M. lusitanicus CBS277.49-1
M. circinelloides 1006PhL-1
AtATG8a
HsGABARAPL1
HsGABARAPL3
HsGABARAP
HsGABARAPL2
HsLC3a
HsLC3a-2
HsLC3b
HsLC3c
Pichia kudriavzevii CBS573
Candida tropicalis MYA-3404 2020
Candida parapsilosis CDC317
Candida metapsilosis BP57
Candida dubliniensis CD36
Candida albicans SC5314
Candida albicans WO-1
Candida albicans SC5314_B
Candida haemulonis B11899
Candida pseudohaemulonii B12108
Candida duobushaemulonis B09383
Candida auris B11221
Candida auris B11220
Candida auris B8441
Candida auris B11245
Candida auris B11243
Candida auris 6684
Aspergillus parasiticus CBS 117618
Aspergillus fijiensis CBS 313.89
Aspergillus homomorphus CBS 101889
Aspergillus aculeatus ATCC 16872
Aspergillus heteromorphus CBS 117.55
Aspergillus uvarum CBS 121591
Aspergillus fumigatus A1163
Aspergillus carbonarius ITEM 5010
Aspergillus clavatus NRRL 1
Aspergillus fumigatus Af293
Aspergillus novofumigatus IBT 16806
Aspergillus fumigatus af293
Aspergillus cristatus GZAAS20.1005
Aspergillus lentulus IFM 54703
Aspergillus campestris IBT 28561
Coccidioides posadasii Silveira 2022
Coccidioides immitis WA_211
Coccidioides immitis H538.4
Coccidioides posadasii Silveira
Coccidioides posadasii RMSCC 3488
Coccidioides posadasii C735 delta SOWgp
Coccidioides immitis RS
Aspergillus wentii DTO 134E9
Aspergillus niger N402
Aspergillus glaucus CBS 516.65
Aspergillus sclerotiicarbonarius CBS 121057
Aspergillus fischeri NRRL 181
Aspergillus eucalypticola CBS 122712
Aspergillus luchuensis IFO 4308
Aspergillus luchuensis CBS 106.47
Aspergillus tubingensis CBS 134.48
Aspergillus thermomutatus HMR AF 39
Aspergillus terreus NIH2624
Aspergillus niger CBS 513.88
Aspergillus niger ATCC 1015
Aspergillus brasiliensis CBS 101740
Aspergillus tanneri NIH1004
Aspergillus niger ATCC 13496
Aspergillus nidulans FGSC A4
Aspergillus versicolor CBS 583.65
Aspergillus sydowii CBS 593.65
Histoplasma capsulatum G184AR
Histoplasma capsulatum H143
Histoplasma capsulatum H88
Histoplasma capsulatum G217B
Histoplasma capsulatum G186AR
Aspergillus ochraceoroseus IBT 24754
Aspergillus steynii IBT 23096
Aspergillus oryzae RIB40
Aspergillus flavus NRRL3357
Aspergillus flavus NRRL3357 2020
Botrytis cinerea B05.10
Monilinia fructicola CPMC6
Sclerotinia sclerotiorum 1980 UF-70
Sclerotinia sclerotiorum 1980 UF-70

Figure S2-revised

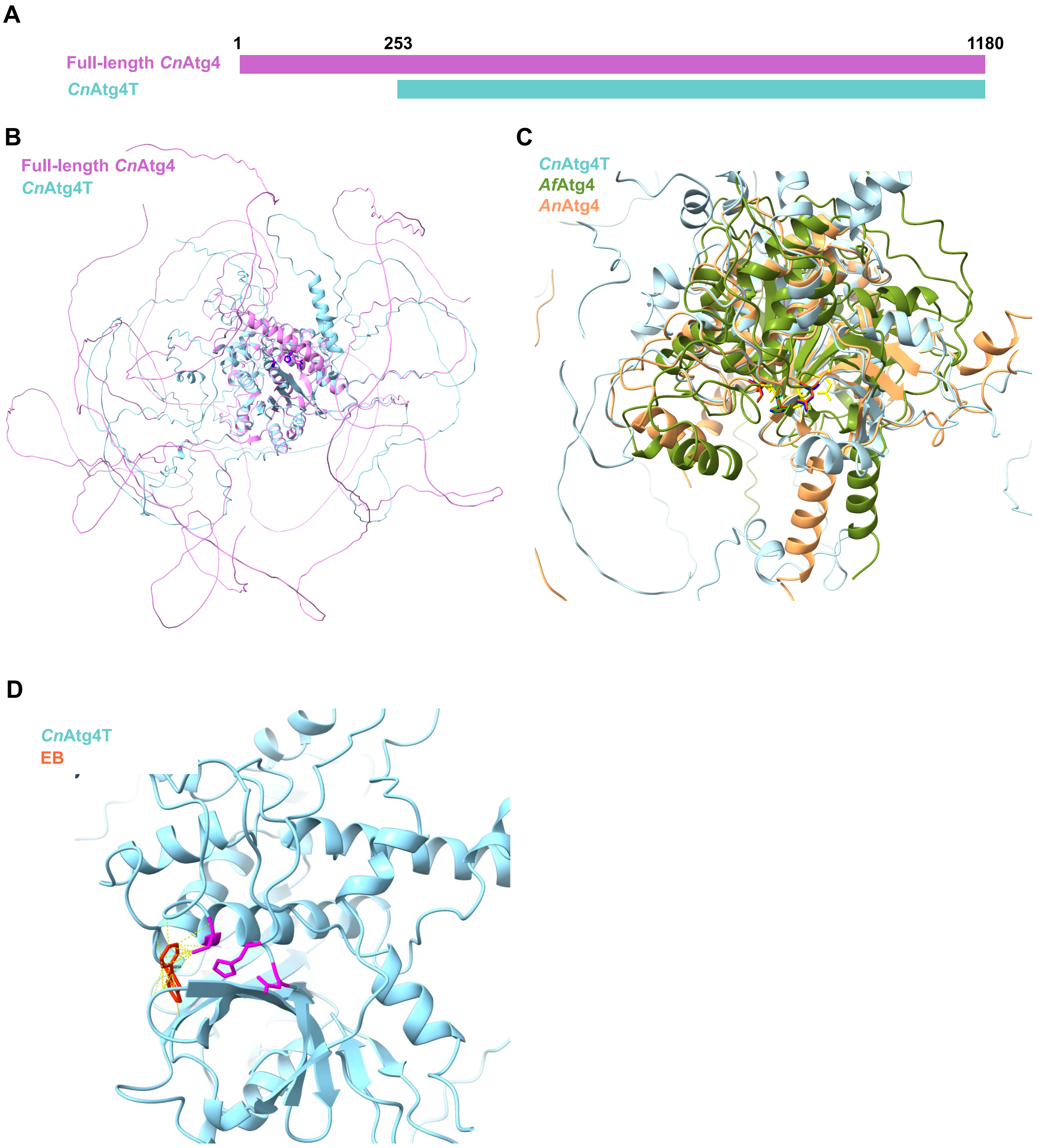

Figure S3 Revised

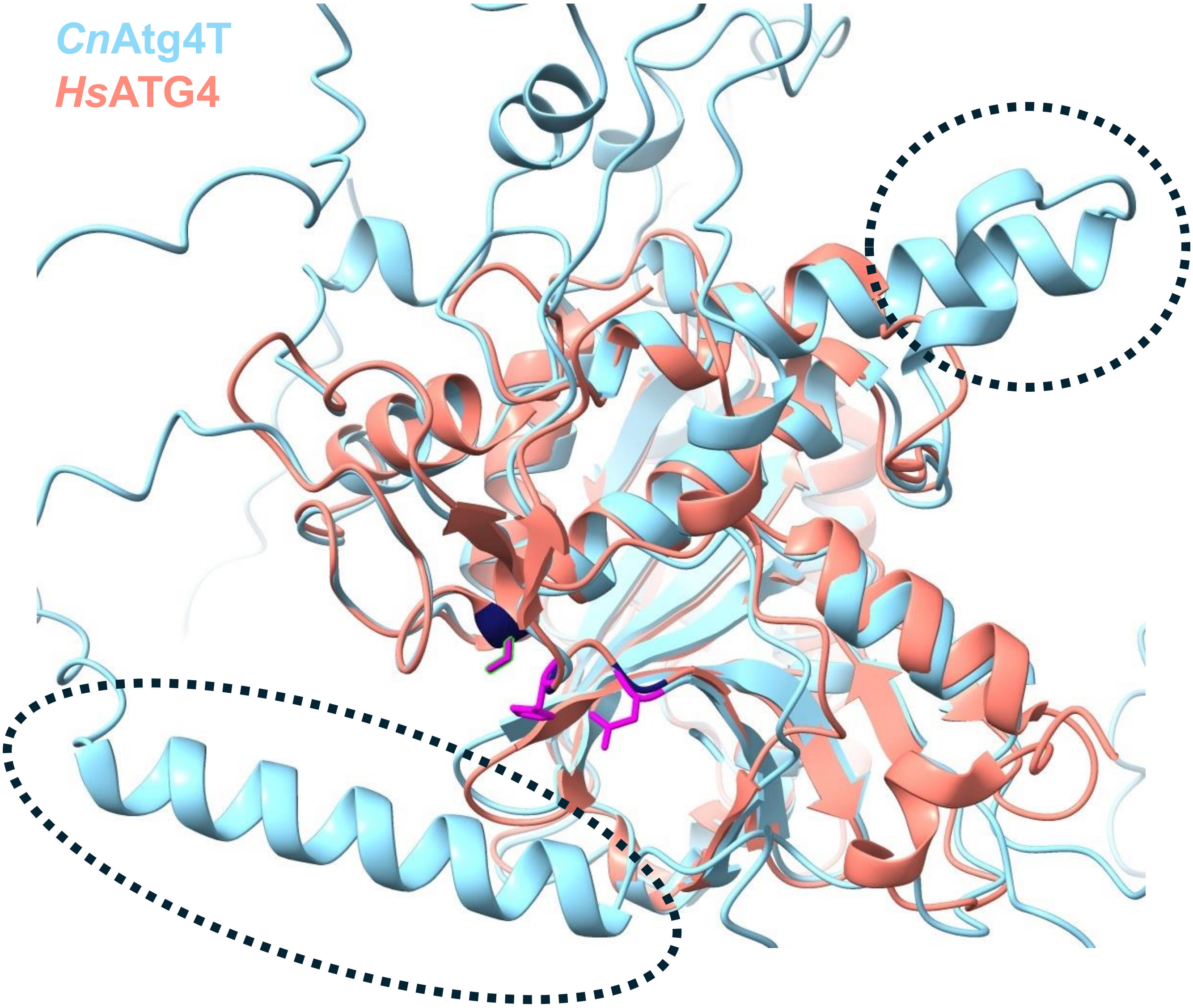

Supplemental Data 1. Sequences of ATG4 proteins of major human fungal pathogens used for Figure 1A.

Highlighted sequences are conserved sequences with Cysteine (Yellow), Aspartic acid (Green), and Histidine (Green) of the catalytic triad.

Green highlighted sequences are

Magenta highlighted sequences in A0A9R1CPD5 of *Cryptococcus cf. gattii* indicates mutation of cysteine of the catalytic triad.

>sp|Q9M1Y0|ATG4B_ARATH Cysteine protease ATG4b OS=Arabidopsis thaliana OX=3702 GN=ATG4B PE=1 SV=1

MKAICDRFVPSKCSSSSTSEKRDISSPTSLVSDSASSDNKSNLTLCSDVVASSSPVSQLCREASTSG
HNPVCTTHSSWTVILKTASMASGAIRRFQDRVLGPSRTGISSSTSEIWLLGVCYKISEGESSEEADAG
RVLAAFRQDFSSLILMTYRRGFEPIGDTTYTSDVNWGCMLRSGQMLFAQALLFQRLGRSWRKKDS
EPADEKYLEILELFGDTEASAFSIHNLILAGESYGLAAGSWVGPYAVCRSWESLARKNKEETDDKHK
SFSMAVHIVSGSEDGERGGAPILCIEDVTKTCLEFSEGETEWPPILLLVPLVLGLDRVNPRYIPSLIATF
TFPQSLGILGGKPGASTYIVGVQEDKGFYLDPHDVQQVVTVKKENQDVDTSSYHCNTLRYVPLESL
DPSLALGFYCQHKDDFDDFCIRATKLAGDSNGAPLFTVTQSHRRNDCGIAETSSSTETSTEISGEEH
EDDWQLL

>sp|Q8S929|ATG4A_ARATH Cysteine protease ATG4a OS=Arabidopsis thaliana OX=3702 GN=ATG4A PE=2 SV=1

MKALCDRFVPQQCSSSSKSDTHDKSPLVSDSGPSDNKSKFTLWSNVFTSSSSVSQPYRESSTSGH
KQVCTTRNGWTAFVKRVSMASGAIRRFQERVLGPNRTGLPSTTSDVWLLGVCYKISADENSGETDT
GTVLAALQLDFSSKILMTYRKGFEPFRDTTYTSDVNWGCMIRSSQMLFAQALLFHRLGRAWTKKSE
LPEQEYLETLEPFGDSEPSAFSIHNLIIAGASYGLAAGSWVGPYAICRAWESLACKKRKQTDSKNQT
LPMAVHIVSGSEDGERGGAPILCIEDATKSCLEFSKGQSEWTPIILLVPLVLGLDSVNPRYIPSLVATFT
FPQSVGILGGKPGASTYIVGVQEDKGFYLDPHEVQQVVTVNKETPDVDTSSYHCNVLRYVPLESLD
PSLALGFYCRDKDDFDDFCLRALKLAEESNGAPLFTVTQTHTAINQSNYGFADDDSEDEREDDWQ
ML

>sp|Q8WYN0|ATG4A_HUMAN Cysteine protease ATG4A OS=Homo sapiens OX=9606 GN=ATG4A PE=1 SV=1

MESVLSKYEDQITIFTDYLEEYPDTDELVWILGKQHLLKTEKSKLLSDISARLWFTYRRKFSPIGGTGP
SSDAGWGCMLRCGQMMLAQALICRHLGRDWSWEKQKEQPKEYQRILQCFLDRKDCCYSIHQM
AQMGVGEGKSIGEWFGPNTVAQVLKKLALFDEWNSLAVYVSMDNTVVIEDIKKMCRVLPLSADTA
GDRPPDSLTASNQSKGTSAYCSAWKPLLLIVPLRLGINQINPVYVDAFKECFKMPQSLGALGGKPN
NAYYFIGFLGDELIFLDPHTTQTFVDTEENGTVNDQTFHCLQSPQRMNILNLDPSVALGFFCKEEKD
FDNWCSLVQKEILKENLRMFELVQKHPSHWPPFVPPAKPEVTTTGAEFIDSTEQLEEFDLEEDFEIL
SV

>sp|Q9Y4P1|ATG4B_HUMAN Cysteine protease ATG4B OS=Homo sapiens OX=9606 GN=ATG4B PE=1 SV=2

MDAATLTYDTLRFAEFEDFPETSEPVWILGRKYSIFTEKDEILSDVASRLWFTYRKNFPAIGGTGPTSD
TGWGCMLRCGQMIFAQALVCRHLGRDWRWTQRKRQPDSYFSVLNAFIDRKDSYYSIHQIAQMG
VGEGKSIGQWYGPNTVAQVLKKLAVFDTWSSLAVHIAMDNTVVMEEIRRLCRTSVPCAGATAFPAD
SDRHCNGFPAGAEVTNRPSPWRPLVLLIPLRLGLTDINEAYVETLKHCFMMPQSLGVIGGKPNSAH
YFIGYVGEELIYLDPHTTQPAVEPTDGCFIPDESFHCQHPPCRMSIAELDPSIAVGFFCKTEDDFND
WCQQVKKLSLLGGALPMFELVELQPSHLACPDVLNLSLDSSDVERLERFFDSEDEDFEILSL

>sp|Q96DT6|ATG4C_HUMAN Cysteine protease ATG4C OS=Homo sapiens OX=9606 GN=ATG4C PE=1 SV=1

MEATGTDEVDKLKTKFISAWNNMKYSWVLKTKTYFSRNSPVLLLGKCYHFKYEDEDKTLPAESGCTI
EDHVIAGNVEEFRKDFISRIWLTYREEFPQIEGSALTTDCGWGCTLRTGQMLLAQGLILHFLGRAWT
WPDALNIENSDSESWTSHTVKKFTASFEASLSGEREFKTPTISLKETIGKYSDDHEMRNEVYHRKIIS
WFGDSPLALFGLHQLIEYGKKSGKKAGDWYGPAVVAHILRKAVEEARHPDLQGITIYVAQDCTVYN
SDVIDKQSASMTSDNADDKAVIILVPVRLGGERTNTDYLEFVKGILSLEYCVGIIGGKPKQSYYFAGF
QDDSLIYMDPHYCQSFVDVSIKDFPLETFHCPSPKKMSFRKMDPSCTIGFYCRNVQDFKRASEEIT
KMLKFSSKEKYPLFTFVNGHSRDYDFTSTTTNEEDLFSEDEKKQLKRFSTEEFVLL

>sp|Q86TL0|ATG4D_HUMAN Cysteine protease ATG4D OS=Homo sapiens OX=9606 GN=ATG4D PE=1 SV=1

MNSVSPAAAQYRSSSPEDARRRPEARRPRGPRGPDPNGLGPSGASGPALGSPGAGPSEPDEVDK
FKAKFLTAWNNVKYGWVVKSRTSFSKISSIHLCGRRYRFEGEGDIQRFQRDFVSRLWLTYRRDFPPL

PGGCLTSDCGWGCMLRSGQMMLAQGLLLHFLPRDWTWAEGMGLGPPELSGSASPSRYHGPA
RWMPPRWAQGAPELEQERRHRQIVSWFADHPRAPFGLHRLVELGQSSGKKAGDWYGPSLVAHI
LRKAVESCSDVTRLVVYVSQDCTVYKADVARLVARPDPTAEWKSVVILVPVRLGGETLNPVYVPCVK
ELLRCELCLGIMGGKPRHSLYFIGYQDDFLLYLDPHYCQPTVDVSQADFPLESFHCTSPRKMAFAK
MDPSCTVGFYAGDRKEFETLCSELTRVLSSSSATERYPMFTLAEGHAQDHSLDDLCSQLAQPTLRL
PRTGRLLRAKRPSSEDFVFL

>sp|P53867|ATG4_YEAST Cysteine protease ATG4 OS=Saccharomyces cerevisiae (strain ATCC 204508 / S288c) OX=559292 GN=ATG4 PE=1 SV=2

MQRWLQLWKMDLVQKVSHGVFEGSSEEPAALMNHDYIVLGEVYPERDEESGAEQCEQDCRYRG
EAVSDGFLSSLFGREISSYTKEFLLDVQSRVNFTYRTRFVPIARAPDGPSPLSLNLLVRTNPISTIEDYI
ANPDCFNTDIGWGCMIRTGQSLLGNALQILHLGRDFRVNGNESLERESKFVNWFNDTPEAPFSL
HNFVSAGTELSDKRPGEWFGPAATARSIQSLIYGFPECGIDDCIVSVSSGDIYENEVEKVFAENPNS
RILFLLGVKLGINAVNESYRESICGILSSTQSVGIAGGRPSSSLYFFGYQGNEFLHFDPHIPQPAVEDS
FVESCHTSKFGKLQLSEMDPSMLIGILIKGEKDWQQWKLEVAESAIINVLAKRMDDFDVSCSMDD
VESVSSNSMKKDASNNENLGVLEGDYVDIGAIFPHTTNTEDVDEYDCFQDIHCKKQKIVVMGNTH
TVNANLTDYEVEGVLVEKETVGIHSPIDEKC

>sp|A6ZRL7|ATG4_YEAS7 Cysteine protease ATG4 OS=Saccharomyces cerevisiae (strain YJM789) OX=307796 GN=ATG4 PE=3 SV=1

MQRWLQLWKMDLVQKVSHGVFEGSSEEPAALMNHDYIVLGEVYPERDEESGAEQCEQDCRYRG
EAVSDGFLSSLFGREISSYTKEFLLDVQSRVNFTYRTRFVPIARAPDGPSPLSLNLLVRTNPISTIEDYI
ANPDCFNTDIGWGCMIRTGQSLLGNALQILHLGRDFRVNGNESLERESKFVNWFNDTPEAPFSL
HNFVSAGTELSDKRPGEWFGPAATARSIQSLIYGFPECGIDDCIVSVSSGDIYENEVEKVFAENPNS
RILFLLGVKLGINAVNESYRESICGILSSTQSVGIAGGRPSSSLYFFGYQGNEFLHFDPHIPQPAVEDS
FVESCHTSKFGKLQLSEMDPSMLIGILIKGEKDWQQWKLEVAESAIINVLAKRMDDFDVSCSMDD
VESVSSNSMKKDASNNENLGVLEGDYVDIGAIFPHTTNTEDVDEYDCFQDIHCKKQKIVVMGNTH
TVNANLTDYEVEGVLVEKETVGIHSPIDEKC

>sp|Q2U5B0|ATG4_ASPOR Probable cysteine protease atg4 OS=Aspergillus oryzae (strain ATCC 42149 / RIB 40) OX=510516 GN=atg4 PE=3 SV=2

MNSVDIGRCRKRIVQYIWDPEPRNDEEPDASIWCLGVEYAPQPQKITANTTPGKLGNYQDELEAGT
SKIDDVTAHGWPEAFVSDFESKIWMTYRSDFPPIPRLDNDEANHPMTLTVRIRTQLMDPQGFTSDT
GWGCMIRSGQSLLANAMLTLCLGRDWRRGDKAEEEARLLSLFADHPDAPLSIHRFVKYGAESCG

KHPGEWFGPSATARCIEALSAQCGNIAPRVYVTNDTSDVYEDSFLRVARSGSGSIQPTLILLGTRLGI
DNVTPVYWDGLKAVLQLPQSVGIAGGRPSASHYFIGTQGPHFFYLDPHTTRPAVPYSIDGRLLSKTE
ISTYHTRRLRRIHIQDMDPSMLIGFLVRNEDDWEDWKGRVGSVVGKQIIHVFKGEEATYNQGRRGA
LDEVEALDDA

>sp|Q523C3|ATG4_PYRO7 Cysteine protease ATG4 OS=Pyricularia oryzae (strain 70-15 / ATCC MYA-4617 / FGSC 8958) OX=242507 GN=ATG4 PE=1 SV=2

MDSAVAGAADIGRYGRRIVRMIWDPEPTNDPIANRPAWCLGYEYTLETNITSKTKGEDSKLSTATSS
DQQRPPAQANKVPQMPSAQLPTEAAATALSGNTTPPTPEAALEPTKITSQPAAIDTPPDSVDSSFDS
SMAYDDVPDDGGWPPAFLNDFESRIWMTYRSGFEPIPRSTDPTASSRMSFAMRLKTMADQQAGF
TTDSGWGCMIRTGQSLLANSLLTCRLGRSWRRGQAPDEERKLLSLFADDPRAPYSIHNFVAHGAA
KCGKYPGEWFGPSATARCIHALANATENSFRVYSTGDLPDVYEDSFMEVAKPDGKTFHPTLILISTRL
GIDKINQVYWESLTATLQLPQSVGIAGGRPSSSHYFVGAQRSDEDQGSYLFYLDPHHTRPALPFHE
DPQLYTPSDVDSCHTRRLRRLHIREMDPSMLIGFLILDEENWHAWKSSVKHVQGKSIITVSEHDPS
KGSASGRPSAIDEVETLSDDDGDTVLDG

>tr|A0A0L1J8D6|A0A0L1J8D6_ASPN3 Cysteine protease OS=Aspergillus nomiae NRRL (strain ATCC 15546 / NRRL 13137 / CBS 260.88 / M93) OX=1509407 GN=ANOM_003012 PE=3 SV=1

MNSVDIGRCSKRIVQYLWDPEPRNDQKPDASIWCLGVEYAPRFQKHAANRTPDRDEPDAGTNTID
DVTEHGWPEAFVSDFESKIWMTYRSNFPPIPRPDNDEANHPMTLTVRLRTQLMDPQGFTSDTGW
GCMIRSGQSLLANAMLTLCLGRAWRRGDKPEEEAHLLSLFADHPDAPLSIHRFVKHGAESCGKHP
GEWFGPSATARCIEALSAQCGNIAPRVYVTNDTSDVYEDSFLRVARSGSGSIQPTLILLGTRLGIDNV
TPVYWEGLKAVLQLPQSVGIAGGRPSASHYFIGAQGSHLFYLDPHTTRPALPYSTGGRFPSKEEIST
YHTRRLRRIHIQDMDPSMLIGFLVRNEDDWDDWKGRIGSMMGKPIIHVFKGAETTYNQGRREALD
EVEALDDAE

>tr|A0A0W0CEY3|A0A0W0CEY3_CANGB Cysteine protease OS=Candida glabrata OX=5478 GN=AO440_003078 PE=3 SV=1

METIHNISNRLQEVLATNKSVNVDTDNDSLSSNQEDNEVEKVRHLVVILGEKYSYAVDRNTGIIALM
QWFTTNSEIPEEILNAIRSKLNFTYRTNFEPIERAPDGPSPINPLIMLRINPMDAIENVFNNRECFFTD
VGWGCMIRTGQSLLGNALQRVKSTVKDQPYIYEMDDTKEITDLFKDNTKSAFSLQNFVKCGRIYNKI
APGEWFGPATTATCIRYLIQENPCYGIEACYISVSSGDIFKENIQGMIDRYPNGNILILLGIKLGLDSVH
ERYWGEIKTMLESPFSVGIAGGRPSSSLYFFGYFDDTLLFFDPHNSQTALIDDFDESCHTENFGKLN

FSDLDPSMLLGFLLPCSKWDEFQEFTSLLTIVNVLDGMDQYRDPDLNSNDIGNVELSPQLKLSQTP DAITDDDYVDIGALIQGNSMNINDRDNGYQEVQCKNQQIVIMDSLNETKPLEIEKVLVGQGTNLVN ATTPCREAFPK

>tr|A0A2H0ZMM7|A0A2H0ZMM7_CANAR Cysteine protease OS=Candida auris OX=498019 GN=B9J08_003496 PE=3 SV=1

MPPESSKEESSSSSPTSETAHSSSNSVQTSLDRAHVNEPKNAGSGTDSPSEAQNSFQSVLAQFWQ RIRSPEATDQPQPSGSVVIMGSSYQGTLTSPHITEAIQRRIWFTYRTGFEPIPKAEDGPSPLSFIGSMI VHGANAHGTISGLFDNSNFTSDVGWGCMIRTAQCLLANTLQTVLVKGAVKEGKMPDWNAVNSHV ISLFKDTYKTPYSLHNFVRVASHSPLKVKPGQWFGPSAASLSIKRLSCAANQRGNISPELSVMICES CDLHDDEVKHVFEQSGRPLLLLFPIRLGIETINSIYYPSLFQLLSLRNSMGIAGGKPSSSFYFFGYQD QSLLYLDPHTSQHIDQTNGSYKPATCSTVHISGLDPSMVVGLLVESYQDYEELKAKLKDANKIVHFH ERAPARKSASVPVQDRSMEHVESERDSIEDLGDFVDLGDDIADPISEAEFGNDSFDPSSSFVAKYD VVDGPSPVTVLDDEEEA

>tr|A0A2P7YWW6|A0A2P7YWW6_9ASCO Cysteine protease OS=[Candida] pseudohaemuli OX=418784 GN=C7M61_000108 PE=3 SV=1

MSLDHPEENATTSSRESEVNVSGLGKDIGIAQNATNSQEATESQTTTSDQPNTFQNALAQFWQRL RSPELLEDENNGPVVIMGTSYTGNLTLPEIEHAIQRRLWFTYRTGFEPIPRALDGPSPLSFLGSMIVH GVSPQGALTGFFDNNSFKSDVGWGCMIRTAQCLLANTLQTVLVRAAIAEGNTPDWLAINSQVVAL FQDSYSSPFSLHNFVGVASHLPLKVKPGQWFGPSAASLSIKRLCTKANEDSGSTVSVVPRLNVMIC ESCDLHDDEIKTALKELSPLLLLFPIRLGIETINSIYYPSLFQLLSLPNSMGISGGKPSSSYYFFGYQGE LLLYLDPHSSQQVDETNGSYKPLSCNTVPISGLDPSMVVGLLVESYDDYKALKTELQGRNKIVHFH DRAPARRSVSHSTYERSTETERKGETAGDLSDFVDIGDDFGSEEEGPEDTSHSMAAKYDIVEGPSK VTVLEDEN

>tr|A0A2V1A6D2|A0A2V1A6D2_9ASCO Cysteine protease OS=[Candida] duobushaemuli OX=1231522 GN=CXQ87_001762 PE=3 SV=1

MSLDHSEENATTSSRESEVNVSGLGKDVGIAQNATNPQAATESQTTTSDQPNTFQNALAQFWQRL RSPELSEDEINGPVVIMGTSYTGNLTSPEIEHAIQRRLWFTYRTGFEPIPRSLDGPSPLSFLGSMIVHG VNPHGAFTGFFDNNSFKSDVGWGCMIRTAQCLLANTLQTVLVRAAIAEGNTPDWSAINSQVVALF QDSYSSPFSLHNFVGVASHSPLKVKPGQWFGPSAASLSIKRLCTKANENSGSTVSVVPRLNVMICE SCDLHDDEIKTALKEMSPLLLLFPIRLGIETINSIYYPSLFQLLSLTNSMGISGGKPSSSYYFFGYQGES LLYLDPHSSQQVDETNGSYKPLSCNTVPISGLDPSMVVGLLVESYDDYQSLKTELQGRNKIVHFHD

RAPARRSVSHSTHERSTETDRKGENAGDLSDFVDIGDDFGSEEEGAEDTSHSMAAKYDIVEGPSKV
TVLEDEN

>tr|A0A2V1API7|A0A2V1API7_9ASCO Cysteine protease OS=[Candida] haemuli OX=45357 GN=CXQ85_003539 PE=3 SV=1

MSSEPSKEEHKASLAEPVPSAASDSKVPLETSPPHSNLQGPQEIVDSGTKSAAEQPNSFQNALAQF
WQRLRSPDAPETDPESLQPVVIMGTSYKGTLTSAGIEEAIRRRLWFTYRTGFEPIPRALDGPSPLSFIG
SMIVHGVNPHGALAGFLDNTSFTSDVGWGCMIRTAQCLLANTLQTVIARKAIQDGNTPDWSSINT
QVVSLFRDNYNSPFSLHNFVGVASHSPLKVKPGQWFGPSAASLSIKRLCSKANEDADFPKLNVMI
CESCDLHGDEVKSLLEKTSRPLLLLFPIRLGIETINAIYYPSLFQLLSLPHSMGISGGKPSSSYYFFGH
QGDSLLYLDPHTSQQIDETNGSYRPLSCNTVPISGLDPSMVVGLLVESYADYEALKSELKGKNKIVH
FHDRAPTRRSVSVSNQDKNAKSAEPDGNGASDIGDFVDIGDDFAGSEVEEEIQEDPNSSVTAKYDI
VEGPAPVTVLEDEK

>tr|A0A367XZI9|A0A367XZI9_9ASCO Cysteine protease OS=Candida viswanathii OX=5486 GN=ATG4_0 PE=3 SV=1

MSKEPTENTSEKTPTNPPPSQDSVEEVDTVLGRFSLFVKDLSNGISNPSRDGTPSEEPATVIHKPTPII
VLGKEFDDQQEAASFIESKLWLSYRCGFDPIPKSDDGPQPIQFFPSIIFNKTTIYSNFANLKGLFDKD
NFTSDAGWGCMIRTSQNLLANTLLLLLPPDSKEDIIGLFQDRETAPFSIHNFIRVASELPLQVKPGQ
WFGPNAASLSIKRLTDGLQDTEIKGVKYPKVFISENCDLYDDEMQEILKEEGRSVLILLPIRLGIDKVN
AYYYNSIFQILKSKYSCGISGGKPSSSFYFLGYEDSELIYFDPHLPQLVEDLTNLESYQTRNYNKLNIS
LLDPSMMIGILLQSMDDYLDFKTSCIDNSNKIVHFHPHVLPSQQQDTLINQSWEEVHDEDEDFVN
LNVNKVEDEEVQEVPEEPKKLDEFIDLGKQEQN

>tr|A0A367Y9D2|A0A367Y9D2_9ASCO Cysteine protease OS=Candida viswanathii OX=5486 GN=ATG4_1 PE=3 SV=1

MPNELTENTNETTPTNPPPSQDSVEEVDTVLGRFSLFVKDLSNGISNPSRDGTPSEEPATVIHKPTPII
VLGKEFDDQQEAASFIESKLWLSYRCGFDPIPRSDDGPQPIQFFPSIIFNKTTIYSNFANLKGLFDKD
NFTSDAGWGCMIRTSQNLLANTLLLLLPPDSKGDIIGLFQDTETAPFSIHNFIRVASELPLQVKPGQ
WFGPNAASLSIKRLTDGLQDTEIKGVKYPKVFISENCDLYDDEIKEILKEEGRSVLILLPIRLGIDKVNA
YYYNSIFQILKSKYSCGISGGKPSSSFYFLGYEDSELIYFDPHLPQLVETPTNLESYQTRNYSKLNISLL
DPSMMIGILLQSMDDYLDFKTSCIDNSNKIVHFHPHVLPSQQQQDTLINQSWEEVQDEDEDFVNL
NVNKVEDEEEQEVPEELKKLDEFIDLGKQEQN

>tr|A0A3F3RUT5|A0A3F3RUT5_ASPNG Cysteine protease OS=Aspergillus niger OX=5061 GN=AnigIFM63604_005678 PE=3 SV=1

MNTVDIGRCSKRIVQYLWDPEPRNDEDPNSSIWCLGIEYHPDKDANTRETPDKNNTRENVMGTT
NYRKPSEHAWPESFLLDFESRIWMTYRSNFPPIPRVEGDDKSASMTLGVRLRSQLVDTQGFTSDTG
WGCMIRSGQSLLANALSMLVLGRDWRRGARFEEESQLLSLFADTPTAPFSVHRFVKHGAESCGKY
PGEWFGPSATAKCIEALSSQCGNPTLKVYVSNDTSEVYQDKFMDIARNTSGAFQPTLILLGTRLGID
NITPVYWDGLKAALQFPQSVGIAGGRPSASHYFVGAQGSHLFYLDPHYTRPALPDRQEGELYSKEE
VDTYHTRRLRRIHVRDMDPSMLIGFLIRNQEDWADWLKRIEAVKGRPIIHVLKQMNPDHDQEAGA
LDQVEALDDIE

>tr|A0A8H7YCI2|A0A8H7YCI2_9EURO Cysteine protease OS=Histoplasma ohiense OX=2902605 GN=ATG4 PE=3 SV=1

MNNVDIGKYKRIVQYLWDPEPKNDDEPGSPIWCLGREYQPLTPPQPQPPSPHVQAVDTGTKDAG
DDKKTQDEQKSHLDQTTNTSPTRSSDSATKPQRHLLPFAIHRGSTTSPLGQQGQQHWPDAFLDD
FESKIWLTYRSNFPLIPKSSDPNALSAMTLGVRLRSQLVDSQGFTTDTGWGCMIRSGQSLLANTLAI
LSLGRDWRRGTKIKEESKLLSLFADDPKAPFSIHRFVEHGASACGKYPGEWFGPSATARCIQALSSE
CEHAGLNVYVTSDGSDVYEDRFRAIASAGGAGAGTSTDVHPTLILLGIRLGIDRVTPVYWEALKAVLK
YPQSVGIAGGRPSSSHYFIGAQGSHFFYLDPHHTRPALAYHDAGDRPYTTEELNTYHTRRLRRLHIK
DMDPSMLIGFLIRNEDDWNSWKRSVHNGAMIGTGKAIIHVFDKEKSPFGGHGPEREGAVDEVEAL
DDDDDDDDDDDDDVDVDKCDDRDVRVDDDNDVSIREDAGDSSSFLHISK

>tr|A0A9P6XEZ8|A0A9P6XEZ8_RHIOR Cysteine protease OS=Rhizopus oryzae OX=64495 GN=G6F64_003077 PE=3 SV=1

MEDNVPPFGLSVNEWSFYQYVLASPKGTKFTDRPESMDVQDAYIAMNSLLKKNALEIIIVNNENVL
KAIDLTDAEKASRLNDDARLVYNAIRSAGNTGIWTKDLKKATNLHTIVLNRTLKQLEQKREIKSIKHVK
YPTRKIYMLFNLTPSSEVTGGAWYTDQELDIDFIDSLKAACLKYITSRSFPRAEGIPDAVFGAEHEHYP
TASEVKRFITESRISSIELSVKDIETDMEDFSDEEEDNGLVQWSYKAIKKAVNRLSTETFTETPCGKCP
VFSFCVEDGPISPMNCEYYKVWLNCSDQKMSESCTDLADLEVNDKELPQPQNSNHLSTAPLKDK
LLTNSSYLAAEIPLKFGHFMSNLWTSGSELSSSLFLQQQQQQDNCIIWLLGCSYIIKPTDHIQQAL
LEAQRDLMFNKSSSENEEENNQNMYMLWPPDFYDDFTSRLWMTYRHNYPPIRPSSHKTDIGWG
CTLRSGQSLLANTLIIHFLGRDWRRQTQNQAAWKQYSRIVHWFLDELSPRAPFSIHRIALLGKQLG
KNIGEWFGPSTISQVIQALVSDFAPANLSVYVAADGVIYRDDVNDVATGKKPRGDFSYLSSLADSKG
REEAKHLYDASTPQSSTVKTDTFKPVLILVALRLGIDSLHPTYYGGLKACFELPSFVGIVGGRPNSSLY

FIGLKGDELIYLDPHYSRPALETKSLAQYTRKDFNTYHCTIPRKVHISNIDPSMLVGFYCQNISDFDSL
CQQITKTSKNHSAIISVEQSAPVYEEDVRSENDFGIVSDEDDDSDVDDIADK

>sp|A1CJ08|ATG4_ASPCL Probable cysteine protease atg4 OS=Aspergillus clavatus (strain ATCC 1007 / CBS 513.65 / DSM 816 / NCTC 3887 / NRRL 1 / QM 1276 / 107) OX=344612 GN=atg4 PE=3 SV=1

MNSVDIGRYKRIINYLWDPEPRNDLPDEPIWCLGIRYPPNHRGWKTQDQDGSAQGQYEQKTIPTE
KANEHQWPEEFLDDVESRIWITYRSNFTPIPKPPNQEANPAMTLTVHLRSQLMDSQGFTSDTGWG
CMIRSGQSLLANAMLILLLGRDWRRGTEAGKEAQLLHQFADHPEAPFSIHRFVQHGAEFCNKYPG
EWFGPSATARCIQALVAQQGSSELRVYITDDTADIYEDKFARIAQAEHGDFIPTLILVGTRLGIDHVTP
AYWDALKEALQLPQSVGIAGGRPSASHYFIGVHGQYLFYLDPHHTRPASLHQDVNDTLTHEEVNTY
HTRRLRRIHIKDMDPSMLIGFIIRSREDWTDWKTRILSGRGNSIVHILSEDTANHQARKEAIDEVEAL
DD

>sp|A2QY50|ATG4_ASPNC Probable cysteine protease atg4 OS=Aspergillus niger (strain ATCC MYA-4892 / CBS 513.88 / FGSC A1513) OX=425011 GN=atg4 PE=3 SV=1

MNTVDIGRCSKRIVQYLWDPEPRNDEDPNSSIWCLGIEYHPDKDANTRETPDKNNTRENVMGTT
NYRKPSEHAWPESFLLDFESRIWMTYRSNFPPIPRVEGDDKSASMTLGVRLRSQLVDTQGFTSDTG
WGCMIRSGQSLLANALSMLVLGRDWRRGARFEEESQLLSLFADTPTAPFSVHRFVKHGAESCGKY
PGEWFGPSATAKCIEALSSQCGNPTLKVYVSNDTSEVYQDKFMDIARNTSGAFQPTLILLGTRLGID
NITPVYWDGLKAALQFPQSVGIAGGRPSASHYFVGAQGSHLFYLDPHYTRPALPDRQEGELYSKEE
VDTYHTRRLRRIHVRDMDPSMLIGFLIRNQEDWADWLKRIEAVKGRPIIHVLKQMNPDHDQEAGA
LDQVEALDDIE

>sp|A6SDQ3|ATG4_BOTFB Probable cysteine protease atg4 OS=Botryotinia fuckeliana (strain B05.10) OX=332648 GN=atg4 PE=3 SV=1

MTAADLGRYKRFVQYFWDPEPTNDTASQSPIWCLGKEYPILEKSATSAITDSPPQEGHYLPAQSLPT
NEVTTPPDSTVGSLESSSGSQNCDTANADGGWPSAFLDDFEAKIWLTYRSNFPAIAKSQDPKALSA
MSLSVRLRSQLVDQGGFTSDTGWGCMIRSGQSLLANALLTLRMGREWRRGVSSNEERKILSLFAD
DPRAPYSIHKFVEHGASACGKHPGEWFGPSATARCIQALSNSQAKSELRVYITGDGSDVYEDKFM
SIAKPNHSDFTPTLILVGTRLGLDKITPVYWEALKYSLQMPQSVGIAGGRPSSSHYFIGVQESDFFYL
DPHQTRPALPYKDNVEDYTTEDIDSCHTRRLRRLHIKEMDPSMLIAFLIRDENDWNEWRRAVKEVQ
GKGVIHVADTDPASYGLGGERDGAIDEVETFDDDDDDTILDA

>sp|A7F045|ATG4_SCLS1 Probable cysteine protease atg4 OS=Sclerotinia sclerotiorum (strain ATCC 18683 / 1980 / Ss-1) OX=665079 GN=atg4 PE=3 SV=2

MTTVDIGRYKRLVQYFWDPEPTNNIASKSPIWCLGEEYLVSDKSSPSAVTESPPKEGGYLLAQSLSTT
ETTTPPDSTVGSLESSSEYDNCDTASTDGGWPTAFLDDFEAKIWLTYRSNFPAIAKSQDPKALSAMS
LSVRLRSQLVDQGGFTSDTGWGCMIRSGQSLLANALLTLRMGREWRRGSSSNEERKILSLFADDP
RAPYSIHKFVEHGASACGKHPGEWFGPSAAARCIQALTNSQVESELRVYITGDGSDVYEDTFMSIA
KPNSTKFTPTLILVGTRLGLDKITPVYWEALKSSLQMPQSVGIAGGRPSSSHYFIGVQESDFFYLDPH
QTRPALPFNDNVEDYTPEDIDSCHTRRLRRLHIKEMDPSMLIAFLIRDENDWKDWRRAVREVQGK
GVIHVADRDPALHGLGAERDGAIDEVETFDDDDDDTVLNG

>tr|B3LP67|B3LP67_YEAS1 Cysteine protease OS=Saccharomyces cerevisiae (strain RM11-1a) OX=285006 GN=SCRG_03349 PE=3 SV=1

MQRWLQLWKMDLVQKVSHGVFEGSSEEPAALMNHDYIVLGEVYPERDEESGAEQCEQDCRYRG
EAVSDGFLSSLFGREISSYTKEFLLDVQSRVNFTYRTRFVPIARAPDGPSPLSLNLLVRTNPISTIEDYI
ANPDCFNTDIGWGCMIRTGQSLLGNALQILHLGRDFRVNGNESLERESKFVNWFNDTPEAPFSL
HNFVSAGTELSDKRPGEWFGPAATARSIQSLIYGFPECGIDDCIVSVSSGDIYENEVEKVFAENPNS
RILFLLGVKLGINAVNESYRESICGILSSTQSVGIAGGRPSSSLYFFGYQGNEFLHFDPHIPQPAVEDS
FVESCHTSKFGKLQLSEMDPSMLIGILIKGEKDWQQWKLEVAESAIINVLAKRMDDFDVSCSMDD
VESVSSNSMKKDASNNENLGVLEGDYVDIGAIFPHTTNTEDVDEYDCFQDIHCKKQKIVVMGNTH
TVNANLTDYEVEGVLVEKETVGIHSPIDEKC

>tr|B6VCT6|B6VCT6_PYROR Cysteine protease OS=Pyricularia oryzae OX=318829 GN=ATG4 PE=2 SV=1

MDSAVAGAADIGRYGRRIVRMIWDPEPTNDPIANRPAWCLGYEYTLETNITSKTKGEDSKLSTATSS
DQQRPPAQANKVPQMPSAQLPTEAAATALSGNTTPPTPEAALEPTKITSQPAAIDTPPDSVDSSFDS
SMAYDDVPDDGGWPPAFLNDFESRIWMTYRSGFESIPRSTDPTASSRMSFAMRLKTMADQQAGF
TTDSGWGCMIRTGQSLLANSLLTCRLGRSWRRGQAPDEERKLLSLFADDPRAPYSIHNFVAHGAA
KCGKYPGEWFGPSATARCIHALANATENSFRVYSTGDLPDVYEDSFMEVAKPDGKTFHPTLILISTRL
GIDKINQVYWESLTATLQLPQSVGIAGGRPSSSHYFVGAQRSDEDQGSYLFYLDPHHTRPALPFHE
DPQLYTPSDVDSCHTRRLRRLHIREMDPSMLIGFLILDEENWHAWKSSVKHVQGKSIITVSEHDPS
KGSASGRPSAIDEVETLSDDDGDTVLDG

>tr|C5M3Z0|C5M3Z0_CANTT Cysteine protease OS=Candida tropicalis (strain ATCC MYA-3404 / T1) OX=294747 GN=CTRG_00779 PE=3 SV=1

MQDESTDKNANKSPPQSQDSVEEVDTVLGRFSLFVKDLSQGFSNPSRDGTPSEEPATLVDTPQPVIVLGQEFKNEQEAIVFIESRLWLSYRCGFDPIPKAEDGPQPIQFFPSIIFNKTTIYSNFANLKSLFDKENFTSDAGWGCMIRTSQNLLANTLLQLLPPDSKQDVIGLFQDNQSSPFSIHNFIKVAGESPLQVKPGQWFGPNAASLSIKRLTDTLQDKEIKGVKYPKVFISENSDLYDGEINEILSEEGRSVLVLFPIRLGIDKVNSYYYDSIFQVLKSKFSCGISGGKPSSSFYFLGYDNSDLIYFDPHLPQLVENPINIESYHTRNYNRLNISLLDPSMMIGILLRSMDDYLEFKTSCIGNNNKIVHFHPHVSPLQQQQDNLINQSWEEVQDEDEDFVNLNVSKVEVAEEETEDEEGVKVDSPKQSDEFIDLGKQEQS

>tr|G2YW47|G2YW47_BOTF4 Cysteine protease OS=Botryotinia fuckeliana (strain T4) OX=999810 GN=BofuT4_P154070.1 PE=3 SV=1

MTAADLGRYKRFVQYFWDPEPTNDTASQSPIWCLGKEYPILEKSATSAITDSPPQEGHYLPAQSLPTNEVTTPPDSTVGSLESSSGSQNCDTANADGGWPSAFLDDFEAKIWLTYRSNFPAIAKSQDPKALSAMSLSVRLRSQLVDQGGFTSDTGWGCMIRSGQSLLANALLTLRMGREWRRGVSSNEERKILSLFADDPRAPYSIHKFVEHGASACGKHPGEWFGPSATARCIQALSNSQAKSELRVYITGDGSDVYEDKFMSIAKPNHSDFTPTLILVGTRLGLDKITPVYWEALKYSLQMPQSVGIAGGRPSSSHYFIGVQESDFFYLDPHQTRPALPYKDNVEDYTTEDIDSCHTRRLRRLHIKEMDPSMLIAFLIRDENDWNEWRRAVKEVQGKGVIHVADTDPASYGLGGERDGAIDEVETFDDDDDDTILDA

>tr|G8BC15|G8BC15_CANPC Cysteine protease OS=Candida parapsilosis (strain CDC 317 / ATCC MYA-4646) OX=578454 GN=CPAR2_802300 PE=3 SV=1

MTEPQPASESQQDIEENATNQPRSYDVAPGSGFERLTSFFKGVSGINMGSQEAKDDTHSTTYNMNTEQKSISILGNHFKTETEAKEYIQSLLWLSYRCGFTPIPKSADGPQPVSFLPSVLFSKSTLTNMSNLRGLFDNDNFTSDAGWGCMIRTSQNLLAIALLKLSEEHNESAQLDILKLFQDDPTSPFSLHNFIRVASSSPLLVKPGQWFGPNAASLSIKKLTIEAKKLETPGEIPYVYISENADLFDDEIEDLFNEEQKPLLLLFPVRLGIDQVNKYYYKSILQLLSLPYSVGIAGGKPSSSFYFIGYENENHLLYFDPHLPQVVEAPINITTYHTANYNKLDIEMVDPSMMIGVLLKSMDEYKEFKQDCSENKIIHFHPLVITSQQDSALNQSWEEVQEDDDFVNLTVPKSEEEFVVLDK

>tr|M7TFD4|M7TFD4_BOTF1 Cysteine protease OS=Botryotinia fuckeliana (strain BcDW1) OX=1290391 GN=BcDW1_9232 PE=3 SV=1

MTAADLGRYKRFVQYFWDPEPTNDTASQSPIWCLGKEYPILEKSATSAITDSPPQEGHYLPAQSLPT
NEVTTPPDSTVGSLESSSGSQNCDTANADGGWPSAFLDDFEAKIWLTYRSNFPAIAKSQDPKALSA
MSLSVRLRSQLVDQGGFTSDTGWGCMIRSGQSLLANALLTLRMGREWRRGVSSNEERKILSLFAD
DPRAPYSIHKFVEHGASACGKHPGEWFGPSATARCIQALSNSQAKSELRVYITGDGSDVYEDKFM
SIAKPNHSDFTPTLILVGTRLGLDKITPVYWEALKYSLQMPQSVGIAGGRPSSSHYFIGVQESDFFYL
DPHQTRPALPYKDNVEDYTTEDIDSCHTRRLRRLHIKEMDPSMLIAFLIRDENDWNEWRRAVKEVQ
GKGVIHVADTDPASYGLGGERDGAIDEVETFDDDDDDTILDA

>sp|P0CQ10|ATG4_CRYNJ Cysteine protease ATG4 OS=Cryptococcus neoformans var. neoformans serotype D (strain JEC21 / ATCC MYA-565) OX=214684 GN=ATG4 PE=3 SV=1

MSSPTSTSPKSSFVFSPTSFPHAAIASNNVRPRTNLPPPPPPDRIPPPKGRSHQQKFKILRKEKDKD
RRQPIDLEDDWTIEDVTVNGDGVEQESQYLDDLQPEENELKPGPRFLEMANREEKKEKTSKSRGL
VKKTSRLFGRDKDKDRGKPEEPAVTGSSSSTLAAMRQSSSTSTDSTTSRSITSAFTRQNSIQSRRSPR
TSFGQAHSRRASQDSQMSWPAPRSIRSSTTSHDPSSDPQNSASSTGVPIPQRQGASMSSLSRYSL
PHPNGGTSRSPDTFPNKMSTWFSHLLPVSSGSPPSSSYETSSSIRKQSSVAASLFNAARQKAVDGV
RHLLDSEAQPDKCMDTIWVRGVAHPGWRPITPENSTSNLPALEPGGSGGGVEDRRASLSMNGPS
PNSLRPSSWKRNTSLPPTGQPQSPAHVHTQASNQTTSPSKGFTGIWNPSTLSLGMPIGGSPNKEK
ENGSGAESPSKKKSKEIVKWPEQFYDDFKSTVWFTYRNQYAPISSLSPNLLIPSPEAYYASFGPPLD
ATSPSSLRVTTPTAAAQQSASGSGGWGWSKEERGLTSDAGWGCMLRTGQSLLVNALIHIHLGRD
WRVPSTPASFSEATTTQEIAALKDYAKYAQMLSWFLDDPSPLCPFSVHRMALIGKELGKEVGEWFG
PSTAAGALKTLANSFAPCGVAVATATDSIIYKSDVYTASNLPSDDWNSISPTFNSSKKKRRGDNEAKE
EKWGKRAVLILVGVRLGLDGVNPIYYDSIKALFTFPQSVGIAGGRPSSSYYFVGSQANHLFYLDPHLT
RPAIPLQIPPLPVHSAKEKGSTESSSIMSTAEEESEEGVMIRTPETPRSTTPSMFSAPEHVEDEDQEE
WGQGSKYKLDVVDADGVEVEGIDDDKGRNEGKMIREEVPKSSFESNGAAQEQPKKQKGFTSTASI
DSQVDSQVDPHMLWYTTAYPDPLLRTYHCEKIKKMPLSGLDPSMLLGFVCKDEDDFEDFVERVAQ
LPKKIFTVQDEMPSWEEDDDAGLESVSEPDFEGDEFEEPGTAKPRFDSSSPVNEDSLKGPRVVSAS
TTATPLAAKEEDHLDVEEANSTDDDNESIGTTTAAGPMDIARHLNRVDLSSKREQEGDDDDGEWV
GGTPSSQGVLVEPPSLKGTPSKSRSSAFEPRYEQNGETEQERPVFPARNRMESWVEPVCEGKEAP
NGDNLL

>sp|P0CQ11|ATG4_CRYNB Cysteine protease ATG4 OS=Cryptococcus neoformans var. neoformans serotype D (strain B-3501A) OX=283643 GN=ATG4 PE=3 SV=1

MSSPTSTSPKSSFVFSPTSFPHAAIASNNVRPRTNLPPPPPPDRIPPPKGRSHQQKFKILRKEKDKD
RRQPIDLEDDWTIEDVTVNGDGVEQESQYLDDLQPEENELKPGPRFLEMANREEKKEKTSKSRGL
VKKTSRLFGRDKDKDRGKPEEPAVTGSSSSTLAAMRQSSSTSTDSTTSRSITSAFTRQNSIQSRRSPR

TSFGQAHSRRASQDSQMSWPAPRSIRSSTTSHDPSSDPQNSASSTGVPIPQRQGASMSSLSRYSL
PHPNGGTSRSPDTFPNKMSTWFSHLLPVSSGSPPSSSYETSSSIRKQSSVAASLFNAARQKAVDGV
RHLLDSEAQPDKCMDTIWVRGVAHPGWRPITPENSTSNLPALEPGGSGGGVEDRRASLSMNGPS
PNSLRPSSWKRNTSLPPTGQPQSPAHVHTQASNQTTSPSKGFTGIWNPSTLSLGMPIGGSPNKEK
ENGSGAESPSKKKSKEIVKWPEQFYDDFKSTVWFTYRNQYAPISSLSPNLLIPSPEAYYASFGPPLD
ATSPSSLRVTTPTAAAQQSASGSGGWGWSKEERGLTSDAGWGCMLRTGQSLLVNALIHIHLGRD
WRVPSTPASFSEATTTQEIAALKDYAKYAQMLSWFLDDPSPLCPFSVHRMALIGKELGKEVGEWFG
PSTAAGALKTLANSFAPCGVAVATATDSIIYKSDVYTASNLPSDDWNSISPTFNSSKKKRRGDNEAKE
EKWGKRAVLILVGVRLGLDGVNPIYYDSIKALFTFPQSVGIAGGRPSSSYYFVGSQANHLFYLDPHLT
RPAIPLQIPPLPVHSAKEKGSTESSSIMSTAEEESEEGVMIRTPETPRSTTPSMFSAPEHVEDEDQEE
WGQGSKYKLDVVDADGVEVEGIDDDKGRNEGKMIREEVPKSSFESNGAAQEQPKKQKGFTSTASI
DSQVDSQVDPHMLWYTTAYPDPLLRTYHCEKIKKMPLSGLDPSMLLGFVCKDEDDFEDFVERVAQ
LPKKIFTVQDEMPSWEEDDDAGLESVSEPDFEGDEFEEPGTAKPRFDSSSPVNEDSLKGPRVVSAS
TTATPLAAKEEDHLDVEEANSTDDDNESIGTTTAAGPMDIARHLNRVDLSSKREQEGDDDDGEWV
GGTPSSQGVLVEPPSLKGTPSKSRSSAFEPRYEQNGETEQERPVFPARNRMESWVEPVCEGKEAP
NGDNLL

>sp|Q1E5M9|ATG4_COCIM Probable cysteine protease ATG4 OS=Coccidioides immitis (strain RS) OX=246410 GN=ATG4 PE=3 SV=1

MNTVDIGQKYKRIVEYLWDPEPKNDDDIIEPVWCLGKEYKTSIPRDSEGAEAPESCNMPGMPFLSP
MNQMSLSSRDTQAALSKPATPPHQLGIQRSKSREWPTSFLDDFESKFWFTYRSNFPAIPKSRDPDT
PLALTLSVRLRSQFLDTHGFTADTGWGCMIRSGQSLLANALSILNLGRDWRRGSKIKEECELLSLFA
DNPQAPFSIHRFVDYGASACGKHPGEWFGPSATARCIEALSNECKHTDLNVYVMSDGSDVHEDQ
FRQIAGPDGIRPTLILLGVRLGIESVTPVYWEALRAIIRYPQSVGIAGGRPSSSLYFIGVQGPYFFYLDP
HHTRPAVSWNPDSTLSPENLDTYHTRRLRRLHIREMDPSMLIGFLIKDDDDWKDWKRRLRSVTGN
PIIHIFDLERPNFGRHLEREEAVDEVEALDDDSN

>sp|Q59UG3|ATG4_CANAL Cysteine protease ATG4 OS=Candida albicans (strain SC5314 / ATCC MYA-2876) OX=237561 GN=ATG4 PE=3 SV=2

MNQPNPNKQPIVQSTSEQTSNEEVDTVLGRFTLFVKDLSNGLNGSQEVPPSQESVSEEAEVISRKII
VLGQTFDNFDNANDYIESKLWLSYRCGFEPIPKSIDGPQPIQFFPSIIFNRSTIYSNFANLKSLFDKEN
FTSDAGWGCMIRTSQNLLANTLLKLYPKNEPEIVKLFQDDTSSPFSIHNFIRVASLSPLHVKPGEWF
GPNAASLSIKRLASELLQDQEIDGIKIPRVFISENSDLFDDEIRDVFAKEKNASVLILFPIRLGIDKVNS
YYYNSIFHLLASKYSCGIAGGKPSSSFYFLGYEDTDLIYFDPHLPQVVETPINMDSYHTTNYNRLNISL

LDPSMMIGILVTNIDEYIDFKTSCLDINNKIVHFHPHTLPVQQDSIINQSWEEVQDEEEEFINLNVSKI
ENEQQQEQGQSTDAPDEFIDIGNQSSSVVSVPSNV

>sp|Q6FP20|ATG4_CANGA Probable cysteine protease ATG4 OS=Candida glabrata (strain ATCC 2001 / BCRC 20586 / JCM 3761 / NBRC 0622 / NRRL Y-65 / CBS 138) OX=284593 GN=ATG4 PE=3 SV=1

METIHNISNRLQEVLATNKSVNVDTDNDSLSSNQEDNEVEKVRHLVVILGEKYSYAVDRNTGINAL
MQWFTTNSEIPEEILNAIRSKLNFTYRTNFEPIERAPDGPSPINPLIMLRINPIDAIENVFNNRECFFTD
VGWGCMIRTGQSLLGNALQRVKSTVKDQPYIYEMDDTKEITDLFKDNTKSAFSLQNFVKCGRIYNKI
APGEWFGPATTATCIRYLIQENPCYGIEACYISVSSGDIFKENIQGMIDRYPNGNILILLGIKLGLDSVH
ERYWGEIKTMLESPFSVGIAGGRPSSSLYFFGYFDDTLLFFDPHNSQTALIDDFDESCHTENFGKLN
FSDLDPSMLLGFLLPCSKWDEFQEFTSLLTIVNVLDGMDQYRDPDLNSNDIGNVELSPQLKLSQTP
DAITDDDYVDIGALIQGNSMNINDRDNGYQEVQCKNQQIVIMDSLNETKPLEIEKVLVGQGTNLVN
ATTPCREAFPK

>tr|A0A095CCL1|A0A095CCL1_CRYD2 Autophagy-related protein 4 OS=Cryptococcus deuterogattii (strain R265) OX=294750 GN=CNBG_3127 PE=3 SV=1

MSSPTPTSPTSSLAFSPTSFSHASIASTNVRSRTNLPPPPPPDRIPPPKGRPHQQKFKILRKGKDKDK
RQPMNSEDDWAIEDVVANGDSIEQEPQYLDDLQSEENESKPEQQLVEMASREEKKEKASKSRELV
RKTSRLFGRDKDKDRDKFEEPAVTGSSSSTLAATRQSSSTSTDSSTSRSFTSAFTRMNSIQSKRSPRR
SFGQAHSRRASQDSQMSWPAPRSIHSSITSHDTSGDPQDGAGGNSVPIPQRQGASMSSLSRYSL
PQPNGNTSRSPDTFPNKMSTWFSHLLPVSSETPSPSTNETASSVRKQPSVAASLFNAARQKAVDG
VRHLLDSEAQPDKCMDTIWVRGVAHPGWRPITPENVASNLPALEPVDGGVEDRRASVSMNRPSP
TSFRPSSWRRNTSLPPSAQLQPSTQIHSQASNQTTSPNKGFTGIWNPSTLSLAMSIGGSPNKEKEN
GSGAESPSKKKGKEVVKWPEQFYDDFKSIVWFTYRNQYAPILSLSPDLLIPSPEAYYASFGPPLDATS
PSSPQVTTPTATAQQTATGGGGWSWSKEERGLTSDAGWGCMLRTGQSLLVNALIHVHLGRDWRL
PSTPSIFSEATTSQEIAALKDYAKYAQMLSWFLDDPSPLCPFSVHRMALIGKELGKEVGEWFGPSTA
AGTLKTLANSFAPCGVAVATATDSIIYRSDVYAASNLPSDDWNRISPTFYPNRKKKRDNAEAKEGKW
GERAVLILVGIRLGLDGVNPIYYDSIKALFTFPQSVGIAGGRPSSSYYFVGSQANHLFYLDPHLTRPAI
PLQVPPLPVHSAKEEGSMESSSILSTAEEESEEGVMIHTPETPRSTTPSTFSAAEEVEDEEREEWEQG
SKYKLDVMDADGFRVEEGISDDKGRNEGTSIGSDVPKSTLESNGVIQERSDKQNFFTSTTSTDSQV
DPQMLWYTTAYPDSLLRTYHCEKIKKMPLSGLDPSMLLGFVCKSEDDFENFVERVAQLPKKIFTVQ
DEMPSWGEDDDAGLESVSEPDFEEDEFEEPGTAKPRFDSSSPLNEDSLKGPSIVSASTTATPIAAKE
DYLDGEDGDGTDDDDESIGTTTAVRPVDIVRHLHRVDLSVKREQEGGDDDDGEWVGGTPSSQG
VLVEPLSLKGTPSKSRSSTVEPHHEQSEEEQERRVFPVRNRMESWVEPVCEGKEAPNGDNLL

>tr|A0A099P6Z5|A0A099P6Z5_PICKU Autophagy-related protein 4 OS=Pichia kudriavzevii OX=4909 GN=JL09_g872 PE=3 SV=1

MGSQDIADGNDVYFSGLQNMWDRIWEFPGGLDKCSSESDLVILGMHYKVNDENQEDEKSEEQS
GISKCAGRATYEKIENSENSEDQLINVNYDKISDDKNTGDEPEKFDNNAIDNIKMFMKKISDTTYQK
FHSELNYTYPQNALDDLQTRLWFTYRYGFPLIKYDENGSPPIHLGAILRGNMDIQNFGKGFTSDSG
WGCMIRTSQTLLANALVSLKLGRNWRLQGSAPEDMKVHWEIVEQFADSPDARFSIHNMVLYAAKY
CGRRPGEWFGPSNAARSIEMLCNESQVDTNLKVYISSNSGDIYEEDLLSSCYEKNIDKFTPVLILCG
VRLGVHNINKIYWEFLKLTLDLPYSVGIAGGRPSSSHYFLGCQADYLLYLDPHIPQQAILLNDLKKAG
SSDYKSKMLGTLHTTKIKLLSLGKVDPSMLIGFLVKTREEYNDLRQKINLFPSNRRFLNFSNSKPVIRR
TSSVEDDIDGFVDLTMESGDEFLEHGEIIDDIDNKIKDMAEDKHQELAEYEKLEHAEIKDEPIVEIEKI
DTPVKLQSERSGKYNNEIFASDGIIEVEDINNENISGSRLERLKQLAMSTIEDDVFYEQDELDFEELIE
KNMDSNFEDDENIDKDLLSDLEEYEDEEVDGSKEKIKREERLQQKMNMTKNPLTSVSLTATKDEDH
IKRRVAMLCSALGGFDLNPPQDYILGIEAAGCLRDIKRLLKVVDEEKNVHVVASACHDSGLLINDLV
PIVVQFGTTDVANADPDIIKLLLSCLELITKLMTPITLGNDDVDDTGLLKLKRAQIEYKHRLLHYKKGKI
FKYLVALIIPVLQLERSELTHRDNIILNLCLTLFTNILRIKPSDANTAKKNKSHVINVFQELPPGILEEDIS
LEVVLEVYRKYKILPIVQTIASNLSNEFESRILGTACLDFYYYSFVSVDPKSLVDDIGKAGNALDPLPKS
AAIADARDETNDRNVSNINDRLRQLKMKEKENIRSFKSKSSTRHAKFGSLISVQDRKNGTRVLSGQ
DKLRDTNIVGLLDKNVSKIESGKIFNRINKKENEGHEKIVRLNIRSRELLSKFVQDFIENGFPIVFREM
YSMILEGLVHPEEKKIYKLDYDFFFVVNWILRFEMCYQSVHKRGLRTVSKYKYFYCCFTGKTIKDLIN
VFIPTYTKVNYDCLRVTTCVLKNILSSALTIHSYENFEDSKMLSDDKMVLKALVQTGEAVLRLVFEIES
EVKSLLRLPQDAHRRSYLFGLEMIEFTHVVLKVLSYISKLSIPIMLFRKYSDDFNEDDIYDGQDRSNLT
KNALNRYKELDRGRCEDFKDLLFHDNIVSTHIWLFKQYQEVEEEKLNLVLFYFRKLLLKWERNILKLF
RLDFMHALYEFKNADVSPKLKLDFGNLTNFFMHHFTKICQHSPYAFIDLAVFDETFDSDIKTYLMSG
DPMALGSLTFKERRKTERRGNKDVKFRDDLMPDKRKTILLVSLLFFKDMTAVLDTLSKYLQQWYDEL
TQTFDGKDPIGPLNVYRFAGTLQGEYRSNPYFRLLCNVGGIVNDFKTNVEQAMETPVDPDMLEDIL
DDPNIEIQQLHQYNKRRKRQNVDDVDDDPDLNDGYDSYLEDDSYGDEVVNDGDDLAGSGDEQ
QDELELMETRLQSLENRIKGKAMKRTVDGQLVEMGKKLRRKKSKKSKDKKKTHKRRRIVEDDNEIV
SDGEIKRRANLSKHMIYDSDEEANEDPNFFEREIKLHQLLQKRHGLITKEQYNSLMDGSLDVDDVS
DLDDNLVVDTEKDLEITAAASKPDRELLIDLLRGGKSLSNDEENVIMSGESSHESDTSDEDEISEEH
QVSVELGRIRDMLRNSSGEPEVFNNVVNKRPTKLRIVDDEEEEEEEEEEEEEKRKLAKPGGDNTNS
DVAANIHPEEQMQETEIDSQMTSTSSVDSTTQTTENTESDILNSLRQIHDVINLQNI

>tr|A0A0C9MJ45|A0A0C9MJ45_9FUNG Cysteine protease OS=Mucor ambiguus OX=91626 GN=MAM1_0161c06941 PE=3 SV=1

MTHHSNEQKELPQPHNSNHISTAPLKDKLLTNSSYLAAEIPLKLGHFMSNLWISGSELSNSFFLQH
GECNPDSENAQIIWLLGRPYQTKPLDQMQQAILDAQKDEMFRPGGAATDRDDEEVNKDNVSMV
WPPDFYDDFTSRLWMTYRHNYPPIRPSNHKTDIGWGCMLRSGQSLLANTLLIHFLSRDWRRQKQ
TAAARLQYAKIIHWFLDELSPRAPFSIHRIALLGKQLGKNIGEWFGPSTISQVIQALVSDFQPADLSVY
IATDGTIYLDGVQDVTAGKKPRGDFSYFTNKLSSSTTDEGREEAKHLYDASSTPQEQDQATYPSADS
PDEKDTVFKPVLMLVALRLGIDSLHPTYYPALKACFELPSFVGIAGGRPNSSLYFIGLQGDDLIYLDP
HFSRPALETKSLSEYTREDFSTYHCTIPRRIPIANLDPSMMLGFYCRTQRELDLFCDQIKTISQKHTAI
FSIQQSAPEYDEDVRSENDFGVMSDEDDPLESESKCKDDDSDGNSIF

>tr|A0A0D0TCC4|A0A0D0TCC4_9TREE Autophagy-related protein 4 OS=Cryptococcus deuterogattii Ram5 OX=1296110 GN=I313_00622 PE=3 SV=1

MSSPTPTSPTSSLAFSPTSFSHASIASTNVRSRTNLPPPPPPDRIPPPKGRPHQQKFKILRKGKDKDK
RQPMNSEDDWAIEDVVANGDSIEQEPQYLDDLQSEENESKPEQQLVEMASREEKKEKASKSRVLV
RKTSRLFGRDKDKDRDKFEEPAVTGSSSSTLAATRQSSSTSTDSSTSRSFTSAFTRMNSIQSKRSPRR
SFGQAHSRRASQDSQMSWPAPRSIHSSITSHDTSGDPQDGAGGNSVPIPQRQGASMSSLSPYSL
PQPNGNTSRSPDTFPNKMSTWFSHLLPVSSETPSPSTNETASSVRKQPSVAASLFNAARQKAVDG
VRHLLDSEAQPDKCMDTIWVRGVAHPGWRPITPENVASNLPALEPVDGGVEDRRASVSMNRPSP
TSFRPSSWRRNTSLPPSAQLQPSTQIHSQASNQTTSPNKGFTGIWNPSTLSLAMSIGGSPNKEKEN
GSGAESPSKKKGKEVVKWPEQFYDDFKSIVWFTYRNQYAPILSLSPDLLIPSPEAYYASFGPPLDATS
PSSPQVTTPTAIAQQTATGGGGWSWSKEERGLTSDAGWGCMLRTGQSLLVNALIHVHLGRDWRL
PSTPSIFSEATTSQEIAALKDYAKYAQMLSWFLDDPSPLCPFSVHRMALIGKELGKEVGEWFGPSTA
AGTLKTLANSFAPCGVAVATATDSIIYRSDVYAASNLPSDDWNRISPTFYPNRKKKRDNAEAKEGKW
GERAVLILVGIRLGLDGVNPIYYDSIKALFTFPQSVGIAGGRPSSSYYFVGSQANHLFYLDPHLTRPAI
PLQVPPLPVHSAKEEGSMESSSILSTAEEESEEGVMIHTPETPRSTTPSTFSAAEEVEDEEREEWEQG
SKYKLDVMDADGFRVEEGISDDKGRNEGTSIGSDVPKSTLESNGVIQERSDKQNFFTSTTSTDSQV
DPQMLWYTTAYPDSLLRTYHCEKIKKMPLSGLDPSMLLGFVCKSEDDFENFVERVAQLPKKIFTVQ
DEMPSWGEDDDAGLESVSEPDFEEDEFEEPGTAKPRFDSSSPVNEDSLKGPSIVSASTTATPIAAKE
DYLDGEDGDGTDDDDESIGTTTAVRPVDIVRHLHRVDLSVKREQEGGDDDDGEWVGGTPSSQG
VLVEPLSLKGTPSKSRSSTVEPHHEQSEEEQERRVFPVRNRMESWVEPVCEGKEAPNGDNLL

>tr|A0A0D0VN62|A0A0D0VN62_CRYGA Autophagy-related protein 4 OS=Cryptococcus bacillisporus CA1280 OX=1296109 GN=I312_02977 PE=3 SV=1

MSSPTPTSPTSSLFSPTSFSHASIASTNVRSRTNLPPPPPPDRIPPPKGRPHQQKFKILRKGKDKDKR
QPTNSEDDWTIEDVIVNGDGIEQGPQYLDDLQWEENESKPERQLVEMASREEKKGKASKSRELVR
KTSHLFGRDKDKDRDKFEEPAVTGSSSSTLAATRQSSSTSTDSSTSRSFTSAFTRMNSIQSKRSPRRS

FAQAHSRRASQDSQMSWPAPRSIHSSITSHDPSSDPQDGAGGNGVPIPQRQGASMSSLSRYSLP
QPNGNMSRSPDTFPNKMSTWFSHLLPVSSETPSPSTNETDSPVRKQPSVAASLFNAARQKAVDGV
RHLLDSEAQPDKCMDTIWVRGVAHPGWRPITPENGASNLPALESGDGGVEDRRASSSMNRSSPT
SFRPSSWRRNTSLPPSAQLQPSTQIHSQASNQTTSPNKGFTGIWNPSTLSLAMSIGGSPNKEKEIG
SGAESPSKKKGKEVVKWPEQFYDDFKSTVWFTYRNQYAPILSLSPDLLIPSPEAYYASFGPPLDATSP
SSPQVTTPTATAQQTVTGGGGWSWSKEERGLTSDAGWGCMLRTGQSLLINALIHVHLGRDWRLP
STPATFSEAATFQEIAALKDYAKYAQMLSWFLDDPSPLCPFSVHRMALIGKELGKEVGEWFGPSTAA
GTLKTLANSFAPCGIAVATATDSIIYRSDVYAASNLLYDDWNRISPTFNPSTKKKRDNAEAKEGKWGE
RAVLILVGIRLGLDGVNPIYYDSIKALFTFPQSVGIAGGRPSSSYYFVGSQANHLFYLDPHLTRPAIPL
QVPPLPVHSAKEEGSMESSSILSTAEEESEEGVMIHTPETPRSTTPSTFSAAEEVGDEEREEWEQGP
KYKLDVMDADGFRVEEEISDDKGRNEGKAIGSDIPKSALESNGVIRERSDKQKTFTSTTSVDSQVDP
QMLWYTTAYPDSLLRTYHCEKIKKMPLSGLDPSMLLGFVCKSEDDFENFVERVAQLPKKIFTVQDE
MPSWEEDDDAGLESVSEPDFEEDEFEEPGTAKPRFDSSSPVNEDSLKGPSIVSASTTATPIAAKEDY
LDGEDGDGTDDDDESIDTTTAVRPVDIVRHLHRVDLSVKREQEGGDDDDGEWVGGTPSSQGVLV
EPPSMKGTPSKSRSSTVEPHHEQSGEEEQERRVFPVRNRMESWVEPVCEGKEAPNGDNLL

>tr|A0A0F0IKI5|A0A0F0IKI5_ASPPU Cysteine protease OS=Aspergillus parasiticus (strain ATCC 56775 / NRRL 5862 / SRRC 143 / SU-1) OX=1403190 GN=P875_00109108 PE=3 SV=1

MNSVDIGRCRKRIVQYIWDPEPRNDEEPDASIWCLGVEYAPQLQKITANTTPDQDELEAGSSRIDD
VTAHGWPEAFVSDFESKIWMTYRSDFPPIPRPDNDEANHPMTLTVRIRTQLMDPQGFTSDTGWGC
MIRSGQSLLANAMLTLCLGRDWRRGDKAEEEARLLSLFADHPDAPLSIHRFVKYGAESCGKYPGE
WFGPSATARCIEYAPNASTIPRARDLPATGLSQHNLPPLGIDNVTPVYWDGLKAVLQLPQSVGIAGG
RPSASHYFIGTQGPHLFYLDPHTTRPALPYSIDGRLLSKTEISTYHTRRLRRIHIQDMDPSMLIGFLVK
DEDDWEDWKGRVGSVMGKQIIHVFKGAEATYNQGRRGALDEVEALDDAE

>tr|A0A0J6EUZ8|A0A0J6EUZ8_COCPO Cysteine protease OS=Coccidioides posadasii RMSCC 3488 OX=454284 GN=CPAG_00708 PE=3 SV=1

MNTVDIGQKYKRIVEYLWDPEPKNDDDIIEPVWCLGKEYKTSIPRDSEGAEAPESCNMPGMPFLSP
MNQMSLSSRDTQAALSKPATPPHQLGIQRSKSREWPTSFLDDFESKFWFTYRSNFPAIPKSRDPDT
PLALTLSVRLRSQFLDTHGFTADTGWGCMIRSGQSLLANALSILNLGRDWRRGSKIKEECELLSLFA
DNPQAPFSIHRFVDYGASACGKHPGEWFGPSATARCIEALSNECKHTDLNVYVMSDGSDVHEDQ
FRQIAGPDGIRPTLILLGVRLGIESVTPVYWEALRAIIRYPQSVGIAGGRPSSSLYFIGVQGPYFFYLDP
HHTRPAVSWNPDSTLSPENLDTYHTRRLRRLHIREMDPSMLIGFLIKDDDDWKDWKRRLRSVTGN
PIIHIFDLERPNFGRHLEREEAVDEVEALDDDSN

>tr|A0A0J6YRU6|A0A0J6YRU6_COCIT Cysteine protease OS=Coccidioides immitis RMSCC 2394 OX=404692 GN=CIRG_08931 PE=3 SV=1

MNTVDIGQKYKRIVEYLWDPEPKNDDDIIEPVWCLGKEYKTSIPRDSEGAEAPESCNMPGMPFLSP
MNQMSLSSRDTQAALSKPATPPHQLGIQRSKSREWPTSFLDDFESKFWFTYRSNFPAIPKSRDPDT
PLALTLSVRLRSQFLDTHGFTADTGWGCMIRSGQSLLANALSILNLGRDWRRGSKIKEECELLSLFA
DNPQAPFSIHRFVDYGASACGKHPGEWFGPSATARCIEALSNECKHTDLNVYVMSDGSDVHEDQ
FRQIAGPDGIRPTLILLGVRLGIESVTPVYWEALRAIIRYPQSVGIAGGRPSSSLYFIGVQGPYFFYLDP
HHTRPAVSWNPDSTLSPENLDTYHTRRLRRLHIREMDPSMLIGFLIKDDDDWKDWKRRLRSVTGN
PIIHIFDLERPNFGRHLEREEAVDEVEALDDDSN

>tr|A0A0J8QLI6|A0A0J8QLI6_COCIT Cysteine protease OS=Coccidioides immitis RMSCC 3703 OX=454286 GN=CISG_09869 PE=3 SV=1

MPGMPFLSPMNQMSLSSRDTQAALSKPATPPHQLGIQRSKSREWPTSFLDDFESKFWFTYRSNFP
AIPKSRDPDTPLALTLSVRLRSQFLDTHGFTADTGWGCMIRSGQSLLANALSILNLGRDWRRGSKIK
EECELLSLFADNPQAPFSIHRFVDYGASACGKHPGEWFGPSATARCIEALSNECKHTDLNVYVMS
DGSDVHEDQFRQIAGPDGIRPTLILLGVRLGIESVTPVYWEALRAIIRYPQSVGIAGGRPSSSLYFIGV
QGPYFFYLDPHHTRPAVSWNPDSTLSPENLDTYHTRRLRRLHIREMDPSMLIGFLIKDDDDWKDW
KRRLRSVTGNPIIHIFDLERPNFGRHLEREEAVDEVEALDDDSN

>tr|A0A0L0NN11|A0A0L0NN11_CANAR Cysteine protease OS=Candida auris OX=498019 GN=QG37_08318 PE=3 SV=1

MPPELSKEESSSSSPTSETAHSSSNSVQTSLDRAHVNEPKNAGSGTDSPSEAQNLFQSVLAQFWQ
RIRSPEATDQPQPLGLVVIMGSSYQGTLTLPHITEAIQRRIWFTYRTGFEPIPKAEDGPSPLSFIGSMIV
HGANAHGTISGLFDNSNFTSDVGWGCMIRTAQCLLANTLQTVLVKGAVKEGKMPDWNAVNSHVI
SLFKDTYKTPYSLHNFVRVASHLPLKVKPGQWFGPSAASLSIKRLSCAANQRGNISPELSVMICESC
DLHDDEVKHVFEQSGRPLLLLFPIRLGIETINSIYYPSLFQLLSLRNSMGIAGGKPSSSFYFFGYQDQ
LLLYLDPHTSQHIDQTNGSYKPATCSTVHISGLDPSMVVGLLVESYQDYEELKAKLKDANKIVHFHE
RAPARKSASVPVQDRSMEHVESERDSIEDLGDFVDLGDDIADPISEAEFGNDSFDPSSSFVAKYDV
VDGPSPVTVLDDEEEA

>tr|A0A168MY55|A0A168MY55_MUCCL Cysteine protease OS=Mucor lusitanicus CBS 277.49 OX=747725 GN=MUCCIDRAFT_91441 PE=3 SV=1

MTHHSNEQKHVDNELPQPHNSNHISTAPLKDKLLTNSSYLAAEIPLKLGHFMSNLWISGSELSSSF
FLQNGDCNIDSENAQIIWLLGRPYQTKPLDQMQQAILDAQKDEMFRPGGTAADRDDEEVHKDNV
SMVWPPDFYDDFTSRLWMTYRHNYPPIRPSNHKTDIGWGCMLRSGQSLLANTLLIHFLSRDWRR
QKQTAAARLQYAKIIHWFLDELSPRAPFSIHRIALLGKQLGKNIGEWFGPSTISQVIQALVSDFQPAD
LSVYIATDGTIYLDSVQDVTTGKKPRGDFSYFTDKLSCSTSDEGREEAKHLYDASSTPQEQEQATSPN
ADSPNDTDTVFKPVLMLVALRLGIDSLHPTYYPALKACFELPSFVGIAGGRPNSSLYFIGLQGDDLIYL
DPHFSRPALETKSLSEYTREDFSTYHCTIPRRIPIANLDPSMMLGFYCRTQRELDLFCDQIKTISQKHT
AIFSIQQSAPEYDEDVRSENDFGVMSDEDDPLDNESKCKVDNDDDGNSDSDSNSIF

>tr|A0A1D9QGW5|A0A1D9QGW5_SCLS1 Cysteine protease OS=Sclerotinia sclerotiorum (strain ATCC 18683 / 1980 / Ss-1) OX=665079 GN=sscle_12g089570 PE=3 SV=1

MTTVDIGRYKRLVQYFWDPEPTNNIASKSPIWCLGEEYLVSDKSSPSAVTESPPKEGGYLLAQSLSTT
ETTTPPDSTVGSLESSSEYDNCDTASTDGGWPTAFLDDFEAKIWLTYRSNFPAIAKSQDPKALSAMS
LSVRLRSQLVDQGGFTSDTGWGCMIRSGQSLLANALLTLRMGREWRRGSSSNEERKILSLFADDP
RAPYSIHKFVEHGASACGKHPGEWFGPSAAARCIQALTNSQVESELRVYITGDGSDVYEDTFMSIA
KPNSTKFTPTLILVGTRLGLDKITPVYWEALKSSLQMPQSVGIAGGRPSSSHYFIGVQESDFFYLDPH
QTRPALPFNDNVEDYTPEDIDSCHTRRLRRLHIKEMDPSMLIAFLIRDENDWKDWRRAVREVQGK
GVIHVADRDPALHGLGAERDGAIDEVETFDDDDDDTVLNG

>tr|A0A1E3HI40|A0A1E3HI40_9TREE Autophagy-related protein 4 OS=Cryptococcus amylolentus CBS 6039 OX=1295533 GN=L202_05976 PE=3 SV=1

MPSPSTHAPRASISSNLPSPPPADRVPTPKGRTHQFKLLRKGKDRERRHPTDLEDDWTIEGMSPDA
VEDEEFLEDSEPALEPEPIVPEMPVREEAKKPSSKGRQLVKKTSRLFSRDKDREGDSSSFAPSSSSSL
VASRQTSASSADSQTTGSASRTLPSAFIQRHSSMQSKSRSPRTSFGQPSHSRRASQDSQSSWQAP
RSIRSFASNDTEPSAPRGLPIPQRQGASIPSLSRQSLPQPINVNSRSPETIPGRMSSWFSHLLPTAE
NQPASLGSSPGAEATSPIRKQPSVAASLFNAARQKAVDGVRHLLDSEAQPDKCMDTIWMRGVGH
PGYRPKTPDTTSSPLPSVEGVQEEGRRSSLSMNRPSPTGLRPSSWRRNASISQGIPAQTNQSTPPN
KGFTNLFNSSSLSLAMPMGSSPSKEKEGPDSPNKKKPGKEVLQWPEQFYDDFRGTVWCTYRNQY
APILALSPNLLIPSPEAYYASFAPPLDVASAPPAPPSPQQTPTQNSSFQAQQAPTGWWGKEERGLTS
DAGWGCMLRTGQSLLVNAIVHIHLGRDWRAVRPPTPPTSAAEAEVLEQYAKYIQIISWFLDDPSPL
CPFSVHRMALIGKELGKEVGEWFGPSTAAGALKTLANSFAPCGVSVATATDSIIYKSDVYAASNLSS
DAWDQLNPAFSIGRPNQTDRGNRSGPGQKWGHKAVLVLVGVRLGLDGVNPIYHDSIKTLFTFPQS
VGIAGGRPSSSYYFIGTQANSLFYLDPHLTRPAVPLQVPPLPTLPTEEEVVVIPSPQRQPHTHSRSHS
RSPYRSPSRSRSRPPGSPTKASPLQNSLDISPEVAKYKLDVVNVDDVSESDSEGEGQRVKSPKKEAI
RRGFTSPTGTPSKNGVKAMNDDVGSPRKRTPSASPTKGSINYSYPLSANSSQPASESSTPAREVPE

MTARPKVDPHAEWYIQAYPEHLMRTYQCEKVKKLPISGLDPSMLLGFVVRDEKDFEDFIDRVNKLP
KKIFTIQNEQPQWEEEDDVGLESVSEPELDSEDEFDAPGTAKPRFEAGSPIDSDMMSGPNVLAASE
LDMDKIPDIPSTTATPQVAREDYVGMSGSASASGSGTGPTASSSTSAPTTLDEDNGDGSLLDGSME
TTVGAVDIAKHLQRVDIANSDGEEEDGELVGGTPSSGGVMVEPPSVHNTPNRVRPVADRQDTARP
VSSSPEEIPRHVRKGAESGHEDEKEVDSKATSGTEMPPMRNRMESWVEPIRGGQEAPNGDSLL

>tr|A0A1E3IVK6|A0A1E3IVK6_9TREE Autophagy-related protein 4 OS=Cryptococcus depauperatus CBS 7841 OX=1295531 GN=L203_106188 PE=3 SV=1

MASATSPPSTRNLPSPPPPDRLPPPKGRPRQKFKILGKQKDRGRRDVEPELDDDWTLEGNTETHV
QEHAGDSVERNSMDVRRAIDQDRDTQVYEIAIKEEEKKEKRSKGRQLVKKTSRLFGRDKDKSESNS
ANTNGSSAILPLRQISSVSGETQATASRHLPSAFLNRQASLQTKSRPSRDSFSQQRYPRRTSHDSQ
VSWQASRSILSYTSQDTMVESSPTNGLGLPIPQRQGASIPTLSRLSLPQPAGSSNSLRSPETFPAKM
STWFSHLLSTTESSAGLSETIISPIRKQPSVASSLFNAARQKAVEGVRHLLDSEATPDKCMETIWVRG
VDHLGWRPVTPENGPLALPVEYPENRRSSVSSNRPSPTVFRPSSWRRSNAPHPAHVFPPQPNSP
AAGSCPNTSITNLFNGSALSLALPGGSPSMDREKYDGGDSPSKKKGKEVVRWPEQFYDDFKSTIW
CTYRTQYAPILNLSPNLLIPTPEAYYASFGPPLDATVLQPLALQQQASSPTQTTPVTTTYSTGWWNRE
ERGLTSDAGWGCMLRTGQSLLVNALIHVHLGRNWRLPATYPPNPPTSISEVEATKAYAKYTQILSWF
FDDPSPLCPFSVHRMALIGKELGKEVGEWFGPSTAAGALKTLANSFAPSGVSVATATDSIIYKSDVYA
ASRISSESWRQAHPCFAPFGGDKDVAYGGKELRADGSGDKWGERAVLVLVGVRLGLDEVNPIYYD
SIKALFTFPQSVGIAGGRPSSSYYFIGTQANSLFYLDPHTTRPAIPLQIPPLPSYPTVICTSTSEESDSSS
RGSLDKTTFDSASTSTDEGVMIPSSKTPSTPSSTFTQPAPSQEAEIVQYKLDVVDMDDVSEDSDEDG
VHIDRFVSTDEHTPGRGGRMVKKSPSSEKMDYESPTQRSPETIPAVANPTPTLPSHTSADPVNIQID
PYTLWYATAYSPSFLRTFHCEKVKKMPYSSLDPSMLLGFICRNESDFVDFVERIGKLPNKIFTVQDEQ
PVWAEDDDVGLESVSEPDFDEDDEGEAGEGEPRAI

>tr|A0A1E3J8F5|A0A1E3J8F5_9TREE Autophagy-related protein 4 OS=Cryptococcus amylolentus CBS 6273 OX=1296118 GN=I350_08114 PE=3 SV=1

MPSPSTHAPRASISSNLPSPPPADRVPTPKGRTHQFKLLRKGKDRERRHPTDLEDDWTIEGMSPDA
VEDKAFLEDSEPALEPEPIVPEMPVREEAKKPSSKGRQLVKKTSRLFSRDKDREGDSSSFAPSSSSS
LVASRQTSASSADSQTTGSASRTLPSAFIQRHSSMQSKSRSPRTSFGQPSHSRRASQDSQSSWQA
PRSIRSFASNDTEPSAPRGLPIPQRQGASIPSLSRQSLLQPINVNSRSPETIPGRMSSWFSHLLPTAE
NQPASLGSSPGAEATSPIRKQPSVAASLFNAARQKAVDGVRHLLDSEAQPDKCMDTIWMRGVGH
PGYRPKTPDTTSSPLPSVEGVQEEGRRSSLSMNRPSPTGLRPSSWRRNASISQGIPAQTNQSTPPN
KGFTNLFNSSSLSLAMPMGSSPSKEKEGPDSPNKKKPGKEVLQWPEQFYDDFRGTVWCTYRNQY
APILALSPNLLIPSPEAYYASFAPPLDVASAPPAPPSPQQTPTQNSSFQAQQAPTGWWGKEERGLTS

DAGWGCMLRTGQSLLVNAIVHIHLGRDWRAVRPPTPPTSAAEAEALEQYAKYIQIISWFLDDPSPL
CPFSVHRMALIGKELGKEVGEWFGPSTAAGALKTLANSFAPCGVSVATATDSIIYKSDVYAASNLSS
DAWDQLNPAFSIGRPNQTDRGNRSGPGQKWGHKAVLVLVGVRLGLDGVNPIYHDSIKTLFTFPQS
VGIAGGRPSSSYYFIGTQANSLFYLDPHLTRPAVPLQVPPLPTLPTEEEVVVIPSPQRQPHTHSRSHS
RSPYRSPSRSRSRPPGSPTKASPLQNSLDISPEVAKYKLDVVNVDDVSESDSEGEGQRVKSPKKEAI
RRGFTSPTGTPSKNGVKAMNDDVGSPRKRTPSASPTKGSTNYSYPLSANSSQSASESSTPAREVPE
MTARPKVDPHAEWYIQAYPEHLMRTYQCEKVKKLPISGLDPSMLLGFVVRDEKDFEDFIDRVNKLP
KKIFTIQNEQPQWEEEDDVGLESVSEPELDSEDEFDAPGTAKPRFEAGSPIDSDMMSGPNVLAASE
LDMDKIPDIPSTTATPQVAREDYVGMSGSASASGSGTGPTASSSTSAPTTLDEDNGDGSLLDGSME
TTVGAVDIAKHLQRVDIANSDGEEEDGELVGGTPSSGGVMVEPPSVHNTPNRVRPVADRQDTARP
VSSSPEEIPRHVRKGAESGHEDEKEVDSKATSGTEMPPMRNRMESWVEPIRGGQEAPNGDSLL

>tr|A0A1E3JMA8|A0A1E3JMA8_9TREE Autophagy-related protein 4 OS=Cryptococcus wingfieldii CBS 7118 OX=1295528 GN=L198_02740 PE=3 SV=1

MPSPTPSPRPSPSSNLPAPPPADRAPPPKGRTHQFTLLRKGRDRQKRLPTDLEDDWTIEGMSPHP
VQHNELLDDPQPAPEPEPIVPEIPVREEAKKPPSKGRQLVKKTSRLFSRDKDREGDSSLAPSSSSSL
VASRQTSASSADSQPTGSASRTRPSAFIQRHSSMQSKSRSPRTSFGQPTHSRRASQDSQSSWQAP
RSIRSFASTDTEPSAPRGLPIPQRQGASIPSLSRQSLPQPINVNSRSPETIPGRMSSWFSHLLPTAEN
QPASLGSSPGAEATSPIRKQPSVAASLFNVARQKAVDGVRHLLDSEAQPDKCMDTIWMRGVGHP
GYRPKTPDTTSSPLPSVEGVQEEGRRSSLSMNRPSPTGLRPSSWRRNASISQGIPTQANQSTPPNK
GFTNLFNSSSLSLAMPIGSSPSKEKEGPDSPNKKKPGKEVLQWPEQFYDDFRGTVWCTYRNQYSP
ILALSPNLLIPSPEAYYASFSSPLDVASPPPASPSPQQTPTQNSSFQAQQAPGGWWGKEERGLTSD
AGWGCMLRTGQSLLVNAIVHIHLGRDWRAIRPPTPPTSAAEAEALEQYAKYIQIISWFLDDPSPLCP
FSVHRMALIGKELGKEVGEWFGPSTAAGALKTLANSFAPCGVSVATATDSIIYKSDVYAASNLSSDA
WDQLIPAFSIGRPNQTDRGNKSGSGQKWGHKAVLVLVGVRLGLDGVNPIYHDSIKTLFTFPQSVGI
AGGRPSSSYYFIGTQANSLFYLDPHLTRPAVPLQVPPLPTLPTEEEVVGIPSPQRQPYTPSRSRSRPQ
ESPTKASPLRNSLDINPEVAKYKLDVVNVDDVSESDSGGEGERVRSPKKEAIRRGVMSPTGTPSKN
GVKGMKDDVGSPHKRTPSASPTKGSINYSYPLSANSSQPASRSSTPAREVPEMTARPKLDPHAEWY
IEAYPEHLMRTFQCEKVKKLPISGLDPSMLLGFVVRDEKDFEDFVDRVNKLPKKIFTIQNEQPQWEE
EDDVGLESVSEPELESEDDFDAPGTAKPRFEAGSPIDSDMMSGPSVLAASELDMDKIPDIPSATATP
QVAPEDYVGMSGSTSASGSGTGPTASSSTSAPTTLDEDDGDGSLLDGSMETTVGAVDIAKHLQRV
DIANNDGEEEDGELVGGTPSSGGVMVEPPSVHSTPNRVRPVADRQDTARPVSSSPEDIPRHVRQG
AESGQEEEEVDSKATRGTELPTMRNRMESWVEPIRGGQEAPNGDNLL

>tr|A0A1F7ZJY8|A0A1F7ZJY8_9EURO Cysteine protease OS=Aspergillus bombycis OX=109264 GN=ABOM_011022 PE=3 SV=1

MNSVDIGRCSKRIVQYLWDPEPRNDQEPDASIWCLGVEYAPRLQKHTANTASDRDELDVGTNTID
DVTAHGWPEAFISDFESKIWMTYRSNFPPIPRPDNDEANHPMTLTVRLRTQLMDPQGFTSDTGWG
CMIRSGQSLLANAMLILCLGRAWRRGDKAEEEAHLLSLFADHPDAPLSIHRFVRHGAESCGKHPG
EWFGPSATARCIEALSAQCGNIAPRVYVTNGTSDVYEDSFLRVARSGSGSIQPTLILLGTRLGIDNVT
PVYWEGLKAVLQLPQSVGIAGGRPSASHYFIGTQGSHLFYLDPHTTRPALAYSIGGRLPSKEEISTYH
SRRLRRIHIQDMDPSMLIGFLVRNEDDWEDLKGRIGSIMGKPIIHVFEGAETTYNQGRREALDEVEA
LDDAE

>tr|A0A1L0D0D4|A0A1L0D0D4_9ASCO Cysteine protease OS=[Candida] intermedia OX=45354 GN=SAMEA4029010_CIC11G00000002609 PE=3 SV=1

MSGATPEGYVPPIEAIYQLLDTFGDLWKRIRNLDTDDNEVADVIVLGSVYPNAKLLSAEIASAIHSRL
WFTYRAGFEPIERAESGPGPLTFARSMIFNASPNNTIGGLFNNLTFLSDVGWGCMIRTSQSLLANAL
QRVVLGRDYQHEIPSAELDEILALFADDYKSPFSLHNFIQAASRLPLQVKPGEWFGPSAASLSIKRLS
DSLTVTNLPKLNVYIGENGGMCDKDIEAEFAKESAPLLVLLPIRLGINNINAIYHASLLQLLALRQSVG
IAGGKPSSSYYFFGYQDLDLLYLDPHHLQPVSEDYETYHTSRCLTLPISQLDPSMLVGLLFENLDDY
HSFKLEVHHTNKIVQFHDHTLRKKSFVDRGDDYVKVRRETDTVNIDDFVDIGDEFSDEEIGYDLSD
EDIVDCGALSVA

>tr|A0A1L0G9H4|A0A1L0G9H4_9ASCO Cysteine protease OS=[Candida] intermedia OX=45354 GN=SAMEA4029009_CIC11G00000000488 PE=3 SV=1

MSGATPEGYVPPIEAMYQLLDTFGDLWKRIRNLDTDDNEVADVIVLGSVYPNAKLLSAEITSAIHSRL
WFTYRAGFEPIERAESGPGPLTFARSMIFNASPNNTIGSLFNNLTFLSDVGWGCMIRTSQSLLANAL
QRIVLGRDYQHETPSAELDEILALFADDYKSPFSLHNFIQAASRLPLQVKPGEWFGPSAASLSIKRLS
DALTVTNLPKLNVYIGENGGMCDKDIEAEFAKASAPLLVLLPIRLGINNINAIYHASLLQLLALKQSVG
IAGGKPSSSYYFFGYQDLNLLYLDPHHLQPVSEDYETYHTSRCLTLPISQLDPSMLVGLLFENLDDY
HSFKLEVHHTNKIVQFHDYTLRKKSFVDRGDDYVKVRRETDTANIDDFVDIGDEFSDEEVGYELSDE
DIVDCGASSVA

>tr|A0A1S9DIK5|A0A1S9DIK5_ASPOZ Cysteine protease OS=Aspergillus oryzae OX=5062 GN=OAory_01101970 PE=3 SV=1

MNSVDIGRCRKRIVQYIWDPEPRNDEEPDASIWCLGVEYAPQPQKITANTTPDQDELEAGTSKIDD
VTAHGWPEAFVSDFESKIWMTYRSDFPPIPRLDNDEANHPMTLTVRIRTQLMDPQGFTSDTGWGC
MIRSGQSLLANAMLTLCLGRDWRRGDKAEEEARLLSLFADHPDAPLSIHRFVKYGAESCGKHPGE
WFGPSATARCIEYAPNTSTPPRARDLPANRALSAQCGNIAPRVYVTNDTSDVYEDSFLRVARSGSG
SIQPTLILLGTRLGIDNVTPVYWDGLKAVLQLPQSVGIAGGRPSASHYFIGTQGPYFFYLDPHTTRPA
VPYSIDGRLLSKTEISTYHTRRLRRIHIQDMDPSMLIGFLVRNEDDWEDWKGRVGSVVGKQIIHVFK
GEEATYNQGRRGALDEVEALDDAE

>tr|A0A1V2LHB5|A0A1V2LHB5_PICKU Autophagy-related protein 4 OS=Pichia kudriavzevii OX=4909 GN=BOH78_4334 PE=3 SV=1

MGSQDIADGNDVYFSGLQNMWDRIWEFPGGLDKCSSESDLVILGMHYKVNDENQEDEKSEEQS
GISKCAGRATYEKIENSENSEDQLINVNYDKILDDKNTGDEPEKFDNNAIDNIKMFMKKISDTTYQKF
HSELNYTYPQNALDDLQTRLWFTYRYGFPLIKYDENGSPPIHLGAILRGNMDIQNFGKGFTSDSGW
GCMIRTSQTLLANALVSLKLGRNWRLQGSAPEDMKVHWEIVEQFADSPDARFSIHNMVLYAAKYC
GRRPGEWFGPSNAARSIEMLCNESQVDTNLKVYISSDSGDIYEEDLLNSCYEKNIDKFTPVLILCGV
RLGVHNINKIYWEFLKLTLDLPYSVGIAGGRPSSSHYFLGCQADYLLYLDPHIPQQAILLNDLKKAGS
SDYKSKMLGTLHTTKIKLLSLGKVDPSMLIGFLVKTREEYNDLRQKINLFPSNRRFLNFSNSKPVIRRT
SSVEDDIDGFVDLTMESGDEFLEHGEIIDDIDNKIKDMAEDKHQELAEYEKLEHAEIKDEPIVEIEKID
TPVKLQSERSGKYNNEIFASDGIIEVEDINNENSRDISGSRLERLKQLAMSTIEDDVFYEQDELDFEE
LIEKNMDSNFEDDENIDKDLLSDLEEYEDEEVDGSKEKIKREERLQQKMNMTKNPLTSVSLTATKDE
DHIKRRVAMLCSALGGFDLNPPQDYILGIEAAGCLRDIKRLLKVVDEEKNVHVVASACHDSGLLIN
DLVPIVVQFGTTDVANADPDIIKLLLSCLELITKLMTPITLGNDDVDDTGLLKLKRAQIEYKHRLLHYKK
GKIFKYLVALIIPVLQLERSEVTHRDNIILNLCLTLFTNILRIKPSDANTAKKNKSHVINVFQELPPGILEE
DISLEVVLEVYRKYKILPIVQTIASNLSNEFESRILGTACLDFYYYSFVSVDPKSLVDDIGKAGNVLDSL
PKSAAIADARDETNDRNVSNINDRLRQLKMKEKENIRSFKSKSSTRHAKFGSLISVQDRKNGTRVLS
GQDKLRDTNIVGLLDKNVSKIESGKIFNRINKKENEGHEKIVRLNIRSRELLSKFVQDFIENGFPIVFR
EMYSMILEGLVHPEEKKIYKLDYDFFFVVNWILRFEMCYQSVHKRGLRTVSKYKYFYCCFTGKTIKDL
INVFIPTYTKVNYDCLRVTTCVLKNILSSALTIHSYENFEDSKMLSDDKMVLKALVQTGEAVLRLVFEIE
SEVKSLLRLPQDAHRRSYLFGLEMIEFTHVVLKVLSYISKLSIPIMLFRKYSDDFNEDDIYDGQDRSN
LTKNALNRYKELDRGRCEDFKDLLFHDNIVSTHIWLFKQYQEVEEEKLNLVLFYFRKLLLKWERNILK
LFRLDFMHALYEFKNADVSPKLKLDFGNLTNFFMHHFTKICQHSPYAFIDLAVFDETFDSDIKTYLM
SGDPMALGSLTFKERRKTERRGNKDVKFRDDLMPDKRKTILLVSLLFFKDMTAVLDTLSKYLQQWY
DELTQTFDGKDPIGPLNVYRFAGTLQGEYRSNPYFRLLCNVGGIVNGAFISKSAQKLLDFKTNVEQA
METPVDPDMLEDILDDPNIEIQQLHQYNKRRKRQNVDDVDDDPDLNDGYDSYLEDDSYGDEVV
NDGDDLAGSGDEQQDELELMETRLQSLENRIKGKAMKRTVDGQLVEMGKKLRRKKSKKSKDKKK
THKRRRIVEDDNEIVSDGEIKRRANLSKHMIYDSDEEANEDPNFFEREIKLHQLLQKRHGLITKEQY

NSLMDGSLDVDDVSDLDDNLVVDTEKDLEITAAASKPDRELLIDLLRGGKSLSNDEENVIMSGESS
HESDTSDEDEISEEHQVSVELGRIRDMLRNSSGEPEVFNNVENKRPTKLRIVDDEEEEEEEEEEEK
RKLAKVKDQKNENLRLAGKPGGDNTNSDVAANIHPEEQMQETEIDSQMTSTSSVDSTTQTTENTE
SDILNSLRQIHDVINLQNI

>tr|A0A225ZI91|A0A225ZI91_CRYNV Autophagy-related protein 4 OS=Cryptococcus neoformans var. grubii c45 OX=1230068 GN=C356_01887 PE=3 SV=1

MSSPASTSPKSSFTFSPTSFPHASIASTNVRPRTNLPPPPPDRIPPPKGRSHQQKFKTLRKGKDKDK
RQPTDLEDDWTIEDVTVNNGSVEQEPQYLDDVQPEENELKPEPRLVEMANREEKKEKTFKSRELVK
KTSRLFGRDKDKDRDKSEEPAGSSSSTLAAMRQSSSTSTDSTTSRSITSAFTRQNSIQSRRSPRTSFG
QAHSRRASQDSQMSWPTPRSIRSSMTSHDPSNDPQNSANNTGVPIPQRQGASMSSLSRYSLPQ
PNGNASRSPDTFPNKMSTWFSHLLPVSSESPPSSNYETSSSVRKQPSVAASLFNAARQKAVDGVR
HLLDSEAQPDKCMDTIWVRGVAHPGWRPITPENGTSNLPTLEPGGGGGVEDKRASLSMNRPSPT
SLRPSSWKRNTALPPTAQPQPSTQQHTQASNQTTSPSKGLTGIWNSSTLSLGMPIGGSPNKERESG
SGAESPSKKKNKEIVKWPEQFYDDFKSTVWCTYRSQYAPISSLSPNLLIPSPEAYYASFGPPLDATSP
SSPRVTIPTAAAQQTASGSGGWGWSKEERGLTSDAGWGCMLRTGQSLLVNALIHVHLGRDWRVP
STAASFPEATTNQEIAALKDYAKYAQMLSWFLDDPSPLCPFSVHRMALIGKELGKEVGEWFGPSTA
AGALKTLANSFAPCGVAVATATDSIIYKSDVYTASNLPSDDWNSISPTFNSSKKKRGGDNEAKEGKW
GKRAVLILVGIRLGLDGVNPIYYDSIKALFTFPQSVGIAGGRPSSSYYFVGSQANHLFYLDPHLTRPAI
PLQVPPLPVHSAKEEGSMENSSILSTTEEESEEGVMICTPETPRSTTPSTFSAPEQIEDEGQEEWGQ
GSKYKLDVVDADGAKVEEIDDGKGRNEGETIKEKIPKNAFESKGVVQDQSEKQRGSASTASVDSQV
DPQMLWYTTAYPDSLLRTYHCEKIKKMPLSGLDPSMLLGFVCKDEDDFEDFVDRVAQLPKKIFTVQ
DEMPSWEEDDDAGLESVSEPDFEGDEFEEPGTAKPRFDSSSPVNEDSLKGPRVVSASTTATPLAAK
EGHLDVEETNSTDDDDESSGTTTAVRPVDIARHLHRVDLSSKREQEGDDDDGEWVGGTPSSQGV
LVEPPSLKGTPSKSRSSALEPRHEQNGEKEQERHVFPARNRMESWVEPVCEGKEAPNGDNLL

>tr|A0A2G7FSB4|A0A2G7FSB4_9EURO Cysteine protease OS=Aspergillus arachidicola OX=656916 GN=AARAC_009150 PE=3 SV=1

MNSVDIGRCRKRIVQYIWDPEPRNDEEPDASIWCLGVEYAPQHQKITANTTPDRDELEAGPSRIDD
VTAHGWPEAFVSDFESKIWMTYRSDFPPIPRLDNDEANHPMTLTVRIRTQLMDSQGFTSDTGWGC
MIRSGQSLLANAMLTLCLGRDWRHGDKAEEEARLLSLFADHPDAPLSIHRFVKYGAESCGKYPGE
WFGPSATARCIEALSAQCGNIAPRVYVTNDTSDVYEDSFLRVARRGSGSIQPTLILLGTRLGIDNVTP
VYWDGLKAVLQLPQSVGIAGGRPSASHYFIGTQGPHLFYLDPHTTRPALPYSIDGRLLSKTDISTYHT
RRLRRIHIQDMDPSMLIGFLVKDEDDWEDWKGRVGSVMGKQIIHVFKGAEATYNQGRRGALDEV
EALDDAE

>tr|A0A2I1C1N3|A0A2I1C1N3_ASPN1 Cysteine protease OS=Aspergillus novofumigatus (strain IBT 16806) OX=1392255 GN=P174DRAFT_374133 PE=3 SV=1

MNGVDMERCKRIIQYIWDPAPRNDQPGDPIWCLGTKYTTDGMERDNRRSEGPNQLGPKATTPGQ
LDEQGWPESFLHDFESRIWITYRSNFPPIPKPINQDAFSAMTLSVRLRSQLVDQHG

FTSDTGWGCMIRSGQSLLANAISILLFGRGWRRGIDTDREAQLLLQFADHPDAPFSIHRFVQHGAE
SCNKHPGEWFGPSATARCIQALVSQHGSLNLGVYMTDDTADVHEDKFLDAAHDERGSFRPTLILI
GTRLGIDRITPVYWDAVKTTLQLPQSVGIAGGRPSASHYFVGVQGSHLFYLDPHQTRPALPRRNIDE
PYTDEEIETYHTRRLRRIHVRDMDPSMLIGFIIKDREDWAHWKSGVSVQGKPIVHVLSESDTAVFQG
REGAIDEVEVLDDD

>tr|A0A2P2HRD8|A0A2P2HRD8_ASPFA Cysteine protease OS=Aspergillus flavus (strain ATCC MYA-384 / AF70) OX=1392242 GN=AFLA70_92g002991 PE=3 SV=1

MNSVDIGRCRKRIVQYIWDPEPRNDEEPDASIWCLGVEYAPQPQKITANTTPDQDELEAGTSKIDD
VTAHGWPEAFVSDFESKIWMTYRSDFPPIPRLDNDEANHPMTLTVRIRTQLMDPQGFTSDTGWGC
MIRSGQSLLANAMLTLCLGRDWRRGDKAEEEARLLSLFADHPDAPLSIHRFVKYGAESCGKHPGE
WFGPSATARCIEYAPNTSTPPRARDLPANRALSAQCGNIAPRVYVTNDTSDVYEDSFLRVARSGSG
SIQPTLILLGTRLGIDNVTPVYWDGLKAVLQLPQSVGIAGGRPSASHYFIGTQGPHFFYLDPHTTRPA
VPYSIDGRLLSKTEISTYHTRRLRRIHIQDMDPSMLIGFLVRNEDDWEDWKGRVGSVVGKQIIHVFK
GEEATYNQGRRGALDEVEALDDAE

>tr|A0A319AYT4|A0A319AYT4_ASPLB Cysteine protease OS=Aspergillus lacticoffeatus (strain CBS 101883) OX=1450533 GN=BO96DRAFT_470355 PE=3 SV=1

MNTVDIGRCSKRIVQYLWDPEPRNDEDPNSSIWCLGIEYHPDKDANTRETPDKNNTRENVMGTT
NYRKPSEHAWPESFLLDFESRIWMTYRSNFPPIPRVEGDDKSASMTLGVRLRSQLVDTQGFTSDTG
WGCMIRSGQSLLANALSMLVLGRDWRRGARFEEESQLLSLFADTPTAPFSVHRFVKHGAESCGKY
PGEWFGPSATAKCIEALSSQCGNPTLKVYVSNDTSEVYQDKFMDIARNTSGAFQPTLILLGTRLGID
NITPVYWDGLKAALQFPQSVGIAGGRPSASHYFVGAQGSHLFYLDPHYTRPALPDRQEGELYSKEE
VDTYHTRRLRRIHVRDMDPSMLIGFLIRNQEDWADWLKRIEAVKGRPIIHVLKQMNPDHDQEAGA
LDQVEALDDIE

>tr|A0A370C709|A0A370C709_ASPNG Cysteine protease OS=Aspergillus niger ATCC 13496 OX=1353008 GN=M747DRAFT_254677 PE=3 SV=1

MNTVDIGRCSKRIVQYLWDPEPRNDEDPNSSIWCLGIEYHPDKDANTRETPDKNNTRENVMGTT
NYRKPSEHAWPESFLLDFESRIWMTYRSNFPPIPRVEGDDKSASMTLGVRLRSQLVDTQGFTSDTG
WGCMIRSGQSLLANALSMLVLGRDWRRGARFEEESQLLSLFADTPTAPFSVHRFVKHGAESCGKY
PGEWFGPSATAKCIEALSSQCGNPTLKVYVSNDTSEVYQDKFMDIARNTSGAFQPTLILLGTRLGID
NITPVYWDGLKAALQFPQSVGIAGGRPSASHYFVGAQGSHLFYLDPHYTRPALPDRQEGELYSKEE
VDTYHTRRLRRIHVRDMDPSMLIGFLIRNQED

WADWLKRIEAVKGRPIIHVLKQMNPDHDQEAGALDQVEALDDIE

>tr|A0A370P3K7|A0A370P3K7_ASPPH Cysteine protease OS=Aspergillus phoenicis ATCC 13157 OX=1353007 GN=M752DRAFT_226353 PE=3 SV=1

MNTVDIGRCSKRIVQYLWDPEPRNDEDPNSSIWCLGIEYHPDKDANTRETPDKNNTRENVMGTT
NYRKPSEHAWPESFLLDFESRIWMTYRSNFPPIPRVEGDDKSASMTLGVRLRSQLVDTQGFTSDTG
WGCMIRSGQSLLANALSMLVLGRDWRRGARFEEESQLLSLFADTPTAPFSVHRFVKHGAESCGKY
PGEWFGPSATAKCIEALSSQCGNPTLKVYVSNDTSEVYQDKFMDIARNTSGAFQPTLILLGTRLGID
NITPVYWDGLKAALQFPQSVGIAGGRPSASHYFVGAQGSHLFYLDPHYTRPALPDRQEGELYSKEE
VDTYHTRRLRRIHVRDMDPSMLIGFLIRNQEDWADWLKRIEAVKGRPIIHVLKQMNPDHDQEAGA
LDQVEALDDIE

>tr|A0A3F3PL18|A0A3F3PL18_9EURO Cysteine protease OS=Aspergillus welwitschiae OX=1341132 GN=BDQ94DRAFT_110831 PE=3 SV=1

MNTVDIGRCSKRIVQYLWDPEPRNDEDPNSSIWCLGIEYHPDKDANPRETPDKNNTRENAMGTT
NYRKPSEHAWPESFLLDFESRIWMTYRSNFPPIPRVEGDDKSASMTLGVRLRSQLVDTQGFTSDTG
WGCMIRSGQSLLANALSMLVLGRDWRRGARFEEESQLLSLFADTPTAPFSVHRFVKHGAESCGKY
PGEWFGPSATAKCIEALSSQCGNPTLKVYVSNDTSEVYQDKFMDIARNTSGAFQPTLILLGTRLGID
NITPVYGDGLKAALQFPQSVGIAGGRPSASHYFVGAQGSHLFYLDPHYTRPALPDRQEGELYSKEE
VDTYHTRRLRRIHVRDMDPSMLIGFLIRNQEDWADWLNRIEAVKGRPIIHVLKQMNPDHDQEAGA
LDQVEALDDIE

>tr|A0A3M2T0S3|A0A3M2T0S3_9EURO Cysteine protease OS=Aspergillus sp. HF37 OX=1960876 GN=PHISP_05707 PE=3 SV=1

MNSVDLGRCKRFVQYLLDPEPKNDEEPDASIWCLGREYDPNDSTVISRDQPKPPTSNESEPSGET
AESSSAHGWPESFLTDFESRIWITYRSNFPPIPRHDGRDGVPSMTLSVRLRSQFMDSQGFTSDTG
WGCMIRSGQSLLANALSILVLGRDWRRGTRVDEEARILSLFAESPNAPFSIHRFVKHGAEYCDKYP

GQWFGPSAAARCIQALSAQCDDPKLRVYVTNDTSDVYEDRFMEVACDGSGRVQPILILLGIRLGID
HVTPVYWEGLKAALQYPQSVGIAGGRPSASHYFVGVQGSHYFYLDPHHTRPALPDHQIDRPYGRE
ELDTYHTRRLRRIHAKDMDPSMLIGFLIKDEHDWRDWKRRVASVPGKPIAYVHDRAEPEHGQGRS
EALDEVEALDDFEISG

>tr|A0A4P7NF89|A0A4P7NF89_PYROR Cysteine protease OS=Pyricularia oryzae
OX=318829 GN=PoMZ_07483 PE=3 SV=1

MDSAVAGAADIGRYGRRIVRMIWDPEPTNDPIANRPAWCLGYEYTLETNITSKTKGEDSKLSTATSS
DQQRPPAQANKVPQMPSAQLPTEAAATALYGNTTPPTPEAALEPTKITSQPAAIDTPPDSVDSSFDS
SMAYDDVPDDGGWPPAFLNDFESRIWMTYRSGFEPIPRSTDPTASSRMSFAMRLKTMADQQAGF
TTDSGWGCMIRTGQSLLANSLLTCRLGRSWRRGQAPDEERKLLSLFADDPRAPYSIHNFVAHGAA
KCGKYPGEWFGPSATARCIHALANATENSFRVYSTGDLPDVYEDSFMEVAKPDGKTFHPTLILISTRL
GIDKINQVYWESLTATLQLPQSVGIAGGRPSSSHYFVGAQRSDEDQGSYLFYLDPHHTRPALPFHE
DPQLYTPSDVDSCHTRRLRRLHIREMDPSMLIGFLILDEENWHAWKSSVKHVQGKSIITVSEHDPS
KGSASGRPSAIDEVETLSDDDGDTVLDG

>tr|A0A4Z1HR75|A0A4Z1HR75_9HELO Cysteine protease OS=Botryotinia convoluta
OX=54673 GN=BCON_0163g00060 PE=3 SV=1

MTAADIGRYKRFVQYFWDPEPTNDTASQSPIWCLGKEYPILEKSPTSTITDSPPQEVQYLPAQSLPTN
EVTTPPDSTVGSLDSSSGYQNCDTANADGGWPSAFLDDFEAKIWLTYRSNFPAIAKSQDPKAFSA
MSLSVRLRSQLVDQGGFTSDTGWGCMIRSGQSLLANALLTLRMGREWRRGISSHEERKILSLFAD
DPRAPYSIHKFVEHGASACGKHPGEWFGPSATARCIQALSNNQAKSELRVYITGDGSDVYEDTFM
SIAKPNHSDFTPTLILVGTRLGLDKITPVYWEALKYSLQMPQSVGIAGGRPSSSHYFIGVQESDFFYL
DPHQTRLALPYKDNMDDYTTEDVDSCHTRRLRRLHIKEMDPSMLIAFLIRDENDWNEWRRAVKEV
QGKGVIHVADTDPASYGLGGERDGAIDEVETFDDDDDDTILDA

>tr|A0A4Z1J4T6|A0A4Z1J4T6_9HELO Cysteine protease OS=Botryotinia narcissicola
OX=278944 GN=BOTNAR_0022g00470 PE=3 SV=1

MTAADIGLYKRFVQYFWDPEPTNDTSSQSPIWCLGKEYPILEKSPTSTITDSPPQEVQYLPAQSLPN
NEVTTPPDSTVGSLDSSSGYQTCDTANADGGWPPAFLDDFEAKIWLTYRSNFPAIAKSQDPKAFSS
MSLSVRLRSQLVDQGGFTSDTGWGCMIRSGQSLLANALLTLRMGREWRRGISSHEERKILSLFAD
DPRAPYSIHKFVEHGASACGKHPGEWFGPSATARCIQALSNNQAKSELRVYITGDGSDVYEDTFM
SIAKPNHSDFTPTLILVGTRLGLDKITPVYWEALKYSLQMPQSVGIAGGRPSSSHYFIGVQESDFFYL

DPHQTRPALPYKDNMDDYTTEDVDSCHSRRLRRLHIKEMDPSMLIAFLIRDENDWNEWRRAVKEV
QGKGVIHVADTDPASYGLGGERDGAIDEVETFDDDDDDTILDA

>tr|A0A505HW94|A0A505HW94_ASPNG Cysteine protease OS=Aspergillus niger OX=5061 GN=CAN33_0033180 PE=3 SV=1

MSLIPVPADKNNTRENVMGTTNYRKPSEHAWPESFLLDFESRIWMTYRSNFPPIPRVEGDDKSAS
MTLGVRLRSQLVDTQGFTSDTGWGCMIRSGQSLLANALSMLVLGRDWRRGARFEEESQLLSLFA
DTPTAPFSVHRFVKHGAESCGKYPGEWFGPSATAKCIEALSSQCGNPTLKVYVSNDTSEVYQDKF
MDIARNTSGAFQPTLILLGTRLGIDNITPVYWDGLKAALQFPQSVGIAGGRPSASHYFVGAQGSHLF
YLDPHYTRPALPDRQEGELYSKEEVDTYHTRRLRRIHVRDMDPSMLIGFLIRNQEDWADWLKRIEA
VKGRPIIHVLKQMNPDHDQEAGALDQVEALDDIE

>tr|A0A5D3B2L2|A0A5D3B2L2_9TREE Autophagy-related protein 4 OS=Cryptococcus floricola OX=2591691 GN=B9479_002531 PE=3 SV=1

MPSPSTHAPRASMSNNLPSPPPPDRVPPPKGRTHQFKLLRKGKDRERRHPTDLEDDWTIEGMSP
DAVEDKEFLEDPEPAPEPERIVPEMPVREEAKKPPSKGRQLVKKTSRLFSRDKDREGDSSLAPSSSS
SLVASRQTSASSADSQPTGSASRTLPSAFIQRHSSMQSKSRSPRTSFGQPTHSRRASQDSQSSWQ
APRSIRSFASNDTEPSAPRGLPIPQRQGASIPSLSRQSLPQPIHVNSRSPETIPGRMSSWFSHLLPTA
ENQPASLGSSPGAEATSPIRKQPSVAASLFNAARQKAVDGVRHLLDSEAQPDKCMDTIWMRGVG
HPGYRPKTPDTTSSPLPSVEGVQEEGRRSSLSMNRPSPTGLRPSSWRRNASISQGIPTQTNQSTPP
NKGFTNLFNSSSLSLAMPMGSSPSKEKEGPDSPNKKKPGKEVLQWPEQFYDDFRGTVWCTYRN
QYSPILALSPNLLIPSPEAYYASFSSPLDVASAPPAPPSPQQTPTQNSSFQAQQAPGGWWGKEERG
LTSDAGWGCMLRTGQSLLVNAIVHIHLGRDWRAIRPPTPPTSAAEAEALEQYAKYIQIISWFLDDPS
PLCPFSVHRMALIGKELGKEVGEWFGPSTAAGALKTLANSFAPCGVSVATATDSIIYKSDVYAASNLS
SDAWDQLIPPFSIGRPNQTDRGNKSGSGQKWGHKAVLVLVGVRLGLDGVNPIYHDSIKTLFTFPQ
SVGIAGGRPSSSYYFIGTQANSLFYLDPHLTRPAVPLQVPPLPTLPTEEEEVVVIPSPQQRPHTPSRS
RSRPAGSPTKASPLRNSLDINPEVAKYKLDVVNVDDVSESDSGGEGERVRSPKKEAIRRGVTSLTGT
PSKNGVKEMREDVGSPHKRAPSVSPTKGSINYSYPLSANSSQPASGSSTPAREVPEMTSRPKVDPH
AEWYTEAYPEHLMRTYQCEKVKKLPISGLDPSMLLGFVVRDEKDFEDFIDRVNKLPKKIFTIQNEQP
QWEEEDDVGLESVSEPELESEDDFDAPGTAKPRFEAGSPIDSDMMSGPSVLAASELDMDKIPDIPS
ATATPQVAREDYVGMSGSASASGSGTGPTASSSTSAPTTLDEDDGDGSLLDGSMETTVGAVDIAKH
LQRVDIANNDGEEEDGELVGGTPSSGGVMVEPPSVHSTPNRVRPVADRQDTARPVSSSPEEIPRQ
VRQGAESGQEEEEVDSKATSGTELPPMRNRMESWVEPIRGGQEAPNGDNLL

>tr|A0A5M9JAN0|A0A5M9JAN0_MONFR Cysteine protease OS=Monilinia fructicola OX=38448 GN=EYC84_008950 PE=3 SV=1

MLVLLDTYLGRQYKTRQDNTIQYNTIQYTSSPFPNFSTSQLLNNIPISTQLQSALTGVNGPYLIYLLHT
LQYTFISSVIGSIFLASLVLFRPPLFLTPTLVKSYVVTKRLVQYFWDPEPTNDTASRSPIWCLGEEYLVP
EKSSTSAPVIDSPPQEGGYLPAQSAQSPPPTTPSPRLHQIVKDGGWPTAFLDDFEAKIWLTYRSNFP
AIPKSQDPKAFSAMSLSVRLRSQLVDQGGFTSDTGWGCMIRSGQSLLANALLTLRMGREWRRGL
SSNEERKILTLFADDPRAPYSIHKFVEHGASACGKHPGEWFGPSATARCIQAAGVYITGDGSDVYED
TFMSIAKPNNTDFTPTLILVGTRLGLDKITPVYWEALKYSLQMPQSVGIAGGRPSSSHYFIGVQESDF
FYLDPHQTRPALPYKDNVEDYTVEDIDSCHTRRLRRLHIKEMDPSMLIAFLIRDENDWKEWRRAVK
EVQGKGVIHVADKDPALYGLGGERDGAIDEVETFDDDDDDTILDAPSIFVHSSLKRLILQGYKANTN
HTQCSK

>tr|A0A5M9MEY0|A0A5M9MEY0_9EURO Cysteine protease OS=Aspergillus tanneri OX=1220188 GN=ATG4 PE=3 SV=1

MNMNSVDIGRCGKRIVQYLWDPEPRNDEPDAALWCLGIRYPSHQQGVASQEIYNNDSFVKAKSP
PQETTVHGWPEEFLIDFESKIWMTYRSNLTPIPKLTSSDADLSMSLSVRLRSQLMDSQGFTSDTGW
GCMIRSGQSLLANALSILILGRDWRRGRNANEEAQLLYLFADHPDAPLSIHRFVKYGSESCGKYPG
EWFGPSATARCIEALLPQCEGPITKVYVTNENPDIHEESFLKVARDDSGVLRPTLILIGTRLGIDHITPV
YWGALKAALHFPQSVGIAGGRPAASHYFIGTQGSYFFYLDPHNTRPAIPFREPGVPYSEEEIDTYHT
RRLRRIHIKDMDPSMLIGFLIKDENDWLDWKARVAAVHGTPIIHVLTEGGTGYCQGRVEALDEVESL
DDGE

>tr|A0A5N5XBR8|A0A5N5XBR8_9EURO Cysteine protease OS=Aspergillus leporis OX=41062 GN=BDV29DRAFT_189199 PE=3 SV=1

MNSVDIGRCSKRIVQYLWDPEPRNDEEPDTPVWCLGIEYPPHQQKHTSPTTSNRDEPDTGTSTSD
DVTAHGWPEAFVSDIESKIWMTYRSNFTPIPTPDNDGTNHPMTLTVRLRTQLMDSQGFTSDTGWG
CMIRSGQSLLANAMFILQLGRDWRRGEKTEEEASMLSLFADHPDAPLSIHRFVKHGAESCGKYPG
EWFGPSATARCIEALSAQCGLPAPRVYVTNDTSDVYEDRFMRVSCSDSGSIQPTLILIGIRLGIDNVT
PVYWDGLKSMLQLPQSVGIAGGRPSASHYFIGTQGSFLFYLDPHNTRPAPPYNNAGKPISRDEINT
YHTRRLRRIHLQDMDPSMLMGFLIKNRDDWEGWKHRIASTPGKPIIHVFSRSDTAYCQGRKEALD
EVEALDDE

>tr|A0A5N6D5G9|A0A5N6D5G9_ASPPA Cysteine protease OS=Aspergillus parasiticus OX=5067 GN=BDV34DRAFT_207050 PE=3 SV=1

MNSVDIGRCRKRIVQYIWDPEPRNDEEPDASIWCLGVEYAPQLQKITANTTPDQDELEAGSSRIDD
VTAHGWPEAFVSDFESKIWMTYRSDFPPIPRPDNDEANHPMTLTVRIRTQLMDPQGFTSDTGWGC
MIRSGQSLLANAMLTLCLGRDWRRGDKAEEEARLLSLFADHPDAPLSIHRFVKYGAESCGKYPGE
WFGPSATARCIEALSAQCGNIAPRVYVTNDTSDVYEDSFLRVARSGSGSIQPTLILLGTRLGIDNVTP
VYWDGLKAVLQLPQSVGIAGGRPSASHYFIGTQGPHLFYLDPHTTRPALPYSIDGRLLSKTEISTYHT
RRLRRIHIQDMDPSMLIGFLVKDEDDWEDWKGRVGSVMGKQIIHVFKGAEATYNQGRRGALDEV
EALDDAE

>tr|A0A5N6EEE9|A0A5N6EEE9_9EURO Cysteine protease OS=Aspergillus novoparasiticus OX=986946 GN=BDV33DRAFT_181369 PE=3 SV=1

MNSVDIGRCRKRIVQYIWDPEPRNEEPDASIWCLGVEYAPQLQKITANTTPDQDELEAGPSRIDDV
TAHGWPEAFVSDFESKIWMTYRSDFPPIPRPDNDEANHPMTLTVRIRTQLMDPQGFTSDTGWGC
MIRSGQSLLANAMLTLCLGRDWRCGDKAEEEARLLSLFADHPDAPLSIHRFVKYGAESCGKYPGE
WFGPSATARCIEALSAQCGNIAPRVYVTNDTSDVYEDSFLRVARSGSGSIQPTLILLGTRLGIDNVTP
VYWDGLKAVLQLPQSVGIAGGRPSASHYFIGTQGPHLFYLDPHTTRPALPYSIDGRLLSKTEISTYHT
RRLRRIHIQDMDPSMLIGFLVKDEDDWEDWKGRVGSVTGKQIIHVFKGAEATYNQGRRGALDEVE
ALDDAE

>tr|A0A5N6GSA8|A0A5N6GSA8_ASPFL Cysteine protease OS=Aspergillus flavus OX=5059 GN=BDV35DRAFT_381631 PE=3 SV=1

MNSVDIGRCRKRIVQYIWDPEPRNDEEPDASIWCLGVEYAPQPQKITANTTPGKLGNYQDELEAGT
SKIDDVTAHGWPEAFVSDFESKIWMTYRSDFPPIPRLDNDEANHPMTLTVRIRTQLMDPQGFTSDT
GWGCMIRSGQSLLANAMLTLCLGRDWRRGDKAEEEARLLSLFADHPDAPLSIHRFVKYGAESCG
KHPGEWFGPSATARCIEALSAQCGNIAPRVYVTNDTSDVYEDSFLRVARSGSGSIQPTLILLGTRLGI
DNVTPVYWDGLKAVLQLPQSVGIAGGRPSASHYFIGTQGPYFFYLDPHTTRPAVPYSIDGRLLSKTEI
STYHTRRLRRIHIQDMDPSMLIGFLVRNEDDWEDWKGRVGSVVGKQIIHVFKGEEATYNQGRRGA
LDEVEALDDAE

>tr|A0A5N6HHI9|A0A5N6HHI9_9EURO Cysteine protease OS=Aspergillus pseudonomiae OX=1506151 GN=BDV37DRAFT_234878 PE=3 SV=1

MNSVDIGRCSKRIVQYLWDPEPRNDQKPDASIWCLGVEYAPRFKKYAANRTPDRDEPDAGTNTID
DVTEHGWPEAFVSDFESKIWMTYRSNFPPIPRPDNDEANHPMTLTVRLRTQLMDPQGFTSDTGW
GCMIRSGQSLLANAMLTLCLGRAWRRGDKSEEEAHLLSLFADHPDAPLSIHRFVKHGAESCGKHP
GEWFGPSATARCIEALSAQCGNIAPRVYVTNDTSDVYEDSFLRVARSGSGSIQPTLILLGTRLGIDNV

TPVYWEGLKAVLQLPQSVGIAGGRPSASHYFIGTQGSHLFYLDPHTTRPALPYSTGGRFPSKEEISTY
HTRRLRRIHIQDMDPSMLIGFLVRNEDDWDDWKGRIGSMMGKPIIHVLKGAETTYNQGRREALDE
VEALDDAE

>tr|A0A5N6ISN2|A0A5N6ISN2_9EURO Cysteine protease OS=Aspergillus minisclerotigenes OX=656917 GN=BDV30DRAFT_216564 PE=3 SV=1

MNSVDIGRCRKRIVQYIWDPEPRNDEEPDASIWCLGVEYAPQPQKITANTTPDQDELEAGTSRIDD
VTAHGWPEAFVSDFESKIWMTYRSDFPPIPRLDKDEANHPMTLTVRIRTQLMDPQGFTSDTGWGC
MIRSGQSLLANAMLTLCLGRDWRRGDKAEEEARLLSLFADHPDAPLSIHRFVKYGAESCGKHPGE
WFGPSATARCIEALSAQCGNIAPRVYVTNDTSDVYEDSFLRVARSGLGSIQPTLILLGTRLGIDNVTP
VYWDGLKAVLQLPQSVGIAGGRPSASHYFIGTQGPHFFYLDPHTTRPALPYSIDGRLLSKTEISTYHT
RRLRRIHIQDMDPSMLIGFLVRNEDDWEDWKGRVGSVVGKQIIHVFKGEEATYNQGRRGALDEVE
ALDDAE

>tr|A0A5N6SB16|A0A5N6SB16_ASPPS Cysteine protease OS=Aspergillus pseudotamarii OX=132259 GN=BDV38DRAFT_276211 PE=3 SV=1

MNSVDIGRCRKRIVQYLWDPEPKNDEEPDASIWCLGVEYASQLPKPTANKTSERDELDTGTSTIDD
VTAYGWPEAFVSDFESKIWMTYRSDFPPIPRPDNDEANHSMTLTVRLRTQLMDPQGFTSDTGWGC
MIRSGQSLLANAMLTLCLGRAWRRGDKAEEEAHLLSLFADHPDAPLSIHRFVKHGAESCGKFPGE
WFGPSATARCIEALSAQCGNIAPRVYVTNDTSDVYEDSFLRVARNGSGSIQPTLILLGTRLGIDNVTP
VYWDGLKAVLQLPQSIGIAGGRPSASHYFIGTQGSHLFYLDPHTTRPALPYSIDGRFLSKEETSTYHT
RRLRRIHIQDMDPSMLIGFLVKNEDDWEDWKGRIGSIAGKRIIHVFKGVETTYNQGRREALDEVEVL
DDAE

>tr|A0A5N6TRQ1|A0A5N6TRQ1_9EURO Cysteine protease OS=Aspergillus avenaceus OX=36643 GN=BDV25DRAFT_130905 PE=3 SV=1

MNSVDIGRCGKRIAQYLWDPEPRNDADPEASIWCLGVEYPPHKRKNALLTTSSQDTGADTNDVTA
HGWPEAFLLDFESKVWMTYRSNFPPIARPESRAPNYNMTLSVRLRSQLMEPEGFTSDTGWGCMI
RSGQSLLANAMFMLHLGRDWRCGEKAEEEAHILSQFADHPNAPLSIHRFVQHGAKSCGIYPGEW
FGPSATARCIETLSEECGNLSPKIYVTNDTSDVHEDTFMSVARNHRGDLQPTLILIATRLGIENVTPVY
WDGLKAALQLPQSVGIAGGRPSASHYFVGTQGSHLFYLDPHTTRTALPFSDVSEALSKDISTYHTRR
LRRIHVRDMDPSMLIGFLIKNEEDWEDWKRRIMATDGRPIIHIFSKRDIALRQSREEALDEVEALDD
E

>tr|A0A5N6V599|A0A5N6V599_ASPTM Cysteine protease OS=Aspergillus tamarii OX=41984 GN=BDV40DRAFT_285571 PE=3 SV=1

MNSVDIGRCRKRIVQYLWDPEPRNDEEPDASIWCLGVEYASQLPKPTANTTSSKPGNHRDELDAK
TSTIDDVTAHGWPEAFVSDFESKIWMTYRSDFPPIPRPDHDEANYSMTLTVRLRTQLMDPQGFTSD
TGWGCMIRSGQSLLANTMLTLCLGRAWRRGDKAEEEAHLLSLFADHPDAPLSIHRFVKHGAESC
GKYPGEWFGPSATARCIEALSTQCGNIAPRVYVSNDTSDVYEDSFLRVARNGSGSIQPTLILLGTRL
GIDNVTPVYWDGLKAVLQLPQSVGIAGGRPSASHYFIGTQGSHLFYLDPHTTRPALPYSIDGKFLSK
EEISTYHTRRLRRIHIQDMDPSMLIGFLVKNEDDWEDWKRRIGSIAGKRIIHVFKGVETTHNQGRRE
ALDEVEALDDAE

>tr|A0A5N6VKG2|A0A5N6VKG2_9EURO Cysteine protease OS=Aspergillus transmontanensis OX=1034304 GN=BDV41DRAFT_591789 PE=3 SV=1

MNSVDIGRCRKRIVQYIWDPEPRNDEEPDASIWCLGVEYAPQLQKIRANTTPGKLVNNQDELEAG
SSRIDDVTAHGWPEAFVSDFESKIWMTYRSDFPPIPRPDNDEANHPMTLTVRIRTQLMDPQGFTSD
TGWGCMIRSGQSLLANAMLTLCLGRDWRRGDKAEEEARLLSLFADHPDAPLSIHRFVKYGAESCG
KYPGEWFGPSATARCIEALSAQCGNIAPRVYVTNDTSDVYEDSFLRVARSGSGSIQPTLILLGTRLGI
DNVTPVYWDGLKAVLQLPQSVGIAGGRPSASHYFIGTQGPHLFYLDPHTTRPALPYSIDGRLLSKTE
ISTYHTRRLRRIHIQDMDPSMLIGFLVKDEDDWEDWKGRVGSVMGKQIIHVFKGAEATYNQGRRG
ALDEVEALDDAE

>tr|A0A5N6WMJ0|A0A5N6WMJ0_9EURO Cysteine protease OS=Aspergillus sergii OX=1034303 GN=BDV39DRAFT_12602 PE=3 SV=1

MNSVDIGRCRKRIVQYIWDPEPRNDEEPDASIWCLGVEYAPRLQKITANTTPDQDGLEAGPSRIDD
VTAHGWPEAFVSDFESKIWMTYRSDFPPIPRPDNDEANHPMTLTVRIRTQLMDPQGFTSDTGWGC
MIRSGQSLLANAMLTLCLGRDWRRGDKAEEEARILSLFADHPDAPLSIHQFVKYGAESCGKYPGE
WFGPSATARCIEALSAQCGNIAPRVYVTNDTSDVYEDSFLRVARSGTGRIQPTLILLGTRLGIDNVTP
VYWDGLKAVLQLPQSVGIAGGRPSASHYFIGTQGPHLFYLDPHTTRPALPYSIDGRLLSKTEISTYHT
RRLRRIHIQDMDPSMLIGFLVKDEDDWEDWKGRVGSVMGKPIIHVFKGAEATYNQGRRAALDEVE
ALDDAE

>tr|A0A5N6XTZ5|A0A5N6XTZ5_9EURO Cysteine protease (Fragment) OS=Aspergillus arachidicola OX=656916 GN=BDV24DRAFT_141680 PE=3 SV=1

HQKITANTTPDRDELEAGPSRIDDVTAHGWPEAFVSDFESKIWMTYRSDFPPIPRLDNDEANHPMT
LTVRIRTQLMDSQGFTSDTGWGCMIRSGQSLLANAMLTLCLGRDWRRGDKAEEEARLLSLFADHP
DAPLSIHRFVKYGAESCGKYPGEWFGPSATARCIEALSAQCGNIAPRVYVTNDTSDVYEDSFLRVAR
RGSGSIQPTLILLGTRLGIDNVTPVYWDGLKAVLQLPQSVGIAGGRPSASHYFIGTQGPHLFYLDPH
TTRPALPYSIDGRLLSKTDISTYHTRRLRRIHIQDMDPSMLIGFLVKDEDDWEDWKGRVGSVMGKQI
IHVFKGAEATYNQGRRGALDEVEALDDAE

>tr|A0A5N6YU93|A0A5N6YU93_9EURO Cysteine protease OS=Aspergillus coremiiformis OX=138285 GN=BDV28DRAFT_69539 PE=3 SV=1

MNSVDIGRCSKRIVQYLWDPEPRNNEEPNASIWCLGIEYTTHKASLQTTNNQNEPDTGALTLDDVT
AHGWPEAFVSDFESKIWMTYRSSFPTIPKLDNDEANHPMTLTVRLRSQLMEPQGFTSDTGWGCMI
RSGQSLLANAMSTLHLGRAWRRGEKSQEEARLLSLFADHPDAPLSIHQFVKHGAEACGKYPGEW
FGPSATARCIEALSVQCGNLAPKVYITNDTTDIYEDSFRRVACSDSGSIQPTLILLGTRLGIENVTPVY
WTGLKAVFQFPQSVGVAGGRPSASYYFIGTQGSYLFYLDPHSTRPASPYNVAGESLSREEINTYHTR
RLRRIHMQDMDPSMLIGFLVRNEDDWEDWKRRIALTAGKPIIHVFSGVDTPHFPGRKDAVDEVEAL
DDLE

>tr|A0A5N6ZZC9|A0A5N6ZZC9_9EURO Cysteine protease OS=Aspergillus caelatus OX=61420 GN=BDV27DRAFT_130633 PE=3 SV=1

MNSVDIGRCRKRIVQYLWDPEPRNDEEPDASIWCLGVEYASQLPKPTANTTSERDELDAGTSTIDD
VTAYGWPAAFISDFESKIWMTYRSDFPPIPRPDNDEANHSMTLTVRLRTQLMDPQGFTSDTGWGC
MIRSGQSLLANAMLTLCLGRAWRRGDKAEEEAHLLSLFADHPDAPLSIHRFVKHGAESCGKYPGE
WFGPSATARCIEALSAQCGNMAPRVYVTNDTSDVYEDSFLRVARNGSGSIQPTLILLGTRLGIDNVT
PVYWDGLKAVLQLPQSVGIAGGRPSASHYFIGTQGSHLFYLDPHTTRPALPYSIDGRFLSNEEISTY
HTRRLRRIHIQDMDPSMLIGFLVKNEDDWEDWKGRIGSIAGKRIIHVFNGAETTYNQGRREALDEV
EVLDDAE

>tr|A0A5N7AZW5|A0A5N7AZW5_9EURO Cysteine protease OS=Aspergillus bertholletiae OX=1226010 GN=BDV26DRAFT_283577 PE=3 SV=1

MNSVDIGRCSKRIVQYLWDPEPRNDEEPNASIWCLGVEYAPHTQKPTGNTTSGKPKNYKDELDTG
ANVTAYGWPEAFVSDFESKIWMTYRSNFPPILRSDNDEANHPMTLTVRLRNQLMDPQGFTSDTG
WGCMIRSGQSLLANAMLTLCLGRAWRYGDKAEEEAHLLSLFADHPDAPLSIHRFVKHGAESCGK
HPGEWFGPSATARCIEALSAKCGNIAPKVYVTNDTSDVYEDSFLRVARSGSDSLQPTLILVGTRLGID
NVTPVYWDGLKAILQLPQSVGIAGGRPSASHYFIGTQGSHLFYLDPHTTRPALSYYIDGKLSKEETST

YHTRRLRRIHIRDMDPSMLIGFLVRNESDWEDWKRRIGSILGKPIIHVFEGAETTYNQGRAEALDEV
EALEDAE

>tr|A0A7R7X8L2|A0A7R7X8L2_ASPKA Cysteine protease OS=Aspergillus kawachii OX=1069201 GN=ATG4 PE=3 SV=1

MNTVDIGRCSKRIVQYLWDPEPRNDEDPTSSIWCLGIEYHPEKDVSPRGETPDKNSARDNTTGTTN
YRKPSEHAWPESFLLDFESRIWMTYRSNFPPIPRVEGDDKSASMTLGVRLRSQLVDTQGFTSDTG
WGCMIRSGQSLLANALSTLVLGRDWRRGARFEEESQLLSLFADTPTAPFSVHRFVKHGAESCGKF
PGEWFGPSATAKCIEALSSQCGSPTLKVYVSNDTSEVYQDRFMNVARNSSGVFQPTLILLGTRLGID
HITPVYWDGLKATLQLPQSVGIAGGRPSASHYFVGAQGSHLFYLDPHYTRPALPDRQGGELYSKEE
VDTYHTRRLRRIHVRDMDPSMLIGFLIRDQEDWDDWLNRIQAVKGRPIIHVLKQMNPDHDQEAEA
LDQVEALDDIE

>tr|A0A7R7ZSE0|A0A7R7ZSE0_ASPCH Cysteine protease OS=Aspergillus chevalieri OX=182096 GN=ATG4 PE=3 SV=1

MNSVDLGRCKRIVQYLWDPEPKNDEEPSSPIWCLGKEYNRHVPVTTSKGDTRPQMVAFEEDGHD
PEDLAQDATTQTHETALGWPKAFVRDFESRIWITYRSNFVPIPRPQDHDANPNMTLSVRLRSQLMD
SQGFTSDTGWGCMIRSGQSLLANGLSSLLLGRDWQRGSKADEEIRLLSLFADFPDAPFSIHRFVEL
GAERCGKYPGEWFGPSATALCIQALCEQCQEPKLRVYVSNENANVYQHKFMEIARDDAGYIRPTLI
LLGTRLGIDHITPVYWDALKAALQYPQSVGIAGGRPSASHYFVGVQGSHLFYLDPHHTRPALPYRPA
HELYSEEERDTYHTRRLRRIHIKDMDPSMLLGFLIKNEEDWEDWKKRVEAGRGKPIIHILGDMQPD
YGLGREEALDEVEALDDIDGMVS

>tr|A0A7R8AN13|A0A7R8AN13_9EURO Cysteine protease OS=Aspergillus puulaauensis OX=1220207 GN=ATG4 PE=3 SV=1

MNPTDIERYKKRIVQYIWDPEPKNDEEPESPIWCLGTGYPPSEQLITPREELSKTQEALPSDTEKDCA
QNRTLTLHVHHNGRQTNTTSISTPDWPEPFLLDFESKIWMTYRSNFTPIARDTSSEGNQSLTLGVRL
RSQFIEPQGFTTDTGWGCMIRSGQSLLVNAMAIVTLGRGWRRRDKIEEEAQMLSLFADSPDAPFSI
HNFVKYGAEFCGKQPGEWFGPTATARCIQGLSANCSQATLKVYIADDNSDVYQDRFMSASRNEQ
GIVSPTLILLALRLGIDRVTTVYWEGLKAVLQFPQSVGIAGGRPSASHYFIAVQGSHFFYLDPHNTRP
APNYTKLGTYTKDEVDTYHTRRLRRLHIRDMDPSMLIGFLIKDEEDWEDWKTRVASTQGKPIINILSA
GDTTPWQGRREALDEVEAFDDE

>tr|A0A7U2R4G0|A0A7U2R4G0_ASPFN Cysteine protease OS=Aspergillus flavus (strain ATCC 200026 / FGSC A1120 / IAM 13836 / NRRL 3357 / JCM 12722 / SRRC 167) OX=332952 GN=F9C07_12140 PE=3 SV=1

MYPYIYIERHHNNHNAQKQKISSTALSLQTHRLHPKMNSVDIGRCRKRIVQYIWDPEPRNDEEPDA
SIWCLGVEYAPQPQKITANTTPDQDELEAGTSKIDDVTAHGWPEAFVSDFESKIWMTYRSDFPPIPR
LDNDEANHPMTLTVRIRTQLMDPQGFTSDTGWGCMIRSGQSLLANAMLTLCLGRDWRRGDKAEE
EARLLSLFADHPDAPLSIHRFVKYGAESCGKHPGEWFGPSATARCIEALSAQCGNIAPRVYVTNDT
SDVYEDSFLRVARSGSGSIQPTLILLGTRLGIDNVTPVYWDGLKAVLQLPQSVGIAGGRPSASHYFIG
TQGPHFFYLDPHTTRPAVPYSIDGRLLSKTEISTYHTRRLRRIHIQDMDPSMLIGFLVRNEDDWEDW
KGRVGSVVGKQIIHVFKGEEATYNQGRRGALDEVEALDDAE

>tr|A0A854QH91|A0A854QH91_CRYNV Autophagy-related protein 4 OS=Cryptococcus neoformans var. grubii Tu259-1 OX=1230072 GN=C361_02147 PE=3 SV=1

MSSPASTSPKSSFTFSPTSFPHASIASTNVRPRTNLPPPPPPDRIPPPKGRSHQQKFKILRKGKDKDK
RQPTDLEDDWTIEDVTVNNGNVEQEPQYLDDVQPEENELKPEPRLVEMANREEKKEKTFKSRELV
KKTSRLFGRDKDKDRDKSEEPAGSSSSTLAAMRQSSSTSTDSTTSRSITSAFTRQNSIQSRRSPRTSF
GQAHSRRTSQDSQMSWPAPRSIRSSITPHDPPNDPQNSANNTGVPIPQRQGASMSSLSRYSLPQ
PNGNASRSPDTFPNKMSTWFSHLLPVSSESPPSSNYETSPSVRKQPSVAASLFNAARQKAVDGVR
HLLDSEAQPDKCMDTIWVRGVAHPGWRPITPENGTSNLPTIEPGGGGGGVEDRRASLSMNRPSP
TSLRPSSWKRNTALPPTAQPQPSTQQHTQASNQTTSPSKGFTGIWNSSTLSLGMPIGGSPNKERES
GSGAESPSKKKNKEIVKWPEQFYDDFRSTVWCTYRSQYAPISSLSPNLLIPSPEAYYASFGPPLDATS
PSSPRVTIPTTAAQQTASGSGGWGWSKEERGLTSDAGWGCMLRTGQSLLVNALIHVHLGRDWRV
PSTPASFSEATTNQETAALKDYAKYAQMLSWFLDDPSPLCPFSVHRMALIGKELGKEVGEWFGPST
AAGALKTLANSFAPCGVAVATATDSIIYKSDVHTASNLPSDDWNSISPTFNSSKKKRGGDNKAKEGK
WGKRAVLILVGIRLGLDGVNPIYYDSIKALFTFPQSVGIAGGRPSSSYYFIGSQANHLFYLDPHLTRPA
IPLQVPPLPVHSAKEEGSMENSSILSTTEEESEEGVMIRTPETPRSTTPSTFSAPEQIEDEGQEEWEQ
GSKYKLDVVDADGAKVEEIDDDKGWNEGETIKEEIPKNAFESKGVVQDHSEKQRGSASTASVDSQ
VDPQMLWYTTAYPDSLLRTYHCEKIKKMPLSGLDPSMLLGFVCKDEDDFEDFVERVAQLPKKIFTV
QDEMPSWEEDDDAGLESVSEPDFEGDELEEPGTAKPRFDSSSPVNEDSLKGPRVVSASTTATPLA
AKEGHLDVEETNSTDDDDESIGTTTAVRPVDIARYLHRVDLSSKREQEDDDDGEWVGGTPSSQGV
LVEPPSLKGTPSKSRSSALEPRHEQNGEKEQERHVFPARNRMESWVEPVCEGKEAPNGDNLL

>tr|A0A8H3N823|A0A8H3N823_9EURO Cysteine protease OS=Aspergillus udagawae OX=91492 GN=Aud_002850 PE=3 SV=1

MNGVDMERCKRIIQYIWDPAPRNDQPEDPIWCLGTKYTTDGTERGNRRTASPKPMRLANEEGPS
QLDPKATMPGLLDEQGWPESFLLDFESRIWITYRSNFPPIPKPIKQDAYSAMTLSVRLRSQLVDQH
GFTSDTGWGCMIRSGQSLLANAMSILLFGRGWRRGINTDQEAQLLSQFADHPDAPFSIHRFVQH
GAESCNKHPGEWFGPSATARCIQALVSQHGSLNLGVYMTDDTADVHEDKFLDAAHDERGSFRPT
LILIGTRLGIDRITPVYWDAVKTTLQLPQSVGIAGGRPSASHYFVGVQGSHLFYLDPHQTRPALPQRS
IDEQYTNEEIETYHTRRLRRIHVRDMDPSMLIGFIIKDKEDWTHWKSGVSVQGKAIVHVLSESDTAVF
HGREGAIDEVEVLDDD

>tr|A0A8H4D859|A0A8H4D859_9ASCO Cysteine protease OS=[Candida] haemuli var. vulneris OX=1231523 GN=FT663_00277 PE=3 SV=1

MSSEPSKEEHKASLAEPVPSAASDSKVPLETSPPHSNLQGPQEIVDSGTKSAAEQPNSFQNALAQF
WQRLRSPDAPETDPESLQPVVIMGTSYKGTLTSAGIEEAIRRRLWFTYRTGFEPIPRALDGPSPLSFIG
SMIVHGVNPHGALAGFLDNTSFTSDVGWGCMIRTAQCLLANTLQTVIARKAIQDGNTPDWSSINT
QVVSLFRDNYNSPFSLHNFVGVASHSPLKVKPGQWFGPSAASLSIKRLCSKANEDADFPKLNVMI
CESCDLHGDEVKSLLEKTSRPLLLLFPIRLGIETINAIYYPSLFQLLSLPHSMGISGGKPSSSYYFFGH
QGDSLLYLDPHTSQQIDETNGSYRPLSCNTVPISGLDPSMVVGLLVESYADYEALKSELKGKNKIVH
FHDRAPTRRSVSVSNQDKNAKSAEPDGNGASDIGDFVDIGDDFAGSEVEEEIQEDPNSSVTAKYDI
VEGPAPVTVLEDEK

>tr|A0A8H4F3B9|A0A8H4F3B9_MUCCL Cysteine protease OS=Mucor circinelloides f. lusitanicus OX=29924 GN=FB192DRAFT_1048174 PE=3 SV=1

MTHHSNEQKHVDNELPQPHNSNHISTAPLKDKLLTNSSYLAAEIPLKLGHFMSNLWISGSELSSSF
FLQNGDCNIDSENAQIIWLLGRPYQTKPLDQMQQAILDAQKDEMFRPGGTAADRDDEEVHKDNV
SMVWPPDFYDDFTSRLWMTYRHNYPPIRPSNHKTDIGWGCMLRSGQSLLANTLLIHFLSRDWRR
QKQTAAARLQYAKVMLESGFAQNELSILFVRLFIGSWMSCRREHPFRSIE

>tr|A0A8H4M8S1|A0A8H4M8S1_9EURO Cysteine protease OS=Aspergillus fumigatiaffinis OX=340414 GN=CNMCM6457_010064 PE=3 SV=1

MNGIDMERCKRIIQYIWDPAPRNDQPGDPIWCLGTKYTTDGMERDNRRTATPKLMGMANEEGPN
QLVPKATTPGQLDEQGWPESFLHDFESRIWITYRSNFPPIPKPINQDAFSAMTLSVRLRSQLVDQH
GFTSDTGWGCMIRSGQSLLANAMSILLFGRGWRRGIDTDREAQLLLQFADHPDAPFSIHRFVQHG
AESCNKHPGEWFGPSATARCIQALVSQHGSLNLGVYMTDDTADVQEDKFLDAAHDERGSFRPTLI
LIGTRLGIDRITPVYWDAVKATLQLPQSVGIAGGRPSASHYFVGVQGSHLFYLDPHQTRPALPRRNI

DEPYTDEEIETYHTRHLRRIHIRDMDPSMLIGFIIKDREDWAHWKSRVSVQGKPIVHVLSESDTAVF
QGREGAIDEVEVLDDD

>tr|A0A8H6BX97|A0A8H6BX97_CANAX Cysteine protease OS=Candida albicans OX=5476 GN=FOB64_004501 PE=3 SV=1

MNQPNPNKQPIVQSTLEQTSNEEVDTVLGRFTLFVKDLSNGLNGSQEIPPSQESVSEEAEVISRKIIV
LGQTFDNFDNANDYIESKLWLSYRCGFEPIPKSIDGPQPIQFFPSIIFNRKNFTSDAGWGCMIRTSQ
NLLANTLLKLYPKNEPEIVKLFQDGTSSPFSIHNFIRVASLSPLHVKPGEWFGPNAASLSIKRLTNELL
QDQELDGIKSPECLLAKF

>tr|A0A8H6UWN2|A0A8H6UWN2_9EURO Cysteine protease OS=Aspergillus hiratsukae OX=1194566 GN=CNMCM5793_007818 PE=3 SV=1

MNVVDMERCKRIIQYIWDPAPKNDQPGDPIWCLGTKYSVDDTERDNKRTASPKSMQPANEEGPN
QLVPKGTMPGLLDEQGWPESFLLDFESRIWITYRSNFPPIPKPIKQDAYSAMTLSVRLRSQLMEQH
GFTSDTGWGCMIRSGQSLLANAMSILLLGRGWRRGIDTEQEAQLLSQFADHPDAPFSIHRFVQH
GAEFCNKHPGEWFGPSATARCIQALVSQYGSLNLGVYMTDDTADVHEDKFLDAAHDERGSFRPTL
ILIGTRLGIDRITPVYWDALKTTLQLPQSVGIAGGRPSASHYFVGVQGSHLFYLDPHQTRPALPRHNI
DEPYTDEEIETYHTRHLRRIHIRDMDPSMLIGFIIKDEEDWAHWKSGVSAQGKPIVHVLSGSDTDVF
QGRDGAIDEVEVLDDD

>tr|A0A8H7R865|A0A8H7R865_9FUNG Cysteine protease OS=Mucor saturninus OX=64648 GN=INT47_008047 PE=3 SV=1

MSFEPNKTACASTPDQEYLPQPRNSNHISKAPLKDKLLTNSSYLAAEIPLKLGHFMSNLWTSGSELS
SSFFLHNHETETQQHIIWLLGNAYYNKPLDGIQQAILEAQKDEMFRPVQQENQVHTDTTSMSMLW
PSDFYDDFVSRLWMTYRHNYPPIRPSNHKTDIGWGCMLRSGQSLLANTLLIHFLSRDWRRQKQE
QTVYKQYSKILHWFLDELSPRAPFSIHRIALLGKQLGKNIGEWFGPSTISQVIQALVSDFAPANLSVYI
ATDGVIYRDDVQDVATGKKPRGDFSHLITRVSSDEGGRQEAKNLYEASNNDDAATPDRRDSEFKPV
LILVALRLGIDSLHPTYYPALKACFEIPSFVGIAGGRPNSSLYFIGLQGDDLIYLDPHFSRPALETKGLS
EYTKEDFGTYHCTLPRKINIANLDPSMLLGFYCRTAQDFDLLCDQLDLISQKHSAIVSVQQSAPEYD
EDVRSENDFGVLSDEDEELSVDDDDHSIF

>tr|A0A8H7V1D5|A0A8H7V1D5_9FUNG Cysteine protease OS=Mucor plumbeus OX=97098 GN=INT46_007647 PE=3 SV=1

MTQHFNEQKNTDKELPQPHNSNHISTAPLKDKLLTNSSYLAAEIPLKLGHFMSNLWISGSELSSSFF
LQNGECNPGSENTQIIWLLGCPYQSKPLDQMQQAILDAQKDEMFRPGADREDEEIHKDNVSLVW
PPDFYDDFTSRLWMTYRHNYPPIRPSNHKTDIGWGCMLRSGQSLLANTMLIHFLSRDWRRQKQT
TAAKKQYGKIIHWFLDELSPRAPFSIHRIALLGKQLGKNIGEWFGPSTISQVIQALVSDFQPADLSVYI
ATDGTIYLDGVQDVTTGKKPRGDFSYFSNKLSSSSSDEGRKEAKNLYDASSTPQEETTFPNDMLPD
DKDTVFKSVLILVPLRLGIDSLHPTYYSALKACFELPSFVGIAGGRPNSSLYFIGLQGDDLIYLDPHFS
RPALETKSLSEYTREDFSTYHCTIPRRIPISNLDPSMMLGFYCRTRKELDLLCDQIKTISQKYSAIFSIQ
QSAPEYDEDVRSENDFGVMSDEDDQLEEEYKNDGSDSDCNSIF

>tr|A0A8H8DEA9|A0A8H8DEA9_9ASCO Cysteine protease OS=Candida metapsilosis
OX=273372 GN=I9W82_000101 PE=3 SV=1

MIDPHSASEPREEGASTAVQSQSTDSTSGTGFDRFTSFFKGISGINIGFQEANENTEATEDTTTDSNE
LVILGNHYINQQEANSYIHSLLWLSYRCGFTPIPKAVDGPQPVTFFPSLLFSKSTLTNVGNLRSLFDN
DNFTSDAGWGCMIRTSQNLLANAILKLSSESNEATQLEILKLFQDDSEAVFSLHNFIRVASASPLLIK
PGQWFGPNAASLSIKKLVTEIKEQDLSVEIPCVYVSENADLYDDEIEELFVSEQKSLLLLFPVRLGIDQ
VNKYYYKSIFQLLGSRFSVGIAGGKPSSSFYFVGYQNDEELIYFDPHLPQIVETPINLASYHTTNCNK
LNIESLDPSMMVGVLLNSMADYKEFKQECIENKIIHFHPLAITSQSDSTMNQSWEEVQEDDDFVNL
TIPRSEEEYVVLDE

>tr|A0A8J5QN75|A0A8J5QN75_9ASCO Cysteine protease OS=[Candida] subhashii
OX=561895 GN=J8A68_003012 PE=3 SV=1

MDTNTNDPEPQTTDQNQNGIDTIIGRNLERFSAFFREFSTGTTTTGEGIIIDTKAQQQPEEDKRIFILG
QEFQNAQDADTFISSQVWLSYRCGFDPIVKAEDGPSPISFFPSIVFNKGIFANFANLRSLLDKENFN
SDAGWGCMIRTSQNLLATALIRLATTTADIDKDVIGLFQDKKDAPFSLHNFIRVAGESPLQIKPGQW
FGPNAASLSIKKLIDEIKDNQNGNIKYPNVFISENSDLYDDELKQLFKNGTDNSSVLVLLPMRLGIEQ
VNEYYYESILQLLRCKYSVGISGGKPSSSFYFLGYQNQSQLIYFDPHVSQLFEDPINYQSYHTKNHQ
YLDINALDPSMMIGILLKDEQEYRDFKIYCRENGNKIVYFHPQMTPIQDGTVGQSWEVVDQPDDD
FVNLNLMKSNQDEDEEEDEFVDLG

>tr|A0A8X7NLG1|A0A8X7NLG1_CANPA Cysteine protease OS=Candida parapsilosis
OX=5480 GN=FOB60_003180 PE=3 SV=1

MTEPQPASESQQDIEENATNQPRSYDVAPGSGFERLTSFFKGVSGINMGSQEAKDDTHSTTYNMN
TEQKSISILGNHFKTETEAKEYIQSLLWLSYRCGFTPIPKSADGPQPVSFLPSVLFSKSTLTNMSNLRG
LFDNDNFTSDAGWGCMIRTSQNLLAIALLKLSEEHNESAQLDILKLFQDDPTSPFSLHNFIRVASSS

PLLVKPGQWFGPNAASLSIKKLTIEAKKLETPGEIPYVYISENADLFDDEIEDLFNEEQKPLLLLFPVRL
GIDQVNKYYYKSILQLLSLPYSVGIAGGKPSSSFYFIGYENENHLLYFDPHLPQVVEAPINITTYHTAN
YNKLDIEMVDPSMMIGVLLKSMDEYKEFKQDCSENKIIHFHPLVITSQQDSALNQSWEEVQEDDD
FVNLTVPKSEEEFVVLDK

>tr|A0A9P0QTW5|A0A9P0QTW5_9ASCO Cysteine protease OS=[Candida] railenensis OX=45579 GN=CLIB1423_19S02454 PE=3 SV=1

MTSSDNIGRDGSGAPGAGANESIQADSSPVSRVLESNSESSSSSTPSITPMISQLWSNLAERASSS
LLSSSVPVSAIHVLGKSYQPEDTVPLNADILSKIWLTYRAGFEPISKAEGGPAPLSFINSMLFNRNISS
TISNLHSLTDNDYFTTDVGWGCMIRTSQSLLANALLKAGYGTSSSSSSSSSGIIDMFLDRSTAPFSL
HNFIKVAGESPLQVKPGEWFGPSAASLSIKRLCDSVSDSKVPQVLISESSDLYNDQIQNLLAAGRTV
LVLLPIRLGIDNISPYYYSSLFDLLTLPQSVGIAGGKPSSSYYFFGFQDSKLFYLDPHYPQQSNPNSD
TESVNYSTYHTTKYSTLDIGGMDPSMMAGFLIKDSEDYQNFTKSLLESKNKIIHFHEQREKERRSSTS
QQDISSDDFSLSQVEMAEEDFVQVGEETENGFVDLGDEVTVKEESVHISREEGVDKIDEVVSIARE
EAKDGGEGAGEASDLEGDGIKV

>tr|A0A9P3BDJ2|A0A9P3BDJ2_9EURO Cysteine protease OS=Aspergillus pseudoviridinutans OX=1517512 GN=Asppvi_006084 PE=3 SV=1

MNGVDMERCKRIIQYIWDPAPRNDQPELPIWCMGTKYTTDGMDRDNRHTASHKSMRLANEEGP
SQVVPKATTTSQLDEHGWPEPFLLDFESRIWITYRSNFPPIPKPIQQDAYSAMTLSVRLRSQLVDQH
GFTSDTGWGCMIRSGQSLLANAMSILLFGRGWRRGINTDQEAQLLSQFADHPDAPFSIHRFVQH
GAESCNKHPGEWFGPSATARCIQALVSQHRSLNIGVYMTDDTADVHEDKFLDAAHDERGSFRPTL
ILIGTRLGIDRITPVYWDAVKTTLQLPQSVGIAGGRPSASHYFVGVQGSHLFYLDPHQTRPALPQRNI
DEPYTDEEIETYHTRRLRRIHIRDMDPSMLIGFIIKDKEDWTHWKSGVSVQGKPIVHVLSESDTAVFQ
GREGAIDEVEALDD

>tr|A0A9P3BUR4|A0A9P3BUR4_ASPVI Cysteine protease OS=Aspergillus viridinutans OX=75553 GN=Aspvir_005025 PE=3 SV=1

MNGVDMERCKRIIQYIWDPAPRNDQPEEPIWCLGTKYTTDGMERDNRRNASPKSMRLVNEEGPS
QLVPKATMPGQLDEQGWPESFLLDFESRIWITYRSNFPPIPKPIKQDAYSAMTLSVRLRSQLVDQH
GFTSDTGWGCMIRSGQSLLANAMSILLFGRDWRRGINTDQEAQLLSQFADHPDAPFSIHRFVQH
GAESCNKHPGEWFGPSATARCIQALVSQHGSLNIGVYMTDDTADVHEDKFLDAAHDERGSFRPTL
ILIGTRLGIDRITPVYWDAVKTTLQLPQSVGIAGGRPSASHYFVGVQGSHLFYLDPHQTRPALPQRNI

DEPYTDEEIETYHTRRLRRIHLRDMDPSMLIGFIIKDKEDWAHWKSEVSVQGKPIVHVLSESDTAVF
QGREGAIDEVEVLDDE

>tr|A0A9P6XXA5|A0A9P6XXA5_RHIOR Cysteine protease OS=Rhizopus oryzae OX=64495 GN=G6F51_012029 PE=3 SV=1

MTSSEKKKEEMHINRNVGSTELPQPQNSNHLSTAPLKDKLITNSSYLAAEIPLKLGHFVSNLWTSGS
DFSNSFLHQQQQNDMNHNTIWILGRSYIINPTDHVQQALIEAQRDIMFKSTGEEISEEEDNNNMY
LRWPLDFYDDFTSRLWMTYRHNYPPIRPSNHKTDIGWGCMLRSGQSLLANTLIIHFLGRDWRRQT
QNQTTRKELCIGFLMSYHQEHPFQYTE

>tr|A0A9P6Y405|A0A9P6Y405_RHIOR Cysteine protease OS=Rhizopus oryzae OX=64495 GN=G6F51_009671 PE=3 SV=1

MTSSDQKMSESCTDLTDLEVNDKELPQPQNSNHLSTAPLKDKLLTNSSYLAAEIPLKFGHFMSNLW
TSGSELSSSLFLQQQQQQDNCIIWLLGCSYIIKPTDHIQQALLEAQRDLMFNKGSSENEEENNQ
NMHMLWPPDFYDDFTSRLWMTYRHNYPPIRPSSHKTDIGWGCTLRSGQSLLANTLIIHFLGRDW
RRQTQNQAAWKQYSRIVHWFLDELSPRAPFSIHRIALLGKQLGKNIGEWFGPSTISQVIQALVSDFA
PANLSVYVAADGVIYRDDVNDVATGKKPRGDFSYLSSLADSKGREEAKHLYDASTPQSSTVKTDTFK
PVLILVALRLGIDSLHPTYYGGLKACFELPSFVGIVGGRPNSSLYFIGLKGDELIYLDPHYSRPALETKS
LAQYTRKDFNTYHCTIPRKVHISNIDPSMLVGFYCQNISDFESLCQQITKTSKNHSAIISVEQSAPVYE
EDVRSENDFGIVSDDDDSDVDDIADK

>tr|A0A9P7BMY4|A0A9P7BMY4_RHIOR Cysteine protease OS=Rhizopus oryzae OX=64495 GN=G6F64_010624 PE=3 SV=1

MTSSEKKKKEMHINRNVGSTELPQPQNSNHLSTAPFKDKLITNSSYLAAEIPLKLGHFVSNLWTSGS
EFSYSFLQQQQNDMNHNTIWILGRSYIVNPTDHVQQALIEAQRDIMFKSPGEEISEEEDNNNMH
LRWPPDFYDDFTSRLWMTYRHNYPPIRPSNHKTDIGWGCMLRSGQSLLANTLIIHFLGRDWRRQT
QNQTTRKEYNKATKKCLKE

>tr|A0A9P8N7X1|A0A9P8N7X1_ASPFM Cysteine protease OS=Aspergillus fumigatus OX=746128 GN=ATG4 PE=3 SV=1

MNAVDMERCKRIVQYIWDPAPRNDQPGAPIWCLGTKYTTDGMERDNRHTATPESMRLANEEGPN
QLDPKAITPGHLDGQGWPESFLDDFESRIWITYRSNFPPIPKPIDQDAFSTMTLSVRLRSQLVDQH

GFTSDTGWGCMIRSGQSLLANAMSILLFGRGWRRGIDNDREAQLLSQFADHPDAPFSIHRFVQH
GAEFCNKHPGEWFGPSATARCIQALVSQHGNLNLGVYMTDDTADVYEDKFLDAANDGRGSFRPT
LILIGTRLGIDRITPVYWDAVKTTLQLPQSVGIAGGRPSASHYFVGVQGSHLFYLDPHQTRPALPQR
NIDDPYTDEEIETYHTRRLRRIHIRDMDPSMLIGFIIKDREDWAHWKSGVSAQEKPIVHVLSESNTAV
FKGREGAIDEVEVLDDD

>tr|A0A9R1CPD5|A0A9R1CPD5_9TREE Autophagy-related protein 4 OS=Cryptococcus cf. gattii OX=2011032 GN=D1P53_000637 PE=3 SV=1

MSSPTPASPTSSLAFSPTSFSHASIASTNVRSRTNLPPPPPPDRIPPPKGRPHQQKFKILRKGKDKDK
RQPTNSEDDWTIEDVIANGDGIEQEPQYLRDSQSEENESKPEQELVEITSREEKKEKASKSRELVRK
TSRLFGRDKDKDRDKCEEPAITGSSSSTLAATRQSSSTSTDSSTSRSFTSAFTRMNSIQSKRSPRRSF
GQAHSRRASQDSQTSWPAPRSIHSSITSHDPSIDPQDGADGNGVPIPQRQGASMSSLSRYSLPQP
SGNTSRSPDTFPNKMSTWFSHLLPVSSETPSPSTNESAFFVRKQPSVAASLFNAARQRAVDGVRHL
LDSEAQPDKCMDTIWVRGVAHPGWRPITPENGASNLPALEPGDGGVEDRRASSSMNRPSPTSFR
PSSWRRNTSLPPSAQLQPSTQIPSQASNQTTSPNKGFTGIWNPSTLSLAMSIGGSPNKEKEIGSGA
ESPSKKKGKEVVKWPEQFYDDFKSTVWFTYRNQYAPILSLSPDLLIPSPEAYYASFGPPLDATSPSSP
QVTIPTATAQQTATGGGGWSWSKEERGLTSDAGWGWRDWRLPSTPATFSKATTSQEIAALKDYAKY
AQMLSWFLDDPSPLCPFSVHRMALIGKELGKEVGEWFGPSTAAGTLKTLANSFAPGGVAVATATDS
IIYRSDVYAASNLPSDDWNRISPTLNPSRKKKRDNAEAKEGKWGERAVLILVGIRLGLDGVNPIYYD
SIKALFTFPQSVGIAGGRPSSSYYFVGSQANHLFYLDPHLTRPAIPLQIPPLPVHSAKEEGSMESSSIL
STAEEESEEGVMIHTPETPRSTTPSTFSAAEEVEDEEREEWEQGSKYKLDVMDADGFRVEEEISDDK
GRNEGKAIGSDIPKSTLESNGVIQERSDKQKTFTSTTSVDSQVDPQMLWYTTAYPDSLLRTYHCEKI
KKMPLSGLDPSMLLGFVCKSEDDFENFVERVAQLPKKIFTVQDEMPSWEEDDDAGLESVSEPDFE
EDEFEEPGTAKPRFDSSSPVNEDSLKGPSIVSASTTTTSIAAKEDYLDGEHGDGTDDDDESIGTTTAV
RPVDIVRHLHRVDLSVKREQEGGDDDDGEWVGGTPSSQGVLVEPPSLKGTPSKSRSSTAEPHHE
QGGEEEHERRVFPVRNRMESWVEPVCEGREAPNGDNLL

>tr|A0A9W4TUZ8|A0A9W4TUZ8_9ASCO Cysteine protease OS=Candida verbasci OX=1227364 GN=CANVERA_P0662 PE=3 SV=1

MNESTISNNNEDGQLKSNSKDNNNNIDSNLSLNNNYNKFTILLNQVINNNSSHSNSINSSLYILGN
KFINLIQANNYINNLIYFTYRCGFEPIPKTIDGPQPIQFFPSIIFNKSTLINFTNLKSLFDKENFTSDSGW
GCMIRTSQNLLANTLLKLNKNKDNMTEDIINLFQDSSSCKFSIHNFIKTASESPLQIKPGQWFGPNA
ASFSIKNLIELSQANDANNLLIPQVFISDNCDLYDDEITILLELKPLLLLFPVRLGIDQVNKYYHQSILQ
LLSTKHSVGISGGKPSSSFYFIGYEEQEEGNVELLYFDPHFPQVYENPINVNTYRTKNYNKLKIEDLD

PSMMIGILLNNVNDYLNFKKQCIETKNKILHFHPEFHNNDLTNQSWEEIQEVEDDFVNLNIRNDDE
EFIQL

>tr|A0AA97NUC8|A0AA97NUC8_PYRO3 Cysteine protease OS=Pyricularia oryzae (strain Y34) OX=1143189 GN=OOU_Y34scaffold00655g29 PE=3 SV=1

MTNNRCELIAQAPHISAPQGPGRGRPKITSAMEEPRPFPPKLLKTHFDFVGAVVWPIWFLINSGCP
ALPERARLVDSAQSFMDSAVAGAADIGRYGRRIVRMIWDPEPTNDPIANRPAWCLGYEYTLETNITS
KTKGEDSKLSTATSSDQQRPPAQANKVPQMPSAQLPTEAAATALSGNTTPPTPEAALEPTKITSQPAA
IDTPPDSVDSSFDSSMAYDDVPDDGGWPPAFLNDFESRIWMTYRSGFEPIPRSTDPTASSRMSFA
MRLKTMADQQAGFTTDSGWGCMIRTGQSLLANSLLTCRLGRSWRRGQAPDEERKLLSLFADDPR
APYSIHNFVAHGAAKCGKYPGEWFGPSATARCIHALANATENSFRVYSTGDLPDVYEDSFMEVAKP
DGKTFHPTLILISTRLGIDKINQVYWESLTATLQLPQSVGIAGGRPSSSHYFVGAQRSDEDQGSYLFY
LDPHHTRPALPFHEDPQLYTPSDVDSCHTRRLRRLHIREMDPSMLIGFLILDEENWHAWKSSVKH
VQGKSIITVSEHDPSKGSASGRPSAIDEVETLSDDDGDSVLDG

>tr|A0AAD4CIC9|A0AAD4CIC9_ASPNN Cysteine protease OS=Aspergillus nanangensis OX=2582783 GN=ATG4 PE=3 SV=1

MNSVDIGRCSKRIVQYLWDPEPRNDEEPHESIWCLGVEYPTNQRSTAALETTNSKSNPSMPLLLIH
KLLHEGFVNSQSEVDEVTADEPSAESRSRAPQQETLHGWPDDFLLDFESRIWMTYRSNFPPIPKL
GDRDANNMTLSVRLRSQLMDPEGFTSDTGWGCMIRSGQCLLANAMSSLLLGRDWRRGKDTTEE
TRLLSLFADHKDAPLSIHRFVKHGAECCGKHPGEWFGPSATARCIEALSSQYGNPTLRVYVSNDSS
DVYQDKFMNVARDESGSIQPTLILLGTRLGIDHITPVYWDSLKATLRFPQSVGIAGGRPSASHYFVG
VQGSFFFYLDPHNTRSALPPRPTGDPYSKDDVDSYHTRRLRRIHIKDMDPSMLIGILIKDEADWWD
WKKRVGTTDGKPIIHVLNEMQRSPQGREEALDEVEALDDAE

>tr|A0AAD5BAE1|A0AAD5BAE1_9ASCO Cysteine protease OS=Candida theae OX=1198502 GN=KGF57_005259 PE=3 SV=1

MSELQSIAEPQEVGETTATTSSLTDSTTTTTTGFDRFTSFLKGIPGINLGFEEANECTEPTTKNNSAESR
EVVVLGRHFTIQDKANDYIHSLLWLSYRCGFTPIPKVVDGPQPVTFFPSLLFSKSTLNNVGNLRSLF
DNENFTSDAGWGCMIRTSQNLLANALLKLKNEPDETAQLEILKLFQDNPSSAFSIHNFIRVASNSPL
SVKPGQWFGPNAASYSIRQLVADYTGQASSTKVPCVYISENADLYDDEVDDLFVKEKKPLLLLFPVR
LGIDQVNKYYYRSILQLLASKFSVGIAGGKPSSSFYFIGYENEQSLIYFDPHLPQVVETPLNLASYHTS
NYNKLDITLLDPSMMVGVLLESMTDYKEFKQESIENKIIHFHPLVSTSQQDLTLNQSWEEVQEDDD
FVNLTVPKSEEEFIVLEE

>tr|A0AAI9WYB8|A0AAI9WYB8_9ASCO Cysteine protease OS=Candida oxycetoniae OX=497107 GN=KGF56_002381 PE=3 SV=1

MQREYSVGWYKTQEGFAYKKMDQDESNKNDVVVVLGSRDISEEEPREVVDTPEIPFVGRNLGRNL
ERFSAFMRDISGINGQEPSGNEQADNEKVSNEQAGNEQASNRSFCELPSQSSSARNNFVVLGKE
FDNAVDAKDRVSSLLWMSYRCGFEPIPKSEDGPKPIFFFSSIVFNKQTLNNLKNLKSLLDNENFTSD
AGWGCMIRTSQNLLANTMLKLAKSKQEEKKEKQNKAEDDKKQNEVDDKKQNEVDDKKQRDKEIF
SQIIALFQDNIKSPFSLHNFIRVVSGSPLQIKPGQWFGPNAASASIKKLTAEQNEKNDADSLVPVPFV
YVSENCDLYDDEIEEIFQKEKKPLLLLFPIRLGIDQVNKYYHDSILQLLASEYSVGIAGGKPSSSFYFL
GYENDDELIYLDPHSPQMVEEPINTNSYHVTEYSKLNIEMLDPSMMIGILVKTMAEYQSFKLECTSS
KNKILHFHPLVTVVPQESSITQSWEEVQGGEEEDFVNLNVKKNEEEFIDLGK

>tr|A0AAN4PDS8|A0AAN4PDS8_9EURO Cysteine protease OS=Aspergillus lentulus OX=293939 GN=ALT_2210 PE=3 SV=1

MNGVDMERCKRIIQYIWDPAPRNDQPGDPIWCLGTKYTTDGMERANRRTAAPKSIRLANEEGPN
QLVPKATTPGQLDEQGWPESFLHDFESRIWITYRSNFPPIPKPINQDAFSAMTLSVRLRSQLVDQH
GFTSDTGWGCMIRSGQSLLANAMSILLFGRGWRRGIDTDGEAQLLLQFADHPDAPFSIHRFVQH
GAESCNKHPGEWFGPSATARCIQALVSQHGSLNLGVYMTDDTADVHEDKFLDAAHDERGSFRPT
LILIGTRLGIDHITPVYWDAVKTTLQLPQSVGIAGGRPSASHYFVGVQGSHLFYLDPHQTRPALPRRN
IDEPYTDEEIETYHTRRLRRIHIRDMDPSMLIGFIIKDREDWAQWKSGVSVQGKPIVQVHSESDTAVF
HGREGAIDEVEVLDDD

>tr|A0AAN4YNW1|A0AAN4YNW1_ASPOZ Cysteine protease OS=Aspergillus oryzae OX=5062 GN=Aory04_000744900 PE=3 SV=1

MNSVDIGRCRKRIVQYIWDPEPRNDEEPDASIWCLGVEYAPQPQKITANTTPGKLDDVTAHGWPE
AFVSDFESKIWMTYRSDFPPIPRLDNDEANHPMTLTVRIRTQLMDPQGFTSDTGWGCMIRSGQSL
LANAMLTLCLGRDWRRGDKAEEEARLLSLFADHPDAPLSIHRFVKYGAESCGKHPGEWFGPSATA
RCIEYAPNTSTPPRARDLPANRALSAQCGNIAPRVYVTNDTSDVYEDSFLRVARSGSGSIQPTLILLG
TRLGIDNVTPVYWDGLKAVLQLPQSVGIAGYVYITSAWQ

>tr|A0AAN7DM20|A0AAN7DM20_9FUNG Cysteine protease OS=Mucor velutinosus OX=708070 GN=ATC70_009320 PE=3 SV=1

MTHHSNEQKHVDKELPQPHNSNHISTAPLKDKLLTNSSYLAAEIPLKLGHFMSNLWISGSELSNSF
FLQNGECNPDSETAQIIWLLGRPYQTKPLDQMQQAILDAQKDEMFRPGGTTADRDDGEVLKDNV
SMVWPPDFYDDFTSRLWMTYRHNYPPIRPSNHKTDIGWGCMLRSGQSLLANTLLIHFLSRDWRR
QKQTAAARLQYAKIIHWFLDELSPRAPFSIHRIALLGKQLGKNIGEWFGPSTISQVIQALVSDFQPAD
LSVYIATDGTIYLDGVQDVTTGKKPRGDFSYFTNKLSSSASDEGREEAKHLYDASSTPQEQEQATSP
NTDSPDDKDTVFKPVLMLVALRLGIDSLHTTYYPALKACFELPSFVGIAGGRPNSSLYFIGLQGDDLI
YLDPHFSRPALETKSLSEYTREDFSTYHCTIPRRIPIANLDPSMMLGFYCRTQRELDLFCDQIKTISQK
HTAIFSIQQSTPEYDEDVRSENDFGVMSDEDDPLENKSKCKEDDCSNDSDSNSIF

>tr|B5VQI7|B5VQI7_YEAS6 Cysteine protease OS=Saccharomyces cerevisiae (strain AWRI1631) OX=545124 GN=AWRI1631_141070 PE=3 SV=1

MDDFLSRIGVIYMQRWLQLWKMDLVQKVSHGVFEGSSEEPAALMNHDYIVLGEVYPERDEESGA
EQCEQDCRYRGEAVSDGFLSSLFGREISSYTKEFLLDVQSRVNFTYRTRFVPIARAPDGPSPLSLNL
LVRTNPISTIEDYIANPDCFNTDIGWGCMIRTGQSLLGNALQILHLGRDFRVNGNESLERESKFVN
WFNDTPEAPFSLHNFVSAGTELSDKRPGEWFGPAATARSIQSLIYGFPECGIDDCIVSVSSGDIYEN
EVEKVFAENPNSRILFLLGVKLGINAVNESYRESICGILSSTQSVGIAGGRPSSSLYFFGYQGNEFLHF
DPHIPQPAVEDSFVESCHTSKFGKLQLSEMDPRCSLVF

>tr|B9WLC3|B9WLC3_CANDC Cysteine protease OS=Candida dubliniensis (strain CD36 / ATCC MYA-646 / CBS 7987 / NCPF 3949 / NRRL Y-17841) OX=573826 GN=Cd36_28350 PE=3 SV=1

MDQQNLNNQPDVEASPEQTSNEEVDTVLGRFTLFVKDLSNGLNGSQEVPTSQESASEEAELINRK
IVILGQIFDNSNAANNYIESKLWLSYRCGFEPIPKSIDGPQPIHFFPSIIFNRTTIYSNFANLKSLFDKE
NFTSDAGWGCMIRTSQNLLANTLLKLYPKNEQEIVKLFQDDTKSPFSIHNFIRVASSSPLHVKPGEW
FGPNAASLSIKRLTNELQDQEINGINPPRVFISENSDLFDDEIRDVFAKEKSNSVIILFPIRLGIDKVNS
YYYNSIFHLLSSKYSCGIAGGKPSSSFYFLGYEDTDLIYFDPHLPQIVETPFNMDSYHSTNYNTLNISL
LDPSMMIGILVTNIDEYIDFKTSCIDNNNKIVHFHPHTLPVQQDSIINQSWEEVQDEEEEFVNLNVS
KIENEQQQEEVTDTPDEFIDIGNQSSSVVSVPSNV

>tr|C4YLD9|C4YLD9_CANAW Cysteine protease OS=Candida albicans (strain WO-1) OX=294748 GN=CAWG_01657 PE=3 SV=1

MNQPNPNKQPIVQSTSEQTSNEEVDTVLGRFTLFVKDLSNGLNGSQEVPPSQESVSEEAEVISRKII
VLGQTFDNFDTANDYIESKLWLSYRCGFEPIPKSIDGPQPIQFFPSIIFNRSTIYSNFANLKSLFDKEN
FTSDAGWGCMIRTSQNLLANTLLKLYPKNEPEIVKLFQDGTSSPFSIHNFIRVASLSPLHVKSGEWF

GPNAASLSIKRLTSELLQDQEIDGIKIPRVFISENSDLFDDEIRDVFAKEKNASVLILFPIRLGIDKVNSY
YYNSIFHLLASKYSCGIAGGKPSSSFYFLGYEDTDLIYFDPHLPQVVETPINMDSYHTTNYNRLNISLL
DPSMMIGILVTNIDEYIDFKTSCLDNNNKIVHFHPHTLPVQQDSIINQSWEEVQDEEEEFINLNVSKI
ENEHQQEQEQLTDAPDEFIDIGNQSSSVVSVPSNV

>tr|C5P2M5|C5P2M5_COCP7 Cysteine protease OS=Coccidioides posadasii (strain C735) OX=222929 GN=CPC735_038270 PE=3 SV=1

MNTVDIGQKYKRIVEYLWDPEPKNDDDIIEPVWCLGKEYKTSIPRDSEGAEAPESCNMPGMPFLSP
MNQMSLSSRDTQAALSKPATPPHQLGIQRSKSREWPTSFLDDFESKFWFTYRSNFPAIPKSRDPDT
PLALTLSVRLRSQFLDTHGFTADTGWGCMIRSGQSLLANALSILNLGRDWRRGSKIKEECELLSLFA
DNPQAPFSIHRFVDYGASACGKHPGEWFGPSATARCIEALSNECKHTDLNVYVMSDGSDVHEDQ
FRQIAGPDGIRPTLILLGVRLGIESVTPVYWEALRAIIRYPQSVGIAGGRPSSSLYFIGVQGPYFFYLDP
HHTRPAVSWNPDSTLSPENLDTYHTRRLRRLHIREMDPSMLIGFLIKDDDDWKDWKRRLRSVTGN
PIIHIFDLERPNFGRHLEREEAVDEVEALDDDSN

>tr|C7GPD4|C7GPD4_YEAS2 Cysteine protease OS=Saccharomyces cerevisiae (strain JAY291) OX=574961 GN=ATG4 PE=3 SV=1

MQRWLQLWKMDLVQKVSHGVFEGSSEEPVALMNHDYIVLGEVYPERDEESGAEQCEQDCRYRG
EAVSDGFLSSLFGREISSYTKEFLLDVQSRVNFTYRTRFVPIARAPDGPSPLSLNLLVRTNPISTIEDYI
ANPDCFNTDIGWGCMIRTGQSLLGNALQILHLGRDFRVNGNESLERESKFVNWFNDTPEAPFSL
HNFVSAGTELSDKRPGEWFGPAATARSIQSLIYGFPECGIDDCIVSVSSGDIYENEVEKVFAENPNS
RILFLLGVKLGINAVNESYRESICGILSSTQSVGIAGGRPSSSLYFFGYQGNEFLHFDPHIPQPAVEDS
FVESCHTSKFGKLQLSEMDPSMLIGILIKGEKDWQQWKLEVAESAIINVLAKRMDDFDVSCSMDD
VESVSSNSMKKDASNNENLGVLEGDYVDIGAIFPHTTNTEDVDEYDCFQDIHCKKQKIVVMGNTH
TVNANLTDYEVEGVLVEKETVGIHSPIDEKC

>tr|C8ZG10|C8ZG10_YEAS8 Cysteine protease OS=Saccharomyces cerevisiae (strain Lalvin EC1118 / Prise de mousse) OX=643680 GN=EC1118_1N9_1211g PE=3 SV=1

MDDFLSRIGVIYMQRWLQLWKMDLVQKVSHGVFEGSTEEPVALMNHDYIVLGEVYPERDEESGAE
QCEQDCRYRGEAVSGGFLSSLFGREISSYTKEFLLDVQSRVNFTYRTRFVPIARAPDGPSPLSLNLL
VRTNPISTIEDYIANPDCFNTDIGWGCMIRTGQSLLGNALQILHLGRDFRVNGNESLERESKFVNW
FNDTPEAPFSLHNFVSAGTELSDKRPGEWFGPAATARSIQSLIYGFPECGIDDCIVSVSSGDIYENEV
EKVFAENPNSRILFLLGVKLGINAVNESYRESICGILSSTQSVGIAGGRPSSSLYFFGYQGNEFLHFD
PHIPQPAVEDSFVESCHTSKFGKLQLSEMDPSMLIGILIKGEKDWQQWKLEVAESAIINVLAKRMD

DFDVSCSMDDVESVSSNSMKKDASNNENLGVLEGDYVDIGAIFPHTTNTEDVDEYDCFQDIHCKK
QKIVVMGNTHTVNANLTDYEVEGVLVEKETVGIHSPIDEKC

>tr|E6RDM5|E6RDM5_CRYGW Autophagy-related protein 4 OS=Cryptococcus gattii serotype B (strain WM276 / ATCC MYA-4071) OX=367775 GN=CGB_K2500C PE=3 SV=1

MSSPTPTSPTSSLAFSPTSFSHASIASTNVRSRTNLPHPHPPDRIPPPKGRPHQQKFKILRKGKDKDK
RQPTNSEDDWTIEDVIANGDGIEQGPQYLDDLQWEENESKPERQLVEMASREEKKEKASKSRELV
RKTSRLFGRDKDKDRDKFEEPAVTGSSSSTLAATRQSSSTSTDSSTSRSFTSAFTRMNSIQSKRSPRR
SFGQAHSRRASQDSQMSWPAPRSIHSSITSHDPSSDPQDGAGGNGVPIPQRQGASMSSLSRYSL
PQPNGNTSRSPDTFPNKMSTWFSHLLPVSSETPSPSTNETASSVRKQPSVAASLFNAARQKAVDG
VRHLLDSEAQPDKCMDTIWVRGVAHPGWRPITPENGASNLPVLEPGDGGVEDRTASSSMNRPSP
TSFRPSSWRRNTSLPPSAQLQPSTQIHSQASNQTTSPNKGFTGIWNPSTLSLAMSIGGSPNKEKEIG
SGAESPSKKKGKEVVKWPEQFYDDFKSTVWFTYRNQYAPILSLSPDLLIPSPEAYYASFGPPLDATSP
SSPQVTTPTATAQQTATGGGGWSWSKEERGLTSDAGWGCMLRTGQSLLINALIHVHLGRDWRLP
STPATFSEATTSQEIAALKDYAKYAQMVSWFLDDPSPLCPFSVHRMALIGKELGKEVGEWFGPSTAA
GTLKTLANSFAPCGIAVATATDSIIYRSDVYAASNLPSDDWNRISPTFNPSRKKKRHNAEAKEGKWG
ERAVLILVGIRLGLDGVNPIYYDSIKALFTFPQAGGSAGGRPSSSYYFVGSQANHLFYLDPHLTRPAIP
LQVPPLPVHSAKEEGSMESSSILSTAEEESEEGVMVHTPETPRSTTPSTFSAAEEVEDEEREEWEQG
SKYKLDVMNADGFRVEEEISDDKGRNEGKAIGSDIPKSTLESNGVIQERSDKQKTFTSTASVDSQID
PQMLWYTTAYPDSLLRTYHCEKIKKMPLSGLDPSMLLGFVCKSEDDFENFVERVALLPKKIFTVQDE
MPSWEEDDDAGLESVSEPDFEEDEFEEPGTAKPRFDSSSPVNEDSLKGPSIVSASTTATPIAAKEDY
LDGEDGDGTDDDDESIDTTTAVRPVDIVRHLHRVDLSVKREQEGGDDDDGEWVGGTPSSQGVLV
EPPSLKGTPSKSRSSTAEPHHEQSGEEEQERRVFPVRNRMESWVEPVCEGKEAPNGDNLL

>tr|E9CTA7|E9CTA7_COCPS Cysteine protease OS=Coccidioides posadasii (strain RMSCC 757 / Silveira) OX=443226 GN=CPSG_00005 PE=3 SV=1

MNTVDIGQKYKRIVEYLWDPEPKNDDDIIEPVWCLGKEYKTSIPRDSEGAEAPESCNMPGMPFLSP
MNQMSLSSRDTQAALSKPATPPHQLGIQRSKSREWPTSFLDDFESKFWFTYRSNFPAIPKSRDPDT
PLALTLSVRLRSQFLDTHGFTADTGWGCMIRSGQSLLANALSILNLGRDWRRGSKIKEECELLSLFA
DNPQAPFSIHRFVDYGASACGKHPGEWFGPSATARCIEALSNECKHTDLNVYVMSDGSDVHEDQ
FRQIAGPDGIRPTLILLGVRLGIESVTPVYWEALRAIIRYPQSVGIAGGRPSSSLYFIGVQGPYFFYLDP
HHTRPAVSWNPDSTLSPENLDTYHTRRLRRLHIREMDPSMLIGFLIKDDDDWKDWKRRLRSVTGN
PIIHIFDLERPNFGRHLEREEAVDEVEALDDDSN

>tr|G2WLP0|G2WLP0_YEASK Cysteine protease OS=Saccharomyces cerevisiae (strain Kyokai no. 7 / NBRC 101557) OX=721032 GN=K7_ATG4 PE=3 SV=1

MQKWLQLWKMDLVQKVSHGVFEGSSEEPVALMNHDYIVLGEVYPEREEESGAEQCEQDCRYRG
EAVSGGFLSSLFGREISSYTKEFLLDVQSRVNFTYRTRFVPIARAPDGPSPLSLNLLVRTNPISTIEDYI
ANPDCFNTDIGWGCMIRTGQSLLGNALQILHLGRDFRVNGNESLERESKFVNWFNDTPEAPFSL
HNFVSAGTELSDKRPGEWFGPAATARSIQSLIYGFPECGIDDCIVSVSSGDIYENEVEKVFAENPNS
RILFLLGVKLGINAVNESYRESICGILSSTQSVGIAGGRPSSSLYFFGYQGNEFLHFDPHIPQPAVEDS
FVESCHTSKFGKLQLSEMDPSMLIGILIKGEKDWQQWKLEVAESAIINVLAKRMDDFDVSCSMDD
VESVSSNSMKKDASNNENLGVLEGDYVDIGAIFPHTTNTEDVDEYDCFQDIHCKKQKIVVMGNAH
TVNANLTDYEVEGVLVEKETVGIHSPIDEKC

>tr|G3B7X9|G3B7X9_CANTC Cysteine protease OS=Candida tenuis (strain ATCC 10573 / BCRC 21748 / CBS 615 / JCM 9827 / NBRC 10315 / NRRL Y-1498 / VKM Y-70) OX=590646 GN=CANTEDRAFT_115142 PE=3 SV=1

MASMSTKDIPEQHSLETPLEDAPPERSGLSFSFSELWQHIPRPTSNPGNETEVIVEKPPKIIMGQSYK
VEDEFLLQVEALVWLTYRTGFEPIPKNPNGPHPLAFVQSMVFNKNPLSTNVHSFIDNENFTTDVGW
GCMIRTSQSLLANTYKRMISEDAQQEIQLLDQFKDSEAAPFSLHNFIRVANESPLQVKPGQWFGP
NAASLSIQRLCNLVNSKENFGLPGLSVLISENSDLYDDKVQEFLDKKKQSLLILLPIRLGIDKTNEFYY
SSILQLLNCKQSVGIAGGKPSSSFYFFGYDNDELLYLDPHYPQGTNAGYNSYHTPRYQRLTISQLDP
SMMIGILVDDLQDYNTFKAECLEKNNKIVHFAAKSAINEIQAPASEVNADFVDFVDDFDEDDIVEDE
DLDRVS

>tr|G3YC95|G3YC95_ASPNA Cysteine protease OS=Aspergillus niger (strain ATCC 1015 / CBS 113.46 / FGSC A1144 / LSHB Ac4 / NCTC 3858a / NRRL 328 / USDA 3528.7) OX=380704 GN=ASPNIDRAFT_178675 PE=3 SV=1

MNTVDIGRCSKRIVQYLWDPEPRNDEDPNSSIWCLGIEYHPDKDANTRETQHAWPESFLLDFESR
IWMTYRSNFPPIPRVEGDDKSASMTLGVRLRSQLVDTQGFTSDTGWGCMIRSGQSLLANALSMLV
LGRDWRRGARFEEESQLLSLFADTPTAPFSVHRFVKHGAESCGKYPGEWFGPSATAKCIEALSSQC
GNPTLKVYVSNDTSEVYQDKFMDIARNTSGAFQPTLILLGTRLGIDNITPVYWDGLKAALQFPQSVGI
AGGRPSASHYFVGAQGSHLFYLDPHYTRPALPDRQEGELYSKEEVDTYHTRRLRRIHVRDMDPSM
LIGFLIRNQEDWADWLKRIEAVKGRPIIHVLKQMNPDHDQEAGALDQVEALDDIE

>tr|H0GMB2|H0GMB2_SACCK Cysteine protease OS=Saccharomyces cerevisiae x Saccharomyces kudriavzevii (strain VIN7) OX=1095631 GN=VIN7_4048 PE=3 SV=1

MQRWLQLWKMDLVQKVSHGVFEGSSEEPVALMNHDYIVLGEVYPERDEESGAEQCEQDCRYRG
EAVSGGFLSSLFGREISSYTKEFLLDVQSRVNFTYRTRFVPIARAPDGPSPLSLNLLVRTNPISTIEDYI
ANPDCFNTDIGWGCMIRTGQSLLGNALQILHLGRDFRVNGNESLERESKFVNWFNDTPEAPFSL
HNFVSAGTELSDKRPGEWFGPAATARSIQSLIYGFPECGIDDCIVSVSSGDIYENEVEKVFAENPNS
RILFLLGVKLGINAVNESYRESICGILSSTQSVGIAGGRPSSSLYFFGYQGNEFLHFDPHIPQPAVEDS
FVESCHTSKFGKLQLSEMDPSMLIGILIKGEKDWQQWKLEVAESAIINVLAKRMDDFDVSCSMDD
VESVSSNSMKKDASNNENLGVLEGDYVDIGAIFPHTTNTEDVDEYDCFQDIHCKKQKIVVMGNTH
TVNANLTDYEVEGVLVEKETVGIHSPIDEKC

>tr|H0GZX6|H0GZX6_SACCK Cysteine protease OS=Saccharomyces cerevisiae x Saccharomyces kudriavzevii (strain VIN7) OX=1095631 GN=VIN7_9465 PE=3 SV=1

MDLVQKMSHGVLPGSSEESVAFTNHDYVVLGEMYPEQEQKNSYQQSEQEDEFSSEAASGGFLSN
LFGRENSSYTQEFLLDVRSRVNFTYRTRFVPIARAPDGPSPLSLNVLVRTNPINTIENYIANPDCFNT
DIGWGCMIRTGQSLLGNALQILHLGRDFRVDEDDDFRRESRIVNWFNDTPEAPFSLHNFVSTGTE
LSDKRPGEWFGPAATARSIQYLIYGFPECGINACIVSVSSGDIYENEVEEVFVDNPNSSILFLLGVKL
GINAVNESYRESICGILNSAWSVGIAGGRPSSSLYFFGYQGNEFLHFDPHIPQPAVEDSFVNSCHTS
KFGRLQLSEMDPSMLIGVLIKGEKDWQRWKLEIAESTIIKVLPERMDDFDVSCSMDDVESVDSSSM
KKDISNNENLGVLEGDYVDIGAILPHTADTEDAEEDDCFQDIHCKNQKIVVMGNTHAVNANLTDYE
VEGVLVEKETVGIPSPINEKC

>tr|H8WW02|H8WW02_CANO9 Cysteine protease OS=Candida orthopsilosis (strain 90-125) OX=1136231 GN=CORT_0A02350 PE=3 SV=1

MVDSTPSSEQQETKYSVPTQPQSTDATTGGGFERLTSFFKGISGLNMGFEEATESNEPSGNIIESKPI
VILGNHFINQDEARDHIYSLLWLSYRCGFSPIPKSIDGPQPVTFFPSLLFSKSTLTNVGNLRSLFDNE
NFTSDAGWGCMIRTSQNLLANALLKLAGEANGNVQLEILKLFQDDPNAAFSIHNFIRVASASPLSV
KPGQWFGPNAASISIRQLTIEMTDQESPTVVPFVYISENADLYDDEIEETFLKEKRPLLLLFPVRLGID
HVNKYYYKSILQLLASRFSVGIAGGKPSSSFYFIGYENDENLIYFDPHLPQVFESPINLASYHTLNYN
KLSIEMLDPSMMIGVLLGSMSEYRELKQECTENKIIHFHPLVTSQQDSTLNQSWEEVQEDDDFVNL
TIPKSEEEFVVLNE

>tr|I8TUW6|I8TUW6_ASPO3 Cysteine protease OS=Aspergillus oryzae (strain 3.042) OX=1160506 GN=Ao3042_05982 PE=3 SV=1

MNSVDIGRCRKRIVQYIWDPEPRNDEEPDASIWCLGVEYAPQPQKITANTTPDNDEANHPMTLTVR
IRTQLMDPQGFTSDTGWGCMIRSGQSLLANAMLTLCLGRDWRRGDKAEEEARLLSLFADHPDAP

LSIHRFVKYGAESCGKHPGEWFGPSATARCIEALSAQCGNIAPRVYYVTNDTSDVYEDSFLRVARSG
SGSIQPTLILLGTRLGIDNVTPVYWDGLKAVLQLPQSVGIAGGRPSASHYFIGTQGPYFFYLDPHTTR
PAVPYSIDGRLLSKTEISTYHTRRLRRIHIQDMDPSMLIGFLVRNEDDWEDWKGRVGSVVGKQIIHV
FKGEEATYNQGRRGALDEVEALDDAE

>tr|J9VN57|J9VN57_CRYNH Autophagy-related protein 4 OS=Cryptococcus neoformans var. grubii serotype A (strain H99 / ATCC 208821 / CBS 10515 / FGSC 9487) OX=235443 GN=CNAG_02662 PE=3 SV=1

MSSPASTSPKSSFTFSPTSFPHASIASTNVRPRTNLPPPPPPDRIPPPKGRSHQQKFKILRKGKDKDK
RQPTDLEDDWTIEDVTVNNGNVEQEPQYLDDVQPEENELKPEPRLVEMANREEKKEKTFKSRELV
KKTSRLFGRDKDKDRDKSEEPAGSSSSTLAAMRQSSSTSTDSTTSRSITSAFTRQNSIQSRRSPRTSF
GQAHSRRTSQDSQMSWPAPRSIRSSITPHDPPNDPQNSANNTGVPIPQRQGASMSSLSRYSLPQ
PNGNASRSPDTFPNKMSTWFSHLLPVSSESPPSSNYETSPSVRKQPSVAASLFNAARQKAVDGVR
HLLDSEAQPDKCMDTIWVRGVAHPGWRPITPENGTSNLPTIEPGGGGGGVEDRRASLSMNRPSP
TSLRPSSWKRNTALPPTAQPQPSTQQHTQASNQTTSPSKGFTGIWNSSTLSLGMPIGGSPNKERES
GSGAESPSKKKNKEIVKWPEQFYDDFRSTVWCTYRSQYAPISSLSPNLLIPSPEAYYASFGPPLDATS
PSSPRVTIPTTAAQQTASGSGGWGWSKEERGLTSDAGWGCMLRTGQSLLVNALIHVHLGRDWRV
PSTPASFSEATTNQETAALKDYAKYAQMLSWFLDDPSPLCPFSVHRMALIGKELGKEVGEWFGPST
AAGALKTLANSFAPCGVAVATATDSIIYKSDVYTASNLPSDDWNSISPTFNSSKKKRGGDNKAKEGK
WGKRAVLILVGIRLGLDGVNPIYYDSIKALFTFPQSVGIAGGRPSSSYYFIGSQANHLFYLDPHLTRPA
IPLQVPPLPVHSAKEEGSMENSSILSTTEEESEEGVMIRTPETPRSTTPSTFSAPEQIEDEGQEEWEQ
GSKYKLDVVDADGAKVEEIDDDKGWNEGETIKEEIPKNAFESKGVVQDHSEKQRGSASTASVDSQ
VDPQMLWYTTAYPDSLLRTYHCEKIKKMPLSGLDPSMLLGFVCKDEDDFEDFVERVAQLPKKIFTV
QDEMPSWEEDDDAGLESVSEPDFEGDELEEPGTAKPRFDSSSPVNEDSLKGPRVVSASTTATPLA
AKEGHLDVEETNSTDDDDESIGTTTAVRPVDIARYLHRVDLSSKREQEDDDDGEWVGGTPSSQGV
LVEPPSLKGTPSKSRSSALEPRHEQNGEKEQERHVFPARNRMESWVEPVCEGKEAPNGDNLL

>tr|L7IVU7|L7IVU7_PYRO1 Cysteine protease OS=Pyricularia oryzae (strain P131) OX=1143193 GN=OOW_P131scaffold01337g51 PE=3 SV=1

MTNNRCELIAQAPHISAPQGPGRGRPKITSAMEEPRPFPPKLLKTHFDFVGAVVWPIWFLINSGCP
ALPERARLVDSAQSFMDSAVAGAADIGRYGRRIVRMIWDPEPTNDPIANRPAWCLGYEYTLETNITS
KTKGEDSKLSTATSSDQQRPPAQANKVPQMPSAQLPTEAAATALSGNTTPPTPEAALEPTKITSQPAA
IDTPPDSVDSSFDSSMAYDDVPDDGGWPPAFLNDFESRIWMTYRSGFEPIPRSTDPTASSRMSFA
MRLKTMADQQAGFTTDSGWGCMIRTGQSLLANSLLTCRLGRSWRRGQAPDEERKLLSLFADDPR
APYSIHNFVAHGAAKCGKYPGEWFGPSATARCIHALANATENSFRVYSTGDLPDVYEDSFMEVAKS

DGKTFHPTLILISTRLGIDKINQVYWESLTATLQLPQSVGIAGGRPSSSHYFVGAQRSDEDQGSYLFY
LDPHHTRPALPFHEDPQLYTPSDVDSCHTRRLRRLHIREMDPSMLIGFLILDEENWHAWKSSVKH
VQGKSIITVSEHDPSKGSASGRPSAIDEVETLSDDDGDSVLDG

>tr|M3HMH6|M3HMH6_CANMX Cysteine protease OS=Candida maltosa (strain Xu316)
OX=1245528 GN=G210_0775 PE=3 SV=1

MDTNTSDPEPSPAMTESQESNEEIDTVLGRFTLFVKDLSNSLNAGSRDDTPPSNDPEPPQTKELVIL
GERFSNEHDANEFIRSKLWLSYRCGFDPIPKSETGPQPIQFFPSIVFNRTTISSNFANLKGLFDKDNF
TSDAGWGCMIRTSQNLLANTLLKLLPDSEKEIIDLFQDTPESCFSIHNFIRVANESPLEVKPGQWFG
PNAASLSIKRLCNGLEDSEIQGVKYPKVFISENCDLYDDEIKELLNEEGRSVLLLLPIRLGIDKVNVYY
YNSILQFLSSKFSVGISGGKPSSSFYFLGFEESELLYFDPHLPQLVETPINLESYHTTTCNKLNISLLDP
SMMIGLLMQSMDEYLEFKTACIDNDNKIVHFHPHISPTQQQDSIINQSWENVQDEDDEDFVNLN
VNKYEDEEEDQQKQDEFIDLGK

>tr|N1NX11|N1NX11_YEASC Cysteine protease OS=Saccharomyces cerevisiae (strain
CEN.PK113-7D) OX=889517 GN=CENPK1137D_2816 PE=3 SV=1

MQRWLQLWKMDLVQKVSHGVFEGSSEEPAALMNHDYIVLGEVYPERDEESGAEQCEQDCRYRG
EAVSDGFLSSLFGREISSYTKEFLLDVQSRVNFTYRTRFVPIARAPDGPSPLSLNLLVRTNPISTIEDYI
ANPDCFNTDIGWGCMIRTGQSLLGNALQILHLGRDFRVNGNESLERESKFVNWFNDTPEAPFSL
HNFVSAGTELSDKRPGEWFGPAATARSIQSLIYGFPECGIDDCIVSVSSGDIYENEVEKVFAENPNS
RILFLLGVKLGINAVNESYRESICGILSSTQSVGIAGGRPSSSLYFFGYQGNEFLHFDPHIPQPAVEDS
FVESCHTSKFGKLQLSEMDPSMLIGILIKGEKDWQQWKLEVAESAIINVLAKRMDDFDVSCSMDD
VESVSSNSMKKDASNNENLGVLEGDYVDIGAIFPHTTNTEDVDEYDCFQDIHCKKQKIVVMGNTH
TVNANLTDYEVEGVLVEKETVGIHSPIDEKC

>tr|S2JPG2|S2JPG2_MUCC1 Cysteine protease OS=Mucor circinelloides f. circinelloides
(strain 1006PhL) OX=1220926 GN=HMPREF1544_01337 PE=3 SV=1

MTHHSNEQKHVDKELPQPHNSNHISTAPLKDKLLTNSSYLAAEIPLKLGHFMSNLWISGSELSNSF
FLQNGECNPDSENAQIIWLLGRPYQSKPLDQMQQAILDAQKDEMFRPGADREEEEVLKDNVSMV
WPPDFYDDFTSRLWMTYRHNYPPIRPSNHKTDIGWGCMLRSGQSLLANTLLIHFLSRDWRRQKQ
TAAARQQYGKIIHWFLDELSPRAPFSIHRIALLGKQLGKNIGEWFGPSTISQVIQALVSDFQPADLSV
YIATDGTIYLDSLQDVTTGKKPRGDFSYLTNKLSSSSSDEGREEAKNLYDASSTREEQEQTTSPNADS
SDDKETVFKPVLMLVALRLGIDSLHPTYYPALKACFELPSFVGIAGGRPNSSLYFIGLHGDDLIYLDP
HFSRPALETKSLSEYTKEDFSTYHCTIPRRIPIANLDPSMMLGFYCRTQRELDLFCDQIKTISQKYTAIF
SIQQSAPEYDEDVRSENDFGVMSDEDDPLQDDCKCKDDNSDSDSNSIF

Supplemental Data 2. Sequences of ATG8 proteins of major human fungal pathogens used for Figure S1A.

Green highlighted sequences are the signature sequences of ATG8 that made a cleavage at Glycine for further process.

Yellow highlighted sequences are annotated as ATG8 protein with no consensus sequences. Therefore it was removed from the tree.

>1 sp|Q9BXW4|MLP3C_HUMAN Microtubule-associated proteins 1A/1B light chain 3C OS=Homo sapiens OX=9606 GN=MAP1LC3C PE=1 SV=1

MPPPQKIPSVRPFKQRKSLAIRQEEVAGIRAKFPNKIPVVVERYPRETFLPPLDKTKFLVPQELTMTQFLSI
IRSRMVLRATEAFYLLVNNKSLVSMSATMAEIYRDYKDEDGFVYMTYASQETFGCLESAAPRDGSSLED
RPCNPL

>2 sp|Q9GZQ8|MLP3B_HUMAN Microtubule-associated proteins 1A/1B light chain 3B OS=Homo sapiens OX=9606 GN=MAP1LC3B PE=1 SV=3

MPSEKTFKQRRTFEQRVEDVRLIREQHPTKIPVIIERYKGEKQLPVLDKTKFLVPDHVNMSELIKIIRRRLQ
LNANQAFFLLVNGHSMVSVSTPISEVYESEKDEDGFLYMVYASQETFGMKLSV

>3 sp|Q9H492|MLP3A_HUMAN Microtubule-associated proteins 1A/1B light chain 3A OS=Homo sapiens OX=9606 GN=MAP1LC3A PE=1 SV=2

MPSDRPFKQRRSFADRCKEVQQIRDQHPSKIPVIIERYKGEKQLPVLDKTKFLVPDHVNMSELVKIIRRR
LQLNPTQAFFLLVNQHSMVSVSTPIADIYEQEKDEDGFLYMVYASQETFGF

>4 sp|Q9H492-2|MLP3A_HUMAN Isoform 2 of Microtubule-associated proteins 1A/1B light chain 3A OS=Homo sapiens OX=9606 GN=MAP1LC3A

MKMRFFSSPCGKAAVDPADRCKEVQQIRDQHPSKIPVIIERYKGEKQLPVLDKTKFLVPDHVNMSELVK
IIRRRLQLNPTQAFFLLVNQHSMVSVSTPIADIYEQEKDEDGFLYMVYASQETFGF

>5 sp|Q8LEM4|ATG8A_ARATH Autophagy-related protein 8a OS=Arabidopsis thaliana OX=3702 GN=ATG8A PE=1 SV=2 (#006633)
MAKSSFKISNPLEARMSESSRIREKYPDRIPVIVEKAGQSDVPDIDKKKYLVPADLTVGQFVYVVRKRIKL
GAEKAIFVFVKNTLPPTAALMSAIYEEHKDEDGFLYMTYSGENTFGSLTVA

>6 aacu|ASPACDRAFT_114264 | organism=Aspergillus aculeatus ATCC 16872 | Autophagy-related protein [Source:UniProtKB/TrEMBL;Acc:A0A1L9X4B0] | OG6_100707

MRSKFKDEHPFEKRKAEAERIRQKYADRIPVICEKVEKSDIATIDKKKYLVPADLTVGQFVYVIRKRIKLSP
EKAIFIFVDEVLPPTAALMSSIYEEHKDEDGFLYIT

>7 abra|ASPBRDRAFT_124729 | organism=Aspergillus brasiliensis CBS 101740 | Autophagy-related protein [Source:UniProtKB/TrEMBL;Acc:A0A1L9UMS4] | OG6_100707

MRSKFKDEHPFEKRKAEAERIRQKYADRIPVICEKVEKSDIATIDKKKYLVPADLTVGQFVYVIRKRIKLSP
EKAIFIFVDEVLPPTAALMSSIYEEHKDEDGFLYITYSGENTFGDC

>8 acam|P168DRAFT_291575 | organism=Aspergillus campestris IBT 28561 | Autophagy-related protein [Source:UniProtKB/TrEMBL;Acc:A0A2I1CXS6] | OG6_100707

MRSKFKDEHPFEKRKAEAERIRQKYADRIPVICEKVEKSDIATIDKKKYLVPADLTVGQFVYVIRKRIKLSP
EKAIFIFVDEVLPPTAALMSSIYEEHKDEDGFLYITYSGENTFGNC

>9 acaq|I7I50_04093 | organism=Histoplasma capsulatum G186AR | microtubial binding protein | OG6_100707

MRSKFKDEHPFEKRKAEAERIRAKYTDRIPVICEKVEKSDIATIDKKKYLVPADLTVGQFVYVIRKRIKLSPE
KAIFIFVDEVLPPTAALMSSIYEEHKDEDGFLYITYSGENTFGKS

>10 acar|ASPCADRAFT_207553 | organism=Aspergillus carbonarius ITEM 5010 | Autophagy-related protein [Source:UniProtKB/TrEMBL;Acc:A0A1R3RNY2] | OG6_100707

MRSKFKDEHPFEKRKAEAERIRQKYADRIPVICEKVEKSDIATIDKKKYLVPADLTVGQFVYVIRKRIKLSP
EKAIFIFVDEVLPPTAALMSSIYEEHKDEDGFLYITYSGENTFGDC

>11 acla|ACLA_027140 | organism=Aspergillus clavatus NRRL 1 | Autophagy-related protein 8 [Source:UniProtKB/Swiss-Prot;Acc:A1CQS1] | OG6_100707

MRSKFKDEHPFEKRKAEAERIRQKYADRIPVICEKVEKSDIATIDKKKYLVPADLTVGQFVYVIRKRIKLSP
EKAIFIFVDEVLPPTAALMSSIYEEHKDEDGFLYITYSGENTFGDC

>12 acri|SI65_01209 | organism=Aspergillus cristatus GZAAS20.1005 | Autophagy-related protein 8 | OG6_100707

MRSKFKDEHPFEKRKAEAERIRQKYADRIPVICEKVEKSDIATIDKKKYLVPADLTVGQFVYVIRKRIKLSP
EKAIFIFVDEVLPPTAALMSSIYEEHKDEDGFLYITYSGENTFGDC

>13 aeuc|BO83DRAFT_375729 | organism=Aspergillus eucalypticola CBS 122712 | putative autophagic death protein Aut7/IDI-7 | OG6_100707

MRSKFKDEHPFEKRKAEAERIRQKYADRIPVICEKVEKSDIATIDKKKYLVPADLTVGQFVYVIRKRIKLSP
EKAIFIFVDEVLPPTAALMSSIYEEHKDEDGFLYITYSGENTFGDC

>14 afij|BO72DRAFT_11109 | organism=Aspergillus fijiensis CBS 313.89 | light chain 3 | OG6_100707

MRSKFKDEHPFEKRKAEAERIRQKYADRIPVICEKVEKSDIATIDKKKYLVPADLTVGQFVYVIRKRIKLSP
EKAIFIFVDEVLPPTAALMSSIYEEHKDEDGFLYITYSGENTFGDY

>15 afis|NFIA_017280 | organism=Aspergillus fischeri NRRL 181 | autophagic death protein Aut7/IDI-7, putative | OG6_100707

MRSKFKDEHPFEKRKAEAERIRQKYADRIPVICEKVEKSDIATIDKKKYLVPADLTVGQFVYVIRKRIKLSP
EKAIFIFVDEVLPPTAALMSSIYEEHKDEDGFLYITYSGENTFGDC

>16 afla|AFLA_002722 | organism=Aspergillus flavus NRRL3357 | Autophagy-related protein [Source:UniProtKB/TrEMBL;Acc:B8N5B2] | OG6_100707

MRSKFKDEHPFEKRKAEAERIRQKYADRIPVICEKVEKSDIATIDKKKYLVPADLTVGQFVYVIRKRIKLSP
EKAIFIFVDEVLPPTAALMSSIYEEHKDEDGFLYITYSGENTFGDL

>17 afnr|F9C07_4125 | organism=Aspergillus flavus NRRL3357 2020 | microtubule-associated anchor protein | OG6_100707

MRSKFKDEHPFEKRKAEAERIRQKYADRIPVICEKVEKSDIATIDKKKYLVPADLTVGQFVYVIRKRIKLSPEKAIFIFVDEVLPPTAALMSSIYEEHKDEDGFLYITYSGENTFGDL

>18 afub|AFUB_007790 | organism=Aspergillus fumigatus A1163 | Autophagy-related protein [Source:UniProtKB/TrEMBL;Acc:B0XPW3] | OG6_100707

MRSKFKDEHPFEKRKAEAERIRQKYADRIPVICEKVEKSDIATIDKKKYLVPADLTVGQFVYVIRKRIKLSPEKAIFIFVDEVLPPTAALMSSIYEEHKDEDGFLYITYSGENTFGDC

>19 afum-old|Afu1g07470 | organism=Aspergillus fumigatus Af293 (old build 2015-09-27) | Protein involved in autophagy | OG6_100707

MRSKFKDEHPFEKRKAEAERIRQKYADRIPVICEKVEKSDIATIDKKKYLVPADLTVGQFVYVIRKRIKLSPEKAIFIFVDEVLPPTAALMSSIYEEHKDEDGFLYITYSGENTFGDC

>20 afum|Afu1g07470 | organism=Aspergillus fumigatus Af293 | Autophagy-related protein 8 [Source:UniProtKB/Swiss-Prot;Acc:Q4WJ27] | OG6_100707

MRSKFKDEHPFEKRKAEAERIRQKYADRIPVICEKVEKSDIATIDKKKYLVPADLTVGQFVYVIRKRIKLSPEKAIFIFVDEVLPPTAALMSSIYEEHKDEDGFLYITYSGENTFGDC

>21 agla|ASPGLDRAFT_41433 | organism=Aspergillus glaucus CBS 516.65 | Autophagy-related protein [Source:UniProtKB/TrEMBL;Acc:A0A1L9VZW9] | OG6_100707

MRSKFKDEHPFEKRKAEAERIRQKYADRIPVICEKVEKSDIATIDKKKYLVPADLTVGQFVYVIRKRIKLSPEKAIFIFVDEVLPPTAALMSSIYEEHKDEDGFLYITYSGENTFGDC

>22 ahet|BO70DRAFT_374990 | organism=Aspergillus heteromorphus CBS 117.55 | putative autophagic death protein Aut7/IDI-7 | OG6_100707

MRSKFKDEHPFEKRKAEAERIRQKYADRIPVICEKVEKSDIATIDKKKYLVPADLTVGQFVYVIRKRIKLSPEKAIFIFVDEVLPPTAALMSSIYEEHKDEDGFLYITYSGENTFGEC

>23 ahom|BO97DRAFT_479096 | organism=Aspergillus homomorphus CBS 101889 | light chain 3 | OG6_100707

MRSKFKDEHPFEKRKAEAERIRQKYADRIPVICEKVEKSDIATIDKKKYLVPADLTVGQFVYVIRKRIKLSPEKAIFIFVDEVLPPTAALMSSIYEEHKDEDGFLYITYSGENTFGDY

>24 akaw|AKAW_03712 | organism=Aspergillus luchuensis IFO 4308 | Autophagy-related protein [Source:UniProtKB/TrEMBL;Acc:G7XEQ7] | OG6_100707

MRSKFKDEHPFEKRKAEAERIRQKYADRIPVICEKVEKSDIATIDKKKYLVPADLTVGQFVYVIRKRIKLSPEKAIFIFVDEVLPPTAALMSSIYEEHKDEDGFLYITYSGENTFGDC

>25 alen|TMP_alenIFM54703_8590 | organism=Aspergillus lentulus strain IFM 54703 | autophagy-related protein 8 | OG6_100707

MRSKFKDEHPFEKRKAEAERIRQKYADRIPVICEKVEKSDIATIDKKKYLVPADLTVGQFVYVIRKRIKLSPEKAIFIFVDEVLPPTAALMSSIYEEHKDEDGFLYITYSGENTFGDC

>26 aluc|ASPFODRAFT_50283 | organism=Aspergillus luchuensis CBS 106.47 | Autophagy-related protein [Source:UniProtKB/TrEMBL;Acc:A0A1M3T9X8] | OG6_100707

MRSKFKDEHPFEKRKAEAERIRQKYADRIPVICEKVEKSDIATIDKKKYLVPADLTVGQFVYVIRKRIKLSPEKAIFIFVDEVLPPTAALMSSIYEEHKDEDGFLYITYSGENTFGDC

>27 ania|M747DRAFT_299609 | organism=Aspergillus niger ATCC 13496 | Autophagy-related protein [Source:UniProtKB/TrEMBL;Acc:A0A370BPI8] | OG6_100707

MRSKFKDEHPFEKRKAEAERIRQKYADRIPVICEKVEKSDIATIDKKKYLVPADLTVGQFVYVIRKRIKLSPEKAIFIFVDEVLPPTAALMSSIYEEHKDEDGFLYITYADPSDFLLPLFCVHSANSDLLFIRYSGENTFGDC

>28 anid|AN5131 | organism=Aspergillus nidulans FGSC A4 | Autophagy-related protein 8 [Source:UniProtKB/Swiss-Prot;Acc:Q5B2U9] | OG6_100707

MRSKFKDEHPFEKRKAEAERIRAKYADRIPVICEKVEKSDIATIDKKKYLVPADLTVGQFVYVIRKRIKLSPEKAIFIFVDEVLPPTAALMSSIYEEHKDEDGFLYITYSGENTFGDC

>29 anig|An07g10020 | organism=Aspergillus niger CBS 513.88 | Autophagy-related protein 8 [Source:UniProtKB/Swiss-Prot;Acc:A2QPN1] | OG6_100707

MRSKFKDEHPFEKRKAEAERIRQKYADRIPVICEKVEKSDIATIDKKKYLVPADLTVGQFVYVIRKRIKLSP
EKAIFIFVDEVLPPTAALMSSIYEEHKDEDGFLYITYSGENTFGDC

>30 anih|ASPNIDRAFT2_1144328 | organism=Aspergillus niger ATCC 1015 | Autophagy-related protein [Source:UniProtKB/TrEMBL;Acc:G3XZT6] | OG6_100707

MRSKFKDEHPFEKRKAEAERIRQKYADRIPVICEKVEKSDIATIDKKKYLVPADLTVGQFVYVIRKRIKLSP
EKAIFIFVDEVLPPTAALMSSIYEEHKDEDGFLYITYSGENTFGDC

>31 anin|ATCC64974_50530 | organism=Aspergillus niger strain N402 (ATCC64974) | Autophagy-related protein [Source:UniProtKB/TrEMBL;Acc:A0A100ICI1] | OG6_100707

MRSKFKDEHPFEKRKAEAERIRQKYADRIPVICEKVEKSDIATIDKKKYLVPADLTVGQFVYVIRKRIKLSP
EKAIFIFVDEVLPPTAALMSSIYEEHKDEDGFLYITYSGENTFGDC

>32 anov|P174DRAFT_370625 | organism=Aspergillus novofumigatus IBT 16806 | Autophagy-related protein [Source:UniProtKB/TrEMBL;Acc:A0A2I1CA90] | OG6_100707

MRSKFKDEHPFEKRKAEAERIRQKYADRIPVICEKVEKSDIATIDKKKYLVPADLTVGQFVYVIRKRIKLSP
EKAIFIFVDEVLPPTAALMSSIYEEHKDEDGFLYITYSGENTFGDC

>33 aoch|P175DRAFT_0493231 | organism=Aspergillus ochraceoroseus IBT 24754 | Autophagy-related protein [Source:UniProtKB/TrEMBL;Acc:A0A2T5LXB4] | OG6_100707

MRSKFKDEHPFEKRKAEAERIRQKYADRIPVICEKVEKSDIATIDKKKYLVPADLTVGQFMYVIRKRIKLSP
EKAIFIFVDEVLPPTAALMSSIYEEHKDEDGFLYITYSGENTFGDC

>34 aory|AO090012000997 | organism=Aspergillus oryzae RIB40 | Autophagy-related protein 8 [Source:UniProtKB/Swiss-Prot;Acc:Q2UBH5] | OG6_100707

MRSKFKDEHPFEKRKAEAERIRQKYADRIPVICEKVEKSDIATIDKKKYLVPADLTVGQFVYVIRKRIKLSP
EKAIFIFVDEVLPPTAALMSSIYEEHKDEDGFLYITYSGENTFGDL

>35 apar|BDV34DRAFT_82985 | organism=Aspergillus parasiticus CBS 117618 | Autophagy-related protein 8 | OG6_100707

MRSKFKDEHPFEKRKAEAERIRQKYADRIPVICEKVEKSDIATIDKKKYLVPADLTVGQFVYVIRKRIKLSPEKAIFIFVDEVLPPTAALMSSIYEEHKDEDGFLYITYSGENTFGDL

>36 ascl|BO78DRAFT_320841 | organism=Aspergillus sclerotiicarbonarius CBS 121057 | putative autophagic death protein Aut7/IDI-7 | OG6_100707

MRSKFKDEHPFEKRKAEAERIRQKYADRIPVICEKVEKSDIATIDKKKYLVPADLTVGQFVYVIRKRIKLSPEKAIFIFVDEVLPPTAALMSSIYEEHKDEDGFLYITYSGENTFGDC

>37 aste|P170DRAFT_478339 | organism=Aspergillus steynii IBT 23096 | Autophagy-related protein [Source:UniProtKB/TrEMBL;Acc:A0A2I2FXL5] | OG6_100707

MRSKFKDEHPFEKRKAEAERIRQKYADRIPVICEKVEKSDIATIDKKKYLVPADLTVGQFVYVIRKRIKLSPEKAIFIFVDEVLPPTAALMSSIYEEHKDEDGFLYITYSGENTFGEC

>38 asyd|ASPSYDRAFT_47609 | organism=Aspergillus sydowii CBS 593.65 | Autophagy-related protein [Source:UniProtKB/TrEMBL;Acc:A0A1L9TAJ4] | OG6_100707

MRSKFKDEHPYEKRKAEAERIRAKYADRIPVICEKVEKSDIATIDKKKYLVPADLTVGQFVYVIRKRIKLSPEKAIFIFVDEVLPPTAALMSSIYEEHKDEDGFLYITYSGENTFGDC

>39 atan|EYZ11_009758 | organism=Aspergillus tanneri NIH1004 | Autophagy-related protein [Source:UniProtKB/TrEMBL;Acc:A0A4S3J797] | OG6_100707

MRSKFKDEHPFEKRKAEAERIRQKYADRIPVICEKVEKSDIATIDKKKYLVPADLTVGQFVYVIRKRIKLSPEKAIFIFVDEVLPPTAALMSSIYEEHKDEDGFLYITYSGENTFGDC

>40 ater|ATEG_10180 | organism=Aspergillus terreus NIH2624 | Autophagy-related protein 8 [Source:UniProtKB/Swiss-Prot;Acc:Q0C804] | OG6_100707

MRSKFKDEHPFEKRKAEAERIRQKYADRIPVICEKVEKSDIATIDKKKYLVPADLTVGQFVYVIRKRIKLSPEKAIFIFVDEVLPPTAALMSSIYEEHKDEDGFLYITYSGENTFGDC

>41 athe|CDV56_106253 | organism=Aspergillus thermomutatus strain HMR AF 39 | Autophagy-related protein [Source:UniProtKB/TrEMBL;Acc:A0A397H1D5] | OG6_100707

MRSKFKDEHPFEKRKAEAERIRQKYADRIPVICEKVEKSDIATIDKKKYLVPADLTVGQFVYVIRKRIKLSP
EKAIFIFVDEVLPPTAALMSSIYEEHKDEDGFLYITYSGENTFGDC

>42 atub|ASPTUDRAFT_192670 | organism=Aspergillus tubingensis CBS 134.48 | Autophagy-related protein [Source:UniProtKB/TrEMBL;Acc:A0A1L9MZG2] | OG6_100707

MRSKFKDEHPFEKRKAEAERIRQKYADRIPVICEKVEKSDIATIDKKKYLVPADLTVGQFVYVIRKRIKLSP
EKAIFIFVDEVLPPTAALMSSIYEEHKDEDGFLYITYSGENTFGDC

>43 auva|BO82DRAFT_358536 | organism=Aspergillus uvarum CBS 121591 | light chain 3 | OG6_100707

MRSKFKDEHPFEKRKAEAERIRQKYADRIPVICEKVEKSDIATIDKKKYLVPADLTVGQFVYVIRKRIKLSP
EKAIFIFVDEVLPPTAALMSSIYEEHKDEDGFLYITYSGENTFGDY

>44 aver|ASPVEDRAFT_140090 | organism=Aspergillus versicolor CBS 583.65 | Autophagy-related protein [Source:UniProtKB/TrEMBL;Acc:A0A1L9PXW5] | OG6_100707

MRSKFKDEHPYEKRKAEAERIRAKYADRIPVICEKVEKSDIATIDKKKYLVPADLTVGQFVYVIRKRIKLSPE
KAIFIFVDEVLPPTAALMSSIYEEHKDEDGFLYITYSGENTFGDC

>45 awen|ASPWEDRAFT_40953 | organism=Aspergillus wentii DTO 134E9 | Autophagy-related protein [Source:UniProtKB/TrEMBL;Acc:A0A1L9RLK1] | OG6_100707

MRSKFKDEHPFEKRKAEAERIRQKYADRIPVICEKVEKSDIATIDKKKYLVPADLTVGQFVYVIRKRIKLSP
EKAIFIFVDEVLPPTAALMSSIYEEHKDEDGFLYITYSGENTFGDC

>46 bfuc|Bcin02g02570 | organism=Botrytis cinerea B05.10 | Autophagy-related protein [Source:UniProtKB/TrEMBL;Acc:A0A384J8Q5] | OG6_100707 (#006633)

MRSKFKDEHPFEKRKAEAERIRQKYNDRIPVICEKVEKSDIATIDKKKYLVPSDLTVGQFVYVIRKRIKLSP
EKAIFIFVDEVLPPTAALMSSIYEEHKDEDGFLYISYSGENTFGEALEEAN

>47 cabb|C1_05700W_B | organism=Candida albicans SC5314_B | Putative autophagosome protein; acts synergistically with Ysy6p to regulate unfolded protein response and mitochondrial | OG6_100707

MRSQFKDEHPFEKRQAEAARIAQRFKDRVPVICEKVENSDIPEIDKRKYLVPVDLTVGQFVYVIRKRIKLP
SEKAIFIFVNDILPPTAALISTIYEEHKDEDGFLYVLYSGENTFGEKLAIDISSLDFSDIPDYV

>48 calb|C1_05700W_A | organism=Candida albicans SC5314 | Autophagy-related protein 8 [Source:UniProtKB/Swiss-Prot;Acc:P0C075] | OG6_100707

MRSQFKDEHPFEKRQAEAARIAQRFKDRVPVICEKVENSDIPEIDKRKYLVPVDLTVGQFVYVIRKRIKLP
SEKAIFIFVNDILPPTAALISTIYEEHKDEDGFLYVLYSGENTFGEKLAIDISSLDFSDIPDYV

>49 calw|CAWG_00835 | organism=Candida albicans WO-1 | Autophagy-related protein [Source:UniProtKB/TrEMBL;Acc:C4YE81] | OG6_100707

MRSQFKDEHPFEKRQAEAARIAQRFKDRVPVICEKVENSDIPEIDKRKYLVPVDLTVGQFVYVIRKRIKLP
SEKAIFIFVNDILPPTAALISTIYEEHKDEDGFLYVLYSGENTFGEKLAIDISSLDFSDIPDYV

>50 caub|CJI96_0003871 | organism=Candida auris strain B11220 | Autophagy-related protein [Source:UniProtKB/TrEMBL;Acc:A0A510P0T5] | OG6_100707

MRSQFKDENPFDKRKSESTRILQRFKDRLPVICEKVENSDIQEIDKRKYLVPGDLTVGQFVYVIRKRIKLP
SEKAIFIFVNDILPPTAALMSTVYEEHKDEDGFLYVLYSGENTFGEIEGVEEVAL

>51 cauc|CJJ09_004272 | organism=Candida auris strain B11245 | Autophagy-related protein [Source:UniProtKB/TrEMBL;Acc:A0A510P0T5] | OG6_100707

MRSQFKDENPFDKRKSESTRILQRFKDRLPVICEKVENSDIQEIDKRKYLVPGDLTVGQFVYVIRKRIKLP
SEKAIFIFVNDILPPTAALMSTVYEEHKDEDGFLYVLYSGENTFGEIEGVEEVAL

>52 caur|B9J08_005081 | organism=Candida auris strain B8441 | Autophagy-related protein [Source:UniProtKB/TrEMBL;Acc:A0A510P0T5] | OG6_100707

MRSQFKDENPFDKRKSESTRILQRFKDRLPVICEKVENSDIQEIDKRKYLVPGDLTVGQFVYVIRKRIKLP
SEKAIFIFVNDILPPTAALMSTVYEEHKDEDGFLYVLYSGENTFGEIEGVEEVAL

>53 cauu|QG37_01246 | organism=Candida auris strain 6684 | Autophagy-related protein [Source:UniProtKB/TrEMBL;Acc:A0A510P0T5] | OG6_100707

MRSQFKDENPFDKRKSESTRILQRFKDRLPVICEKVENSDIQEIDKRKYLVPGDLTVGQFVYVIRKRIKLPSEKAIFIFVNDILPPTAALMSTVYEEHKDEDGFLYVLYSGENTFGEIEGVEEVAL

MF34

>54 cauy|CJJ07_000435 | organism=Candida auris strain B11243 | Autophagy-related protein [Source:UniProtKB/TrEMBL;Acc:A0A510P0T5] | OG6_100707

MRSQFKDENPFDKRKSESTRILQRFKDRLPVICEKVENSDIQEIDKRKYLVPGDLTVGQFVYVIRKRIKLPSEKAIFIFVNDILPPTAALMSTVYEEHKDEDGFLYVLYSGENTFGEIEGVEEVAL

>55 cauz|CJI97_005166 | organism=Candida auris strain B11221 | Autophagy-related protein [Source:UniProtKB/TrEMBL;Acc:A0A510P0T5] | OG6_100707

MRSQFKDENPFDKRKSESTRILQRFKDRLPVICEKVENSDIQEIDKRKYLVPGDLTVGQFVYVIRKRIKLPSEKAIFIFVNDILPPTAALMSTVYEEHKDEDGFLYVLYSGENTFGEIEGVEEVAL

>56 ccfg|D1P53_002899 | organism=Cryptococcus cf. gattii MF34 | unknown | OG6_100707

MVRSKFKDEHPFDKRKAEAERIRQKYQDRIPVICEKAEKSDIPTIDKKKYLVPADLTVGQKRIKLAPEKAIFIFVDDILPPTAALMSSIYDEHKDEDGFLYVLYASENTFGDLEQYAISE

>57 cdcb|L204_06311 | organism=Cryptococcus depauperatus CBS 7855 | microtubule binding protein | OG6_100707

MVRSKFKDEHPFDKRKAEAERIRQKYQDRIPVICEKAEKSDIPTIDKKKYLVPADLTVGQFVYVIRKRIKLAPEKAIFIFVDDILPPTAALMSSIYDEHKDEDGFLYVLYASENTFGDLEQYAVRE

>58 cdep|L203_02047 | organism=Cryptococcus depauperatus CBS 7841 | microtubule binding protein | OG6_100707

MVRSKFKDEHPFDKRKAEAERIRQKYQDRIPVICEKAEKSDIPTIDKKKYLVPADLTVGQFVYVIRKRIKLAPEKAIFIFVDDILPPTAALMSSIYDEHKDEDGFLYVLYASENTFGDLEQYAVRE

>59 cdeu|CNBG_1122 | organism=Cryptococcus gattii VGII R265 | microtubule binding protein | OG6_100707

MVRSKFKDEHPFDKRKAEAERIRQKYQDRIPVICEKAEKSDIPTIDKKKYLVPADLTVGQFVYVIRKRIKLA PEKAIFIFVDDILPPTAALMSSIYDEHKDEDGFLYVLYASENTFGDLEQYAIAE

>60 cdub|CD36_05380 | organism=Candida dubliniensis CD36 | CD36_05380 | OG6_100707

MRSQFKDEHPFEKRQAEAARIAQRFKDRVPVICEKVENSDIPEIDKRKYLVPVDLTVGQFVYVIRKRIKLP SEKAIFIFVNDILPPTAALISTIYEEHKDEDGFLYVLYSGENTFGEKIAIDISSLDYSDIPDYV

>61 cduo|CXQ87_003838 | organism=Candida duobushaemulonis strain B09383 | Autophagy-related protein [Source:UniProtKB/TrEMBL;Acc:A0A2V1AC90] | OG6_100707

MRSQFKDENSFEKRKSESTRILQRFKDRLPVICEKVENSDIQEIDKRKYLVPGDLTVGQFVYVIRKRIKLP SEKAIFIFVNDILPPTAALMSTVYEEHKDEDGFLYVLYSGENTFGAIEGVEEIHDVEEVEL

>62 cgac|I314_00045 | organism=Cryptococcus gattii CA1873 | unknown | OG6_100707

MVRSKFKDEHPFDKRKAEAERIRQKYQDRIPVICEKAEKSDIPTIDKKKYLVPADLTVGQFVYVIRKRIKLA PEKAIFIFVDDILPPTAALMSSIYDEHKDEDGFLYVLYASENTFGDLEQYAISE

>63 cgae|I306_03565 | organism=Cryptococcus gattii EJB2 | Autophagy-related protein [Source:UniProtKB/TrEMBL;Acc:A0A0D0Y840] | OG6_100707

MVRSKFKDEHPFDKRKAEAERIRQKYQDRIPVICEKAEKSDIPTIDKKKYLVPADLTVGQFVYVIRKRIKLA PEKAIFIFVDDILPPTAALMSSIYDEHKDEDGFLYVLYASENTFGDLEQYAISE

>64 cgai|I308_05864 | organism=Cryptococcus gattii VGIV IND107 | Autophagy-related protein [Source:UniProtKB/TrEMBL;Acc:A0A0D0YLV0] | OG6_100707

MVRSKFKDEHPFDKRKAEAERIRQKYQDRIPVICEKAEKSDIPTIDKKKYLVPADLTVGQFVYVIRKRIKLA PEKAIFIFVDDILPPTAALMSSIYDEHKDEDGFLYVLYASENTFGDLEQYAISE

>65 cgan|I311_03653 | organism=Cryptococcus gattii NT-10 | unknown | OG6_100707

MVRSKFKDEHPFDKRKAEAERIRQKYQDRIPVICEKAEKSDIPTIDKKKYLVPADLTVGQFVYVIRKRIKLA PEKAIFIFVDDILPPTAALMSSIYDEHKDEDGFLYVLYASENTFGDLEQYAISE

>66 cgat|CGB_A9330C | organism=Cryptococcus gattii WM276 | Autophagy-related protein [Source:UniProtKB/TrEMBL;Acc:E6QYR7] | OG6_100707

MVRSKFKDEHPFDKRKAEAERIRQKYQDRIPVICEKAEKSDIPTIDKKKYLVPADLTVGQFVYVIRKRIKLA PEKAIFIFVDDILPPTAALMSSIYDEHKDEDGFLYVLYASENTFGDLEQYAISE

>67 chae|CXQ85_000290 | organism=Candida haemulonis B11899 | Autophagy-related protein [Source:UniProtKB/TrEMBL;Acc:A0A2V1ASY9] | OG6_100707

MRSQFKDENSFEKRKSESTRILQRFKDRLPVICEKVENSDIQEIDKRKYLVPGDLTVGQFVYVIRKRIKLP SEKAIFIFVNDILPPTAALMSTVYEEHKDEDGFLYVLYSGENTFGSIEGVEEVTEESL

>68 cimh|CIHG_04398 | organism=Coccidioides immitis H538.4 | IDI-7 | OG6_100707

MRSKFKDEHPFEKRKAEAERIRQKYADRIPVICEKVEKSDIATIDKKKYLVPADLTVGQFVYVIRKRIKLSP EKAIFIFVDEVLPPTAALMSSIYEEHKDDDGFLYITYSGENTFGN

>69 cimm|CIMG_02508 | organism=Coccidioides immitis RS | Autophagy-related protein 8 [Source:UniProtKB/Swiss-Prot;Acc:Q1E4K5] | OG6_100707

MRSKFKDEHPFEKRKAEAERIRQKYADRIPVICEKVEKSDIATIDKKKYLVPADLTVGQFVYVIRKRIKLSP EKAIFIFVDEVLPPTAALMSSIYEEHKDDDGFLYITYSGENTFGN

>70 cimw|DIZ76_010280 | organism=Coccidioides immitis WA_211 | ubiquitin-like protein atg8 | OG6_100707

MRSKFKDEHPFEKRKAEAERIRQKYADRIPVICEKVEKSDIATIDKKKYLVPADLTVGQFVYVIRKRIKLSP EKAIFIFVDEVLPPTAALMSSIYEEHKDDDGFLYITYSGENTFGN

>71 cmet|I9W82_000976 | organism=Candida metapsilosis BP57 | ATG8 | OG6_100707

MRSQFKDEHPFEKRQAEAARIAQRFKDRVPVICEKVENSDIPEIDKRKYLVPVDLTVGQFVYVIRKRIKLPSEKAIFIFVNDILPPTAALISTIYEEHKDEDGFLYVLYSGENTFGEKVPINLSELDFTDLPEDL

>72 cnek|CKF44_00816 | organism=Cryptococcus neoformans var. grubii KN99 | Autophagy-related protein [Source:UniProtKB/TrEMBL;Acc:A0A120HYU7] | OG6_100707

MVRSKFKDEHPFDKRKAEAERIRQKYQDRIPVICEKAEKSDIPTIDKKKYLVPADLTVGQFVYVIRKRIKLAPEKAIFIFVDDILPPTAALMSSIYDEHKDEDGFLYVLYASENTFGDLEQYAISE

>73 cneo-old|CNA07930 | organism=Cryptococcus neoformans var. neoformans JEC21 (old build 2016-06-16) | microtubule binding protein, putative | OG6_100707

MVRSKFKDEHPFDKRKAEAERIRQKYQDRIPVICEKAEKSDIPTIDKKKYLVPADLTVGQFVYVIRKRIKLAPEKAIFIFVDDILPPTAALMSSIYDEHKDEDGFLYVLYASENTFGDLEQYAISE

>74 cneo|CNA07930 | organism=Cryptococcus neoformans var. neoformans JEC21 | Autophagy-related protein 8 [Source:UniProtKB/Swiss-Prot;Acc:P0CO54] | OG6_100707

MVRSKFKDEHPFDKRKAEAERIRQKYQDRIPVICEKAEKSDIPTIDKKKYLVPADLTVGQFVYVIRKRIKLAPEKAIFIFVDDILPPTAALMSSIYDEHKDEDGFLYVLYASENTFGDLEQYAISE

>75 cnep|CNBA7760 | organism=Cryptococcus neoformans var. neoformans B-3501A | Autophagy-related protein 8 [Source:UniProtKB/Swiss-Prot;Acc:P0CO55] | OG6_100707

MVRSKFKDEHPFDKRKAEAERIRQKYQDRIPVICEKAEKSDIPTIDKKKYLVPADLTVGQFVYVIRKRIKLAPEKAIFIFVDDILPPTAALMSSIYDEHKDEDGFLYVLYASENTFGDLEQYAISE

>76 cneq|CNAG_00816 | organism=Cryptococcus neoformans var. grubii H99 | Autophagy-related protein [Source:UniProtKB/TrEMBL;Acc:J9VF50] | OG6_100707

MVRSKFKDEHPFDKRKAEAERIRQKYQDRIPVICEKAEKSDIPTIDKKKYLVPADLTVGQFVYVIRKRIKLAPEKAIFIFVDDILPPTAALMSSIYDEHKDEDGFLYVLYASENTFGDLEQYAISE

>77 cnev|LQV05_000780 | organism=Cryptococcus neoformans strain:VNII | Autophagy-related protein [Source:UniProtKB/TrEMBL;Acc:A0A120HYU7] | OG6_100707

MVRSKFKDEHPFDKRKAEAERIRQKYQDRIPVICEKAEKSDIPTIDKKKYLVPADLTVGQFVYVIRKRIKLA PEKAIFIFVDDILPPTAALMSSIYDEHKDEDGFLYVLYASENTFGDLEQYAISE

>78 cpac|CPAR2_107460 | organism=Candida parapsilosis CDC317 | Autophagy-related protein [Source:UniProtKB/TrEMBL;Acc:G8B8C6] | OG6_100707

MRSQFKDEHPFEKRQAEAARIAQRFKDRVPVICEKVENSDIPEIDKRKYLVPVDLTVGQFVYVIRKRIKLP SEKAIFIFVNDILPPTAALISTIYEEHKDEDGFLYVLYSGENTFGEKVPIDLTDLDFNNLSEDL

>79 cpos|CPC735_038040 | organism=Coccidioides posadasii C735 delta SOWgp | Autophagy-related protein [Source:UniProtKB/TrEMBL;Acc:C5P2J5] | OG6_100707

MRSKFKDEHPFEKRKAEAERIRQKYADRIPVICEKVEKSDIATIDKKKYLVPADLTVGQFVYVIRKRIKLSP EKAIFIFVDEVLPPTAALMSSIYEEHKDDDGFLYITYSGENTFGN

>80 cprm|CPAG_00282 | organism=Coccidioides posadasii RMSCC 3488 | Autophagy-related protein [Source:UniProtKB/TrEMBL;Acc:A0A0J6F4M8] | OG6_100707

MRSKFKDEHPFEKRKAEAERIRQKYADRIPVICEKVEKSDIATIDKKKYLVPADLTVGQFVYVIRKRIKLSP EKAIFIFVDEVLPPTAALMSSIYEEHKDDDGFLYITYSGENTFGN

>81 cpse|C7M61_001373 | organism=Candida pseudohaemulonii strain B12108 | Autophagy-related protein [Source:UniProtKB/TrEMBL;Acc:A0A2P7YUD1] | OG6_100707

MRSQFKDENSFEKRKSESTRILQRFKDRLPVICEKVENSDIQEIDKRKYLVPGDLTVGQFVYVIRKRIKLP SEKAIFIFVNDILPPTAALMSTVYEEHKDEDGFLYVLYSGENTFGAIDGVEEIHELEEVEL

>82 cpsi|CPSG_00371 | organism=Coccidioides posadasii str. Silveira | Autophagy-related protein [Source:UniProtKB/TrEMBL;Acc:E9CRP5] | OG6_100707

MRSKFKDEHPFEKRKAEAERIRQKYADRIPVICEKVEKSDIATIDKKKYLVPADLTVGQFVYVIRKRIKLSP EKAIFIFVDEVLPPTAALMSSIYEEHKDDDGFLYITYSGENTFGN

>83 cpsl|D8B26_000850 | organism=Coccidioides posadasii strain Silveira 2022 | ubiquitin-like protein atg8 | OG6_100707

MRSKFKDEHPFEKRKAEAERIRQKYADRIPVICEKVEKSDIATIDKKKYLVPADLTVGQFVYVIRKRIKLSP EKAIFIFVDEVLPPTAALMSSIYEEHKDDDGFLYITYSGENTFGN

>84 ctmy|CTMYA2_026980 | organism=Candida tropicalis MYA-3404 2020 | unknown | OG6_100707

MRSQFKDEHPFEKRQAEATRIAQRFKDRVPVICEKVENSDIPEIDKRKYLVPVDLTVGQFVYVIRKRIKLP SEKAIFIFVNDILPPTAALISTIYEEHKDEDGFLYVLYSGENTFGEQVPIDLSTVDFSDIPKDV

>85 hcag|I7I48_04855 | organism=Histoplasma capsulatum G217B | microtubial binding protein | OG6_100707

MRSKFKDEHPFEKRKAEAERIRAKYTDRIPVICEKVEKSDIATIDKKKYLVPADLTVGQFVYVIRKRIKLSPE KAIFIFVDEVLPPTAALMSSIYEEHKDEDGFLYITYSGENTFGKS

>86 hcah|I7I53_01784 | organism=Histoplasma capsulatum H88 | microtubial binding protein | OG6_100707

MRSKFKDEHPFEKRKAEAERIRAKYTDRIPVICEKVEKSDIATIDKKKYLVPADLTVGQFVYVIRKRIKLSPE KAIFIFVDEVLPPTAALMSSIYEEHKDEDGFLYITYSGENTFGKS

>87 hcap|HCDG_08714 | organism=Histoplasma capsulatum H143 | Autophagy-related protein [Source:UniProtKB/TrEMBL;Acc:C6HQP2] | OG6_100707

MRSKFKDEHPFEKRKAEAERIRAKYTDRIPVICEKVEKSDIATIDKKKYLVPADLTVGQFVYVIRKRIKLSPE KAIFIFVDEVLPPTAALMSSIYEEHKDEDGFLYITYSGENTFGKS

>88 hcga|I7I52_05001 | organism=Histoplasma capsulatum G184AR | microtubial binding protein | OG6_100707

MRSKFKDEHPFEKRKAEAERIRAKYTDRIPVICEKVEKSDIATIDKKKYLVPADLTVGQFVYVIRKRIKLSPE KAIFIFVDEVLPPTAALMSSIYEEHKDEDGFLYITYSGENTFGKS

>89 mcic|HMPREF1544_01983 | organism=Mucor circinelloides 1006PhL | Autophagy-related protein [Source:UniProtKB/TrEMBL;Acc:S2K6Q3] | OG6_100707

MTTTSKPASFKSRHSFDARLKESKRILSKYPDRIPIICEKVEGNNIARMAKQKYLIPSDICLGQFIYSIRKHIRLSEEKAIYMYIGGNIPAVSESLASLYAQYKDQDGFLYLNYAGENTFGCAL

>90 mcic|HMPREF1544_11186 | organism=Mucor circinelloides 1006PhL | Autophagy-related protein [Source:UniProtKB/TrEMBL;Acc:S2IXV1] | OG6_100707

MRSKFKDEHPFEKRKAEAERIRQKYPDRIPVICEKVEKSDIPTIDKKKYLVPADLTVGQFVYVIRKRIKLSPEKAIFIFVNEILPPTAALMSAIYEEHKDEDGFLYITYSGENTFGF

>91 mcir|MUCCIDRAFT_107156 | organism=Mucor lusitanicus CBS 277.49 | Autophagy-related protein [Source:UniProtKB/TrEMBL;Acc:A0A162QXX6] | OG6_100707

MATLSKPASSFKTRHSFDARLKESKRILNKYPDRIPIICEKVEGNSIARMSKQKYLIPSDICLGQFIYSIRKNIKLSEEKVIYMYIGGSIPTISESLSSLYTQYKDEDGFLYLSYAGENTFGAL

>92 mcir|MUCCIDRAFT_155732 | organism=Mucor lusitanicus CBS 277.49 | Autophagy-related protein [Source:UniProtKB/TrEMBL;Acc:A0A162TII1] | OG6_100707

MRSKFKDEHPFEKRKAEAERIRQKYPDRIPVICEKVEKSDIPTIDKKKYLVPADLTVGQFVYVIRKRIKLSPEKAIFIFVNEILPPTAALMSAIYEEHKDEDGFLYITYSGENTFGF

>93 mfru|MFRU_001g04410 | organism=Monilinia fructicola CPMC6 | unknown | OG6_100707 (#006633)

MRSKFKDEHPFEKRKAEAERIRQKYSDRIPVICEKVEKSDIATIDKKKYLVPSDLTVGQFVYVIRKRIKLSPEKAIFIFVDEVLPPTAALMSSIYEEHKDEDGFLYISYSGENTFGGALEEAN

>94 pkud|C5L36_0C08630 | organism=Pichia kudriavzevii strain CBS573 | Autophagy-related protein [Source:UniProtKB/TrEMBL;Acc:A0A2U9R6D1] | OG6_100707

MRHENCWQPVQSTNTQSATKHCYLSILEKRKEESMRIRSKFQDRIPVICERVEDSDIPNIDKRKYLVPSDLTVGQFVYVIRRRIHLPSEKAIFIFVKDILPPTTALMSTIYDQYKDEDGFLYIVYSGENTFGELCQV

>95 sscl-old|SS1G_01602 | organism=Sclerotinia sclerotiorum 1980 UF-70 (old build 2015-03-23) | hypothetical protein | OG6_100707 (#006633)

MRSKFKDEHPFEKRKAEAERIRQKYSDRIPVICEKVEKSDIATIDKKKYLVPSDLTVGQFVYVIRKRIKLSP
EKAIFIFVDEVLPPTAALMSSIYEEHKDEDGFLYISYSGENTFGEALEEAN

>96 sscl|sscle_01g007550 | organism=Sclerotinia sclerotiorum 1980 UF-70 | Autophagy-related protein [Source:UniProtKB/TrEMBL;Acc:A0A1D9PTE5] | OG6_100707 (#006633)

MRSKFKDEHPFEKRKAEAERIRQKYSDRIPVICEKVEKSDIATIDKKKYLVPSDLTVGQFVYVIRKRIKLSP
EKAIFIFVDEVLPPTAALMSSIYEEHKDEDGFLYISYSGENTFGEALEEAN

>97 sp|O95166|GBRAP_HUMAN Gamma-aminobutyric acid receptor-associated protein OS=Homo sapiens OX=9606 GN=GABARAP PE=1 SV=1
MKFVYKEEHPFEKRRSEGEKIRKKYPDRVPVIVEKAPKARIGDLDKKKYLVPSDLTVGQFYFLIRKRIHLR
AEDALFFFVNNVIPPTSATMGQLYQEHHEEDFFLYIAYSDESVYGL

>98 sp|Q9H0R8|GBRL1_HUMAN Gamma-aminobutyric acid receptor-associated protein-like 1 OS=Homo sapiens OX=9606 GN=GABARAPL1 PE=1 SV=1
MKFQYKEDHPFEYRKKEGEKIRKKYPDRVPVIVEKAPKARVPDLDKRKYLVPSDLTVGQFYFLIRKRIHLR
PEDALFFFVNNTIPPTSATMGQLYEDNHEEDYFLYVAYSDESVYGK

>99 sp|P60520|GBRL2_HUMAN Gamma-aminobutyric acid receptor-associated protein-like 2 OS=Homo sapiens OX=9606 GN=GABARAPL2 PE=1 SV=1
MKWMFKEDHSLEHRCVESAKIRAKYPDRVPVIVEKVSGSQIVDIDKRKYLVPSDITVAQFMWIIRKRIQL
PSEKAIFLFVDKTVPQSSLTMGQLYEKEKDEDGFLYVAYSGENTFGF

>100 sp|Q9BY60|GBRL3_HUMAN Gamma-aminobutyric acid receptor-associated protein-like 3 OS=Homo sapiens OX=9606 GN=GABARAPL3 PE=2 SV=1
MKFQYKEVHPFEYRKKEGEKIRKKYPDRVPLIVEKAPKARVPDLDRRKYLVPSDLTDGQFYLLIRKRIHLR
PEDALFFFVNNTIPPTSATMGQLYEDSHEEDDFLYVAYSNESVYGK
